# The Variation of White Matter Connectome After Surgery Revealed Factors Affecting Supplementary Syndrome Recovery Time in Low‐Grade Glioma Patients

**DOI:** 10.1111/cns.70426

**Published:** 2025-05-10

**Authors:** Shengyu Fang, Yuzhe Li, Shimeng Weng, Jiahan Dong, Jiangwei Wang, Zhong Zhang, Xing Fan, Yinyan Wang, Wenbin Ma, Tao Jiang

**Affiliations:** ^1^ Department of Neurosurgery, Beijing Tiantan Hospital Capital Medical University Beijing China; ^2^ Chinese Academy of Medical Sciences and Peking Union Medical College Beijing China; ^3^ Beijing Neurosurgical Institute Capital Medical University Beijing China; ^4^ Research Unit of Accurate Diagnosis, Treatment, and Translational Medicine of Brain Tumors Chinese Academy of Medical Sciences Beijing China

**Keywords:** glioma, supplementary motor area syndrome, topological properties, white matter connectome

## Abstract

**Objective:**

Supplementary motor area (SMA) syndrome is a common complication after SMA glioma resection. The compensatory mechanism of the structural sensorimotor network (SMN) and the factors influencing the recovery time of SMA syndrome have not been investigated.

**Methods:**

Pre‐ and postoperative diffusion tensor images of 42 low‐grade glioma patients with SMA syndrome were processed to construct white matter connectomes. Patients were classified into fast and slow‐recovery groups according to whether postoperative motor disorder recovers within 7 days. Fiber counts between nodes and graph theory topological properties were calculated. The shortest distance from the surgical region to the corticospinal tract (*d*_CST_) and the upper limb region of Brodmann area 4 (A4ul) was measured to find correlations with recovery time. Cox regressions were conducted to identify factors associated with SMA syndrome recovery time. A general linear model was formed using significant factors in multivariate Cox analysis to predict recovery time.

**Results:**

Decrease of fiber number between lesioned‐hemispheric A4ul and contralateral SMN is correlated with prolongation of recovery time. Compared with the slow‐recovery group, a higher increase of nodal degree centrality and nodal efficiency of ipsilateral A4ul was found in the fast‐recovery group (nodal efficiency: left pre‐op: 0.182 ± 0.009, left post‐op: 0.231 ± 0.008, *p* < 0.0001; right pre‐op: 0.157 ± 0.021, right post‐op: 0.195 ± 0.018, *p* = 0.0011); (nodal degree centrality: left pre‐op: 1.985 ± 0.166; left post‐op: 3.195 ± 0.230, *p* < 0.0001; right pre‐op: 1.620 ± 0.389; right post‐op: 2.411 ± 0.452, *p* = 0.0005). Multivariate Cox analysis indicated that the increase in nodal efficiency of A4ul and *d*_CST_ were protective factors for SMA syndrome recovery time. A significant negative correlation between the predict score and recovery time was found in the left lesion group (*r* = −0.756, *p* < 0.0001), and the same trend was found in the right lesion group (*r* = −0.531, *p* = 0.076).

**Conclusions:**

This study revealed an increase in lesioned‐hemispheric A4ul nodal efficiency and long *d*_CST_ as protective factors in SMA syndrome recovery. A decrease in the number of interhemispheric fibers connecting lesioned‐hemispheric A4ul to nodes on the contralateral hemisphere was correlated with the long recovery time of SMA syndrome.

## Graphical abstract

Analysis of the structural network of the patients with SMA glioma revealed that the ipsilateral upper limb region of Brodmann area 4 (A4ul) and the surgical region to the corticospinal tract (CST) were related to SMA syndrome recovery time.

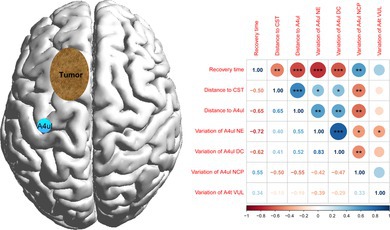

**Abbreviations**
A4ll: lower limb region of Brodmann area 4
A4t: trunk region of Brodmann area 4
A4ul: upper limb region of Brodmann area 4
A6m: medial of Brodmann area 6
AC: awaken craniotomy
CST: corticospinal tract
*d*_A4ul_
: shortest distance from surgical region to A4ul
*d*_CST_
: shortest distance from surgical region to corticospinal tract
DTI: diffusion tensor imaging
FN: fiber numbers
HR: hazard ratio.
rs‐fMRI: resting‐state fMRI
SMA: Supplementary motor area
SMN: sensorimotor network

## 1 Introduction

Supplementary motor area (SMA) syndrome often occurs after SMA glioma resection [1, 2]. It is defined by global or partial akinesia within 24 h of surgery. A previous study showed that more than 90% of SMA tumor resections were followed by SMA syndrome [3]. After medial prefrontal lobe surgery, patients often exhibit akinesia affecting the contralateral side in the immediate postoperative phase, characterized by mild hypotonia and reflexes ranging from reduced to normal [4, 5]. When dominant frontal lobe structures are involved, this may be accompanied by reduced spontaneous speech with preserved language comprehension [6]. Performing awake craniotomy (AC) for SMA glioma patients can provide instant monitoring of motor function, but the SMA syndrome could not be completely avoided [7]. Laplane et al. described three stages of SMA syndrome [6]: (1) in the immediate postoperative period, global akinesia, mild hypotonia, and reduced stretch reflexes in the contralateral hemicorpus; patients with dominant frontal injury may have reduction in spontaneous speech [4, 5, 6]; (2) in the progressive recovery period, rapid recovery of muscular strength and tonus is expected within a few days; (3) full recovery is expected after weeks to months; some patients may continue to suffer from mild motor and speech dysfunction. Nakajima et al. found that the severity of akinesia on the seventh postoperative day correlated positively with the time to total clinical recovery [8].

The development of glioma induces neuroplasticity of adjacent brain tissue [9]. SMA is part of the sensorimotor network (SMN). The SMN is a functional network in the brain responsible for integrating sensory inputs (e.g., tactile and proprioceptive information) and motor outputs. By coordinating multiple brain regions, it facilitates the transformation from perception to action, underpinning fundamental motor control, complex skill learning, and adaptive behavior. The development of glioma in this area is concurrent with SMN compensation and reconstruction. This plays an important role in the recovery of SMA syndrome [10]. After surgery, SMN reconstruction is activated again due to the structural and functional damage [11]. Vassal et al. have reported evidence from resting‐state fMRI (rs‐fMRI) studies indicating that interhemispheric connectivity correlates with SMA syndrome recovery [12]. Furthermore, rs‐fMRI studies have indicated that damage to the lesioned‐hemispheric primary motor cortex and cingulate gyrus is associated with SMA syndrome occurrence [1, 13, 14]. The preliminary exploration of motor function network compensation in the context of SMA syndrome has been conducted through rs‐fMRI studies. The white structural matter of the brain connects different parts of the cortex, forming the structural network of the brain. The structural network is fundamental to understanding function execution. However, the structural network reconstruction following SMA syndrome remains unknown. To explore the interhemispheric and intrahemispheric compensation mechanisms following SMA syndrome, results from rs‐fMRI functional network studies must be combined with structural network studies, which are still scarce. The exploration of the structural network compensation contributes to the understanding of SMA syndrome and may reveal factors affecting the recovery time of SMA glioma patients, thus achieving better clinical outcomes. Therefore, we studied the structural network in low‐grade glioma patients with postoperative SMA syndrome after AC to explore the compensation mechanism of SMA syndrome. Preoperative and paired postoperative diffusion tensor imaging (DTI) tractography were used to investigate postoperative SMN reconstruction. The SMN reconstruction during SMA syndrome recovery and factors affecting the recovery time were revealed in the present study.

By revealing the SMN reconstruction and significant nodes in SMA syndrome recovery, the results of this research proved that the lesioned‐hemispheric upper limb region of Brodmann area 4 (A4ul) may be a therapeutic target to facilitate motor function recovery in patients with SMA motor syndrome.

## 2 Methods

### 2.1 Patients

Forty‐two patients with motor SMA syndrome after low‐grade SMA gliomas AC between January 2020 and August 2022 at Beijing Tiantan Hospital were retrospectively reviewed. Inclusion criteria comprised the following: (1) Adult patients; (2) No history of surgical treatment or adjacent therapy; (3) Gliomas mainly located on the SMA; (4) Right‐handed patients; (5) underwent AC glioma resection. The exclusion criteria were as follows: (1) Glioma‐induced midline shifting; (2) Head motion exceeding 1 mm in translation or 1° in rotation; (3) Disturbance of consciousness and other severe postoperative complications causing an inability to complete postoperative MRI. Prior to the acquisition of data, all participants provided informed written consent. The study was approved by the Ethics Committee on Scientific Research of Beijing Tiantan Hospital. The electromyography monitoring and motor mapping procedure during the AC procedure during SMA glioma resection is described in detail in Data S1, Part 1.

### 2.2 Clinical Characteristics

Dynamic changes of motor function were observed during hospitalization. To ascertain the time required for motor function recovery, follow‐up interviews were conducted with patients up to 3 months after surgery. The interval between tumor resection and the restoration of motor function to its preoperative state was defined as the recovery time. The patients were divided into two groups based on tumor location: the left group (30 cases) and the right group (12 cases). The patients were further categorized into two groups based on the recovery time: the fast‐recovery group (within 7 days) and the slow‐recovery group. Finally, four groups were formed: the left fast‐recovery group, comprising 17 cases; the left slow‐recovery group, comprising 13 cases; the right fast‐recovery group, comprising 6 cases; and the right slow‐recovery group, comprising 6 cases.

### 2.3 MRI Acquisition

MR images were acquired using a Magnetom Prisma 3.0‐T MRI scanner (Siemens). For anatomical imaging, T1‐weighted 3D sequences were employed, with the following parameters: slice number = 192; TR (repetitive time) = 2300 msec; TE (echo time) = 2.3 msec; flip angle = 8°; FOV (field of view) = 240 × 240 mm^2^; voxel size = 1. The imaging parameters included a voxel size of 0.9 × 0.9 × 5 mm^3^, a slice number of 25, a TR of 3200 msec, an echo time (TE) of 87 msec, a flip angle of 150°, and a field of view (FOV) of 220 × 220 mm^2^. A single‐shot echo‐planar imaging (EPI) sequence was employed (axial slices = 75; TR = 6000 ms; TE = 103 ms; EPI factor = 154; spatial resolution = 2.0 × 2.0 × 2.0 mm^3^; flip angle = 75°; FOV = 230 × 230 mm^2^; number of directions = 30; b = 0/1000 s/mm^2^) to conduct DTI. Conventional CT scans were acquired on the day of the procedure to assess the presence of acute ischemia following surgery. Flair MRI was conducted within 72 h to determine the extent of resection. A DTI scan was conducted 7 days after surgery to determine the state of the fiber connections.

### 2.4 Regions of Tumor Invasion

**FIGURE 1:**
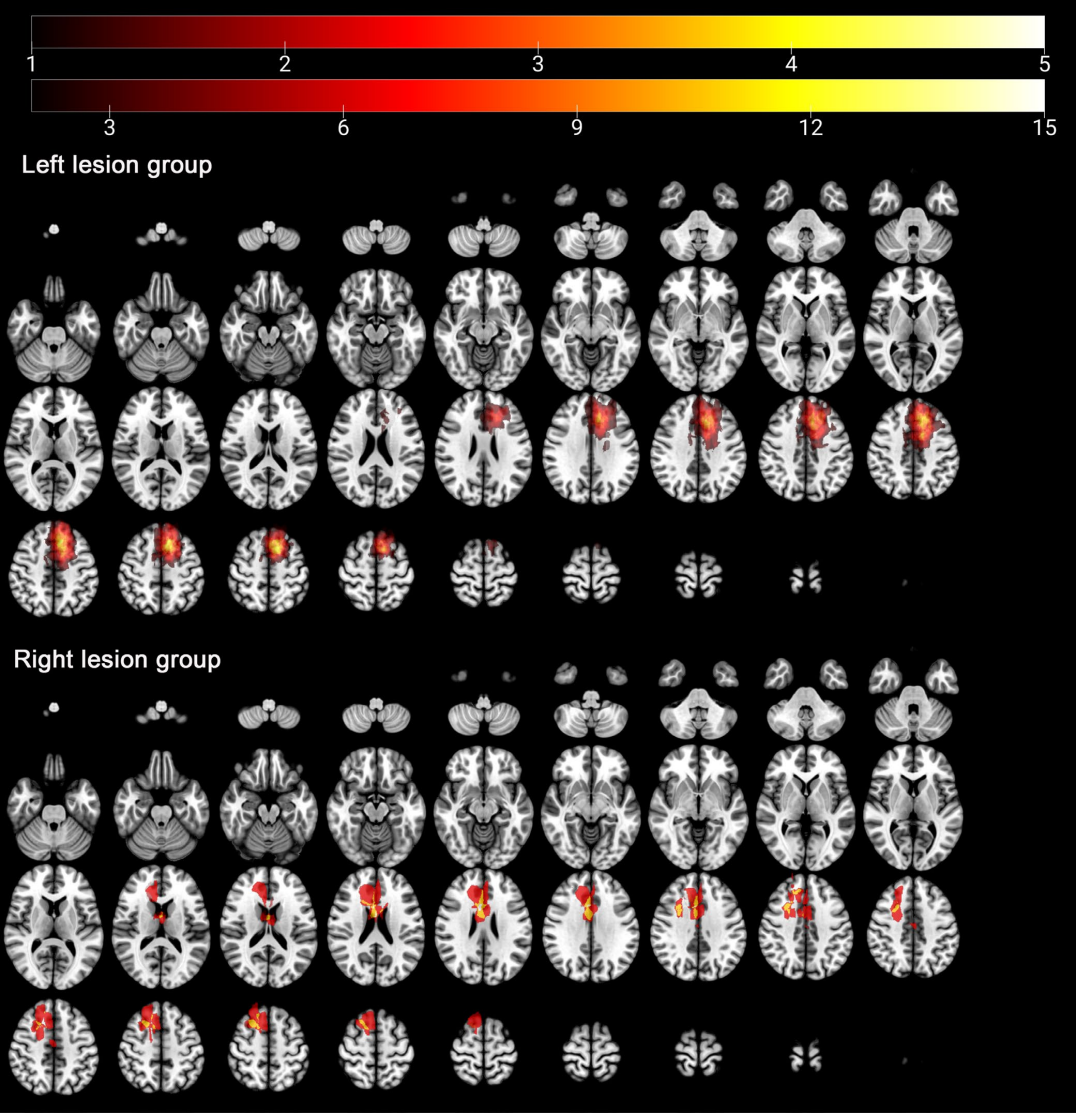
Results of tumor overlapping. The number in the color bar represented the number of gliomas with the same locations.

Tumor masks were manually delineated by one of the authors, based on the hyperintensity observed in T2‐weighted images. Another author with 15 years of experience in tumor localization verified these masks and made the final decisions. All masks were normalized to the MNI‐152 T2 template using SPM 8 software (University College London; http://www.fil.ion.ucl.ac.uk/spm/). Tumor masks were generated using a volumetric method implemented in MRIcron software (http://www.mccauslandcenter.sc.edu/mricro/mricron/). The locations and volumes of tumor invasion were calculated according to tumor masks (Figure 1).

### 2.5 DTI Preprocessing and White Matter Connectome Matrices Construction

Analyzing brain diffusion images (PANDA) software was used to preprocess DTI data (https://www.nitrc.org/projects/panda). The specific parameters employed for the maintenance of the aforementioned processes, including preprocessing, tractography, and network construction, are in accordance with the findings of previous research [15]. Skull removal (*f* = 0.25), cropping gap (mm) = 3, normalization resolution (mm) = 2, and smoothing kernel = 6 were set in our process, while other parameters remained default. Probabilistic fiber tracking was performed using the iFOD2 algorithm to construct the white matter connectome matrix of SMN [16]. Utilizing brain SMN regions from the “brant 274” atlas (www.brainnetome.org), structural connectome matrices including 30 nodes and 435 edges were generated based on fractional anisotropy (FA) for each patient (Tables S1 and S2). Based on preoperative and postoperative DTI of left lesion group patients, FA of each edge in the SMN was calculated, along with the variation of FA after surgery (FA_post‐op_—FA_pre‐op_). CST reconstruction was conducted using PANDA software with an FA threshold of 0.15. We used the mean FA of manual CST reconstruction to estimate the change of CST after surgery, which has been approved to be a good indicator of white matter integrity [17, 18].

### 2.6 Graph Theory Analysis

Graph theory analysis was used to generate global and nodal properties using the toolbox GRETNA (https://www.nitrc.org/projects/gretna) and toolbox brant (www.brainnetome.org), [19] based on the weighted FA matrices. Global properties include clustering coefficient, global efficiency, shortest path length, local efficiency, transitivity, vulnerability, fault tolerance, and small‐world properties (gamma, lambda, and sigma). Nodal properties include efficiency, local efficiency, degree centrality, and vulnerability. All Graph theory properties are explained in detail in Data S1, Part 2. The sparsity value was set to 1, considering the sparse nature of the fiber connectome matrix.

### 2.7 Shortest Distance From Surgical Region to Corticospinal Tract and A4ul

The shortest distance from the surgical region to the corticospinal tract (dCST) and variation of CST fiber numbers were measured to assess the damage of the surgical procedure to the CST. Distance from the surgical region to the midpoint between two endpoints of lesioned‐hemispheric A4ul was defined as *d*_A4ul_. The CST was reconstructed in a surgical navigation workstation (BrainLab), and the shortest distance from the surgical region to the CST (*d*_CST_) and A4ul (*d*_A4ul_) were manually measured by one of the authors, and another author with 15 years of experience in tumor locating checked these results and made final decisions. The *d*_CST_ was measured in axial, sagittal, and coronal views, and the shortest among them was included. The integrity of the CST after surgery was defined as the ratio of post‐operation CST fiber number to pre‐operation CST fiber number calculated in BrainLab. Identical regions of interest were used in every pair of pre‐operation and post‐operation data.

### 2.8 Statistical Analysis

Statistical analyses were performed using SPSS software (version 25.0, IBM Corp.) and GraphPad Prism8.0 (GraphPad Software). Clinical data comparisons between groups were conducted using Student's *t*‐test and chi‐square test, depending on the data type. Pearson's correlation analysis was used to examine associations between SMA syndrome recovery time and possible affecting factors, including age, glioma grade, chromosome 1p/19q status, tumor volume, extent of resection, *d*_CST_, *d*_A4ul_, variation of FN, and nodal topological properties variations. Due to the relatively small sample size of the right lesion group, correlation analysis was only conducted in the left lesion group.

Nodal property comparisons among the groups utilized Student's *t*‐test and post hoc analysis by Bonferroni correction (the results were significant when the original *p* value $\leq \frac{0.05}{30}$, because total of 30 nodes in the SMN), paired‐samples t‐tests were used to determine changes after surgery within each group, and results after *post hoc* analysis with Bonferroni correction (original *p* value $\leq \frac{0.05}{30}$, because total of 30 nodes in the SMN) were considered significant, results with *p* < 0.05 were considered as having trend of difference. Cox regressions were conducted to identify factors associated with SMA syndrome recovery time using SPSS software. An univariate analysis was conducted first, and factors with significant results were included in the following multivariate analysis. Cox regressions were conducted for the patients only in the left lesion group. Short recovery time was considered as a benefit for patients. Hence, to differentiate from conventional Cox regressions, the factors with hazard ratio (HR) > 1 were considered as protective factors, with HR < 1 were considered as risk factors in this study.

A general linear model was formed using significant factors in multivariate Cox analysis to predict recovery time, and the right lesion group data was used to verify the efficiency of the linear model. The prediction score equation is as follows:

$$\begin{array}{c} \text{Prediction score}=x*\mathrm{HR}x*\frac{\bar{x}+\bar{y}+\bar{z}+\cdots }{\bar{x}}\\ \;+y*\mathrm{HR}y*\frac{\bar{x}+\bar{y}+\bar{z}+\cdots }{\bar{y}}\\ \;+z*\mathrm{HR}z*\frac{\bar{x}+\bar{y}+\bar{z}+\cdots }{\bar{z}}+\ldots \end{array}$$

Causal mediation analysis [20] was conducted to identify topological properties mediating between *d*_CST_ and recovery time using SPSS and Process version 3.0 package with specified parameters (model number = 4; confidence interval = 95; number of bootstrap = 5000).

## 3 Results

No differences were observed between the fast‐recovery and slow‐recovery groups regarding gender, age, education level, ratio of histopathology, IDH mutation state, chromosome 1p/19q codeletion, extent of resection, or tumor volume (Table 1).

**TABLE 1:** Demographic and clinical characteristics of the study population.

Characteristics	Left lesion (*n* = 30)			Right lesion (*n* = 12)		
	Fast‐recovery	Slow‐recovery	*p*	Fast‐recovery	Slow‐recovery	*p*
Gender						
Male	9	7	0.9623	4	2	0.2897
Female	8	6		2	4	
Age (years)	39.4 ± 12.3	34.6 ± 7.0	0.2360	36 ± 8.1	45.5 ± 12.6	0.1867
Handness						
Left	17	13	—	6	6	—
Right	0	0		0	0	
Education (years)	13.6 ± 4.1	14.5 ± 2.3	0.5089	14.8 ± 2.6	11.8 ± 3.6	0.1608
IDH status						
Mutation	17	13	—	6	6	—
Wild‐type	0	0		0	0	
1p/19q status						
Codeletion	9	7	0.9623	1	2	0.5490
Non‐codeletion	8	6		5	4	
Glioma grade						
Grade II	15	13	0.2139	6	5	0.3409
Grade III	2	0		0	1	
Tumor volume (mL)	30.5 ± 10.5	22.5 ± 14.8	0.1081	29.8 ± 13.5	26.4 ± 12.3	0.6875
Extent of resection	0.97 ± 0.06	0.97 ± 0.05	0.8049	0.96 ± 0.06	0.93 ± 0.07	0.4534
Intra‐op seizure						
Yes	3	2	0.8691	1	2	0.5050
No	14	11		5	4	

*Note:* Student's *t*‐test: Age, Education, Tumor volume, Extent of resection. chi‐square test: Gender, Handness, IDH status, 1p/19q status, Glioma grade, Intra‐op seizure.

### 3.1 Differences in Topological Global Properties

Regarding the left hemispheric glioma (Table S3), gamma decreased in the fast‐recovery group after surgery. Postoperative comparison showed that the shortest path length, transitivity, gamma, and sigma were significantly greater in the slow‐recovery group (Table S4). Regarding the right hemispheric glioma (Table S5), the vulnerability of the fast‐recovery group decreased after surgery. Preoperative comparison showed the shortest path length and transitivity were significantly greater in the fast‐recovery group (Table S6). Topological global properties comparison has some significant results, but similar significant results from the left and right groups were not obtained.

### 3.2 Differences in Topological Nodal Properties

**FIGURE 2:**
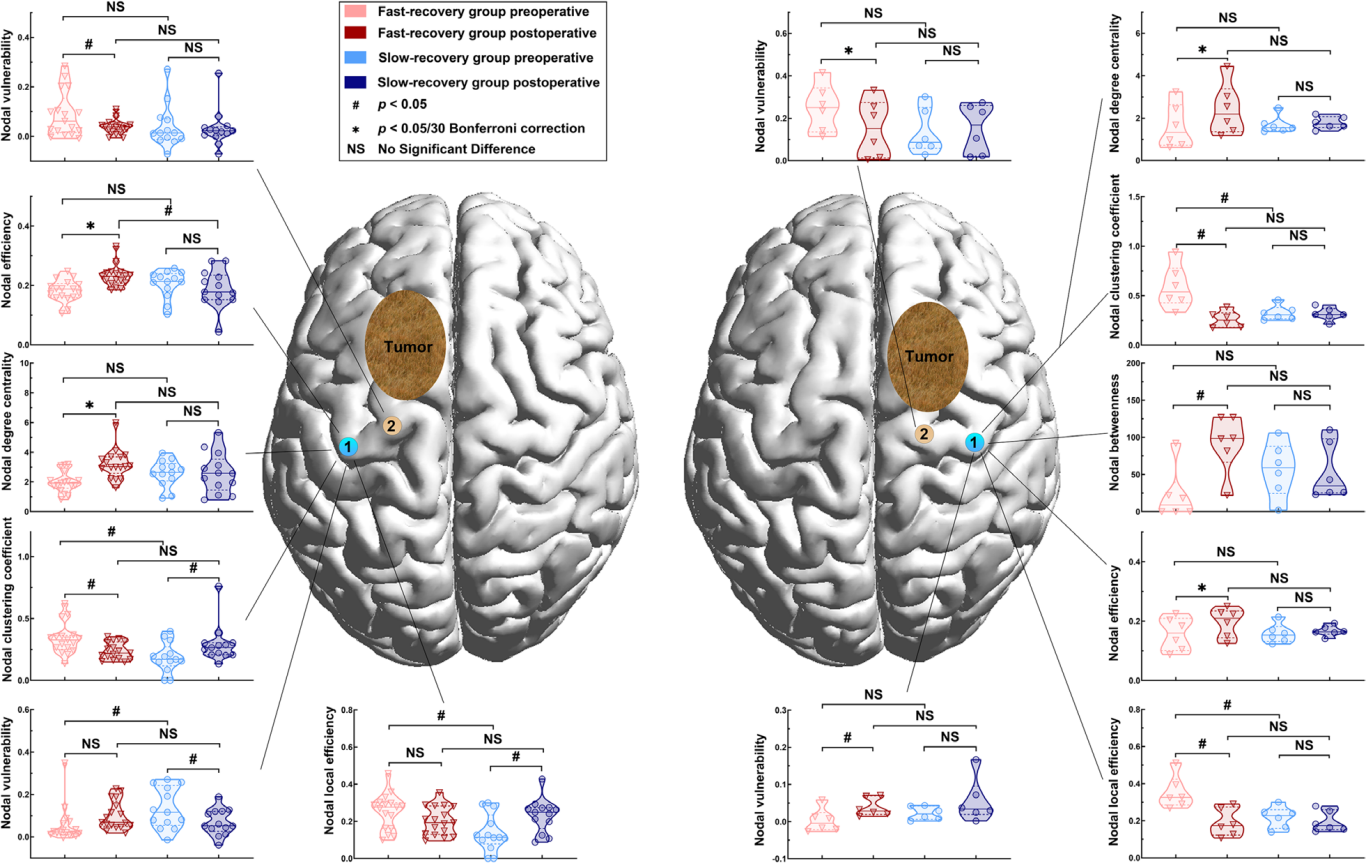
Differences of nodal properties between the fast‐recovery group and slow‐recovery group. Left: Left lesion group; Right: Right lesion group. (node no. 1 (blue) = lesional hemisphere upper limb of BA 4 (A4ul_L); node no. 2 (brown) = lesional hemisphere trunk region of BA 4 (A4t_L); node no. 3 (purple) = lesional hemisphere lower limb BA 4 (A4ll_L); node no. 4 (pink) = contralateral upper limb of BA 4 (A4ul_C); node no. 5 (green) = contralateral trunk region of BA 4 (A4t_C); node no. 5 (white) = contralateral medial area BA 6 (A6m_C).

Significant results of nodal properties comparison were found in lesioned‐hemispheric A4ul. All detailed results can be found in Data S1 (Tables S7–S30, Figure 2).

Most significant results were found in lesioned‐hemispheric A4ul. Nodal efficiency of lesioned‐hemispheric A4ul in the fast‐recovery group significantly increased after surgery (left pre‐op: 0.182 ± 0.009, left post‐op: 0.231 ± 0.008, *p* < 0.0001; right pre‐op: 0.157 ± 0.021, right post‐op: 0.195 ± 0.018, *p* = 0.0011).

Nodal degree centrality also significantly increased in the fast‐recovery group after surgery (left pre‐op: 1.985 ± 0.166; left post‐op: 3.195 ± 0.230, *p* < 0.0001; right pre‐op: 1.620 ± 0.389; right post‐op: 2.411 ± 0.452, *p* = 0.0005).

Nodal clustering coefficient has a trend of decrease in the fast‐recovery group (left pre‐op: 0.335 ± 0.029; left post‐op: 0.238 ± 0.016, *p* = 0.0047; right pre‐op: 0.589 ± 0.081; right post‐op: 0.259 ± 0.030, *p* = 0.0211), and preoperative comparison showed greater nodal clustering coefficient in the fast‐recovery group (left fast‐recovery: 0.335 ± 0.029; left slow‐recovery: 0.185 ± 0.032, *p* = 0.0025; right fast‐recovery: 0.589 ± 0.081; right slow‐recovery: 0.325 ± 0.028, *p* = 0.0185). Besides similar significant results of lesioned‐hemispheric A4ul from the left and right groups, there are still many different trends that are presented in Figure 2.

### 3.3 Correlation Between Factors and Recovery Time

**FIGURE 3:**
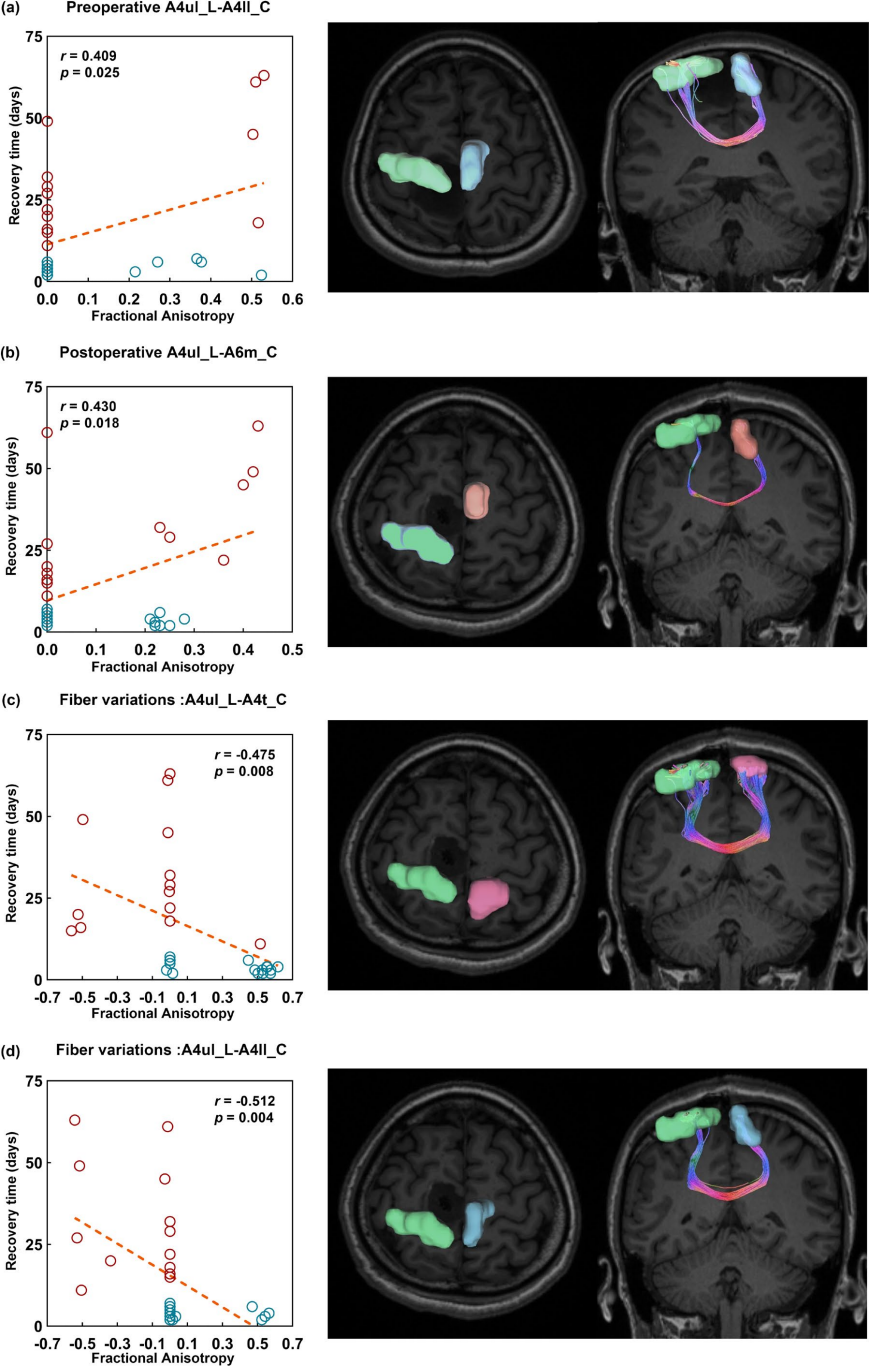
Significant correlations between SMA syndrome recovery time and preoperative fractional anisotropy (FA), postoperative FA, and FA variations in node pairs. (a) Preoperative A4ul_L‐A4ll_R; (b) Postoperative A4ul_L‐A6m_C; (c) Fiber variations: A4ul_L‐A4t_C; (d) Fiber variations: A4ul_L‐A4ll_C. Red dot: Slow‐recovery group; Blue dot: Fast‐recovery group; A4ul_L(green): Lesional hemisphere upper limb of BA 4; A4ll_C (blue): Contralateral lower limb BA 4 A6m_C(light pink): Contralateral medial area BA 6 right; A4t_C(rose red): Contralateral trunk region of BA 4.

**FIGURE 4:**
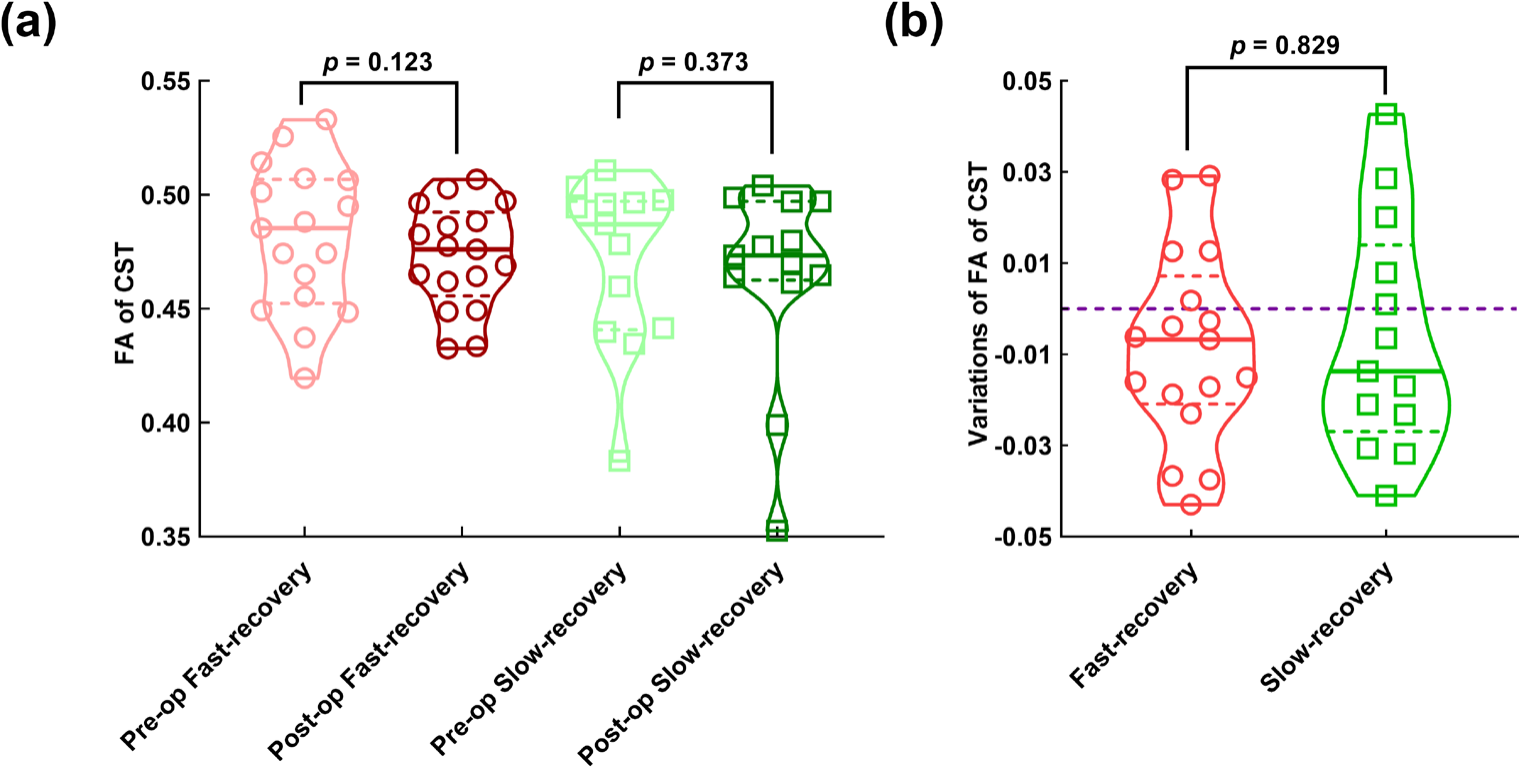
The FA variations in the Cortico‐Spinal Tract (CST) during the perioperative period. (a) Paired *t*‐test comparing preoperative and postoperative FA values in the CST for the fast‐recovery and slow‐recovery groups, respectively; (b) Unpaired t‐test analyzing FA variations in the CST between the fast‐recovery and slow‐recovery groups.

Preoperative FA between lesioned‐hemispheric A4ul and contralateral A4ll were positively correlated with recovery time (*r* = 0.409, *p* = 0.025); postoperative FA between lesioned‐hemispheric A4ul and contralateral A6m were positively correlated with recovery time (*r* = 0.430, *p* = 0.018); Variation of FA between lesioned‐hemispheric A4ul and contralateral A4ll (FA: *r* = −0.512, *p* = 0.004) and lesioned‐hemispheric A4ul and contralateral A4t (FA: *r* = −0.475, *p* = 0.008) were negatively correlated with recovery time. In the left lesion group, all significant results were lesioned‐hemispheric A4ul to contralateral SMN nodes (Figure 3). Preoperative and postoperative mean FA of CSTs have no difference in fast‐recovery and slow‐recovery groups, indicating the CSTs in both groups were preserved in general. Also, the variation of mean FA of CSTs has no difference between fast‐recovery and slow‐recovery groups (Figure 4).

**FIGURE 5:**
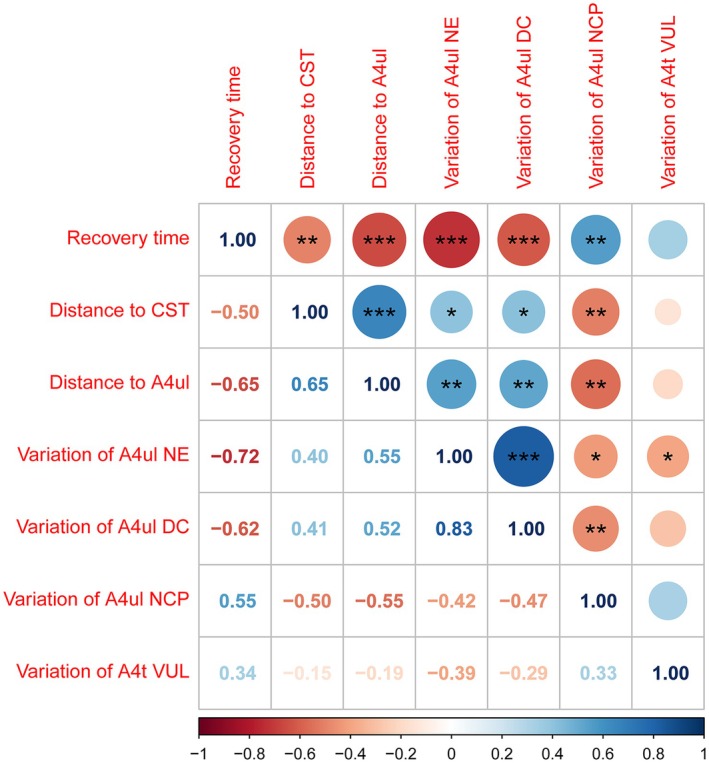
Correlation between nodal properties variations, *d*_CST_, and *d*_A4ul_ and SMA recovery time. (CST, Corticospinal tract; DC, Nodal degree centrality; NCP, Nodal clustering coefficient; NE, Nodal efficiency; VUL, Nodal vulnerability).

Correlations between recovery time, *d*_CST_, *d*_A4ul_, and significant nodal properties variations are present as a matrix (Figure 5). Results indicate the variation of A4ul nodal efficiency, the variation of lesioned‐hemispheric A4ul nodal degree centrality, *d*_CST_, and *d*_A4ul_ have significant negative correlations with recovery time (nodal efficiency: *r* = −0.720, *p* < 0.0001); (nodal degree centrality: *r* = 0.619, *p* = 0.0003); (*d*_CST_: *r* = −0.499, *p* = 0.0050; *d*_A4ul_ *r* = −0.654, *p* < 0.0001). The variation of lesioned‐hemispheric A4ul nodal clustering coefficient has a significant positive correlation with recovery time (*r* = 0.551, *p* = 0.0016). There are also correlations among distance and nodal properties variations; 4 factors (variation of lesioned‐hemispheric A4ul nodal efficiency, variation of lesioned‐hemispheric A4ul nodal degree centrality, *d*_CST_, and *d*_A4ul_) negatively related to recovery time, all have positive relationships. The variation of lesioned‐hemispheric A4ul nodal clustering coefficient has negative relationships with all 4 factors (Figure 5).

### 3.4 Cox Regression Analysis

Cox regression analysis was applied to find factors related to SMA syndrome recovery time. We included important clinical characteristics and significant factors in the analysis above. Fisher's *z*‐transformation was used in nodal properties, *d*_CST_, and *d*_A4ul_ in Cox regression analysis. Univariate analysis indicates nodal efficiency of A4ul, nodal degree centrality of A4ul, Z*d*_CST_, Z*d*_A4ul_, and histopathological grade (grade II) to be protective factors, and nodal clustering coefficient of A4ul to be a risk factor (Table 2).

**TABLE 2:** Factors for postoperative motor function recovery time through Cox regression analysis (left lesion group).

Demographic and clinical characteristics	Univariate analysis			Multivariate analysis		
	*p*	Hazard ratio	95% CI	*p*	Hazard ratio	95% CI
Age	0.237	1.022	0.986 to 1.059	—	—	—
Histopathological grade (reference: not grade 2)	0.008	14.003	1.972 to 99.405	0.932	—	—
Chromosome 1p/19q status (reference: intact)	0.577	0.800	0.379 to 1.686	—	—	—
Tumor volume	0.687	1.006	0.979 to 1.033	—	—	—
Extent of resection (standardized)	0.411	1.227	0.754 to 1.997	—	—	—
FN variation between A4ul to contralateral A4t	0.835	1.003	0.976 to 1.030	—	—	—
FN variation between A4ul to contralateral A4ll	0.875	1.005	0.950 to 1.062	—	—	—
Distance to A4ul (standardized)	< 0.001	2.634	1.630 to 4.255	0.250	—	—
Distance to corticospinal tract (standardized)	< 0.001	2.867	1.693 to 4.858	0.001	2.793	1.551 to 5.030
Variations of nodal efficiency of A4ul (standardized)	< 0.001	2.684	1.610 to 4.473	0.001	2.523	1.493 to 4.263
Variations of nodal degree centrality of A4ul (standardized)	0.001	2.305	1.411 to 3.765	0.730	—	—
Variations of nodal clustering coefficient of A4ul (standardized)	0.002	0.438	0.259 to 0.740	0.084	—	—
Variations of nodal vulnerability of A4t (standardized)	0.103	0.735	0.508 to 1.064	—	—	—

*Note:* A4ul = Brodmann area 4 upper limb region on the lesional hemisphere; A4t = Brodmann area 4 trunk region on the lesional hemisphere; Multivariate analysis was: forward, LR.

**FIGURE 6:**
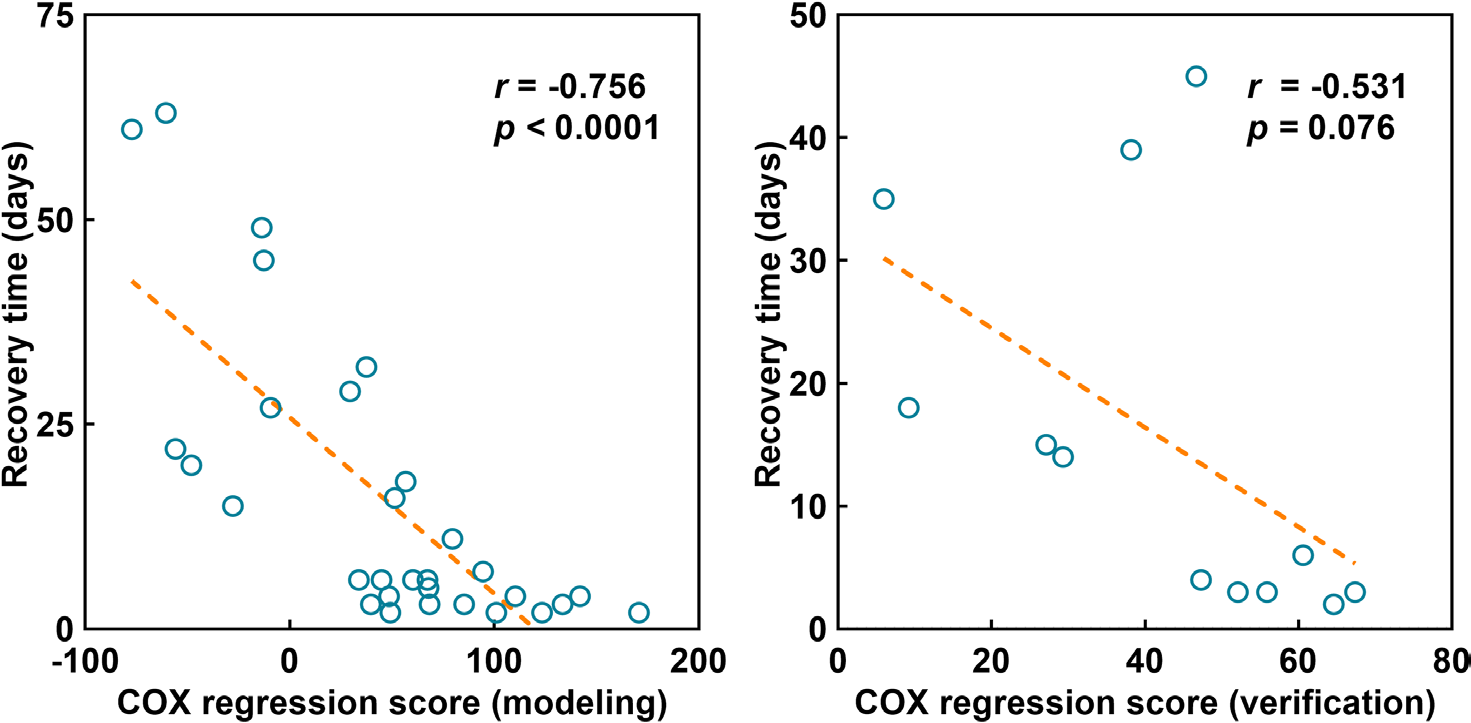
Multivariate Cox regression model to predict the recovery time of SMA; left: Modeling in left lesion group; right: Verification in right lesion group.

Results of multivariate analysis showed Z*d*_CST_ (HR = 2.793, *p* = 0.001) and nodal efficiency variation of A4ul (HR = 2.172, *p* = 0.001) are protective factors to recovery time. The significant prognostic value of *d*_CST_ and nodal efficiency variation of A4ul demonstrated above, we expect to build a robust prognostic model based on multivariate Cox regression analysis. We used these two factors adjusted by HR to build the Cox regression score to predict recovery time of SMA syndrome. A significant negative correlation between Cox regression score and recovery time was found in the left lesion group (*r* = −0.756, *p* < 0.0001), and the same trend was found in the right lesion group (*r* = −0.531, *p* = 0.076) (Figure 6). The Cox regression score has better prediction value than each single factor.

### 3.5 Causal Mediation Results

The results of causal mediation analysis (Table S31–S36) showed that variations of nodal vulnerability of lesioned‐hemispheric A4ul were a mediated factor between *d*_*CST*_ and SMA syndrome recovery time (total effect = −1.1570, direct effect [DE] = −0.8175, indirect effect [IE] = −0.3395, DE% = 70.66%, IE% = 29.34%). Meanwhile, nodal efficiency, nodal degree centrality, and postoperative vulnerability showed no mediating effect between *d*_CST_ and SMA syndrome recovery time but total indirect effect.

## 4 Discussion

The SMA plays a crucial role in the motor system, with extensive projections through the motor systems, mediated by various associations, commissural, and projection fibers [1]. This study revealed an increase in lesioned‐hemispheric A4ul nodal efficiency and long *d*_CST_ as protective factors in SMA syndrome recovery. The general linear model, formed by these two factors, demonstrated a strong correlation with recovery time. Through comparison of paired pre‐surgery and post‐surgery data, we achieved long‐term recovery outcomes prediction based on short‐term data and revealed an appropriate population for early rehabilitation intervention.

Among the superficial arcuate fibers (also known as short U‐fibers) of the SMA cortex that project to associate with other regions, the most prominent bundle forms a projection to A4ul [21, 22]. A study of microelectrode recordings from chronic tetraplegia patients revealed that the A4ul region exhibited considerable neural tuning when patients conducted face, head, arm, and leg movements. This region was found to be tuned to the entire body motor system and to contain a compositional code linking limbs together [23]. Nodal efficiency is the count backward of the shortest path length of the subnetwork that the given node participates in; it signifies the information transmission capability of the node, and nodal degree centrality is defined as the number of edges connecting to a node [24, 25]. One rs‐fMRI study of SMA region gliomas observed a high preoperative nodal efficiency of lesioned‐hemispheric A4ul, which was unexpected given the long recovery time of SMA syndrome [13]. The reason for this phenomenon is that the tumor resection caused A4ul disruption; the rest of SMN would be affected obviously in patients with high nodal efficiency compared with patients with low nodal efficiency, which indicated that the lesioned‐hemispheric A4ul was an important node for SMA syndrome occurrence. In this structural network study, the increase of nodal efficiency and nodal degree centrality of lesioned‐hemispheric A4ul was observed in fast‐recovery patients, but this increase did not occur in slow‐recovery patients. The results also indicated the importance of lesioned‐hemispheric A4ul in SMA syndrome recovery. This phenomenon suggested that SMN reconstruction during SMA syndrome recovery should focus on the lesioned‐hemispheric A4ul.

Additionally, a decrease in the number of interhemispheric fibers is observed to correlate with the long recovery time of SMA syndrome. In contrast, a variation in the number of intrahemispheric fibers does not have similar correlations. Previous rs‐fMRI studies have demonstrated that patients with brain tumors who present with preoperative motor deficits exhibit a significant reduction in interhemispheric connectivity, while intrahemispheric connectivity remains unimpaired, and interhemispheric connectivity has been observed to exhibit a negative correlation with the recovery time of SMA syndrome [12, 26], which corresponds to our findings in structural connection. Interestingly, rs‐fMRI research found the contralateral SMA region plays a major role in functional recovery [27, 28, 29], and our research of fiber number indicated the significance of fibers between the lesioned‐hemispheric A4ul and the contralateral precentral motor area (A4ll and A4t). It is conceivable that the contralateral precentral motor area serves as an intermediary node between the contralateral SMA region, given that we observed a lack of fiber number between A4ul and the contralateral SMA in most patients.

In this study, all CST of patients were intact after surgery, and *d*_*CST*_ is also a protective factor affecting the recovery time. CST is the main descending pathway in motor control [30]. SMA cortex also projects an estimated one‐tenth to one‐third of the fibers of the CST [4, 31]. Injury to the SMA results in acute hyporeflexia and akinesia; the reflex recovery after surgery occurs over days to weeks, as is expected after acute spinal cord injury [32]. The observed similarity between the manifestations of SMA syndrome and acute spinal cord injury also suggests that CST may be associated with SMA syndrome. Further investigation into the function of CST during SMA syndrome is required to elucidate the underlying mechanism of SMA syndrome. The frontal aslant tract (FAT) may be the principal fiber tract connecting the SMA and the classic Broca's area (the posterior part of the inferior frontal gyrus), and it supports the initiation functions of movement and language [33, 34]. Previous studies have demonstrated that protecting the FAT is of great significance for the postoperative aphasia score of patients with low‐grade gliomas [35, 36]. In this study, all the selected patients were those with motor SMA syndrome after surgery but without obvious language dysfunction. Therefore, we focused on the role of the CST rather than the FAT. We need to collect more aphasia SMA syndrome cases for research on the FAT.

The results of causal mediation analysis further illustrate that nodal efficiency of lesioned‐hemispheric A4ul had no mediating effect between *d*_CST_ and SMA syndrome recovery time, and *d*_CST_ had no direct effect on SMA syndrome recovery time [37]. The results demonstrated that the increase in lesioned‐hemispheric A4ul nodal efficiency was the primary protective factor of the recovery time of SMA syndrome, in comparison to *d*_CST_. The Cox regression score based on *d*_CST_ and the increase in lesioned‐hemispheric A4ul nodal efficiency has better predictive value than each single factor, indicating they both contribute to SMA syndrome recovery.

Based on the findings above, lesioned‐hemispheric A4ul plays an important role in SMA syndrome recovery, and lesioned‐hemispheric A4ul may be a therapeutic target to facilitate motor function recovery in patients with SMA syndrome.

Interestingly, rs‐fMRI researches have indicated that the lesioned‐hemispheric A4ul and cingulate cortex are important nodes for the occurrence of SMA syndrome [12, 13, 38]. In this study of structural networks, the cingulate cortex was not directly involved in the network reconstruction during SMA syndrome recovery. The structural mechanism of how the cingulate cortex is involved in SMA syndrome remains to be explored.

## 5 Limitation

The limitation of this study includes (1) small sample size of right lesion group renders some results insignificant, especially in topological properties analysis, and the therapeutic value of lesioned‐hemispheric A4ul in SMA syndrome needs further validation; (2) results of the study were not validated by prospective cohort.

## 6 Conclusion

This study revealed an increase in lesioned‐hemispheric A4ul nodal efficiency, and long *d*_CST_ are protective factors in SMA syndrome recovery. A decrease in the number of interhemispheric fibers connecting lesioned‐hemispheric A4ul to nodes on the contralateral hemisphere was correlated with the long recovery time of SMA syndrome.

## Author Contributions

Yuzhe Li, Shengyu Fang: conception and design. Shengyu Fang, Yuzhe Li, and Zhong Zhang: acquisition and analysis of data. Shengyu Fang, Yuzhe Li, Shimeng Weng, Jiahan Dong, Jiangwei Wang, Yinyan Wang, Xing Fan, Wenbin Ma: writing, review, and/or revision of the manuscript. Tao Jiang, Wenbin Ma: study supervision. The authors read and approved the final manuscript.

## Ethics Statement

This research was approved by the Ethics Committee on Scientific Research of Beijing Tiantan Hospital, Capital Medical University (approval number: KY‐2022‐206‐03).

## Consent

Written informed consent was obtained from all participants.

## Conflicts of Interest

The authors declare no conflicts of interest.

References1

Y. H.
Kim
, 
C. H.
Kim
, 
J. S.
Kim
, et al., “Risk Factor Analysis of the Development of New Neurological Deficits Following Supplementary Motor Area Resection,” Journal of Neurosurgery
119, no. 1 (2013): 7–14.23641824
10.3171/2013.3.JNS1214922

H.
Duffau
 and 
L.
Capelle
, “Preferential Brain Locations of Low‐Grade Gliomas,” Cancer
100, no. 12 (2004): 2622–2626.15197805
10.1002/cncr.202973

S. M.
Russell
 and 
P. J.
Kelly
, “Incidence and Clinical Evolution of Postoperative Deficits After Volumetric Stereotactic Resection of Glial Neoplasms Involving the Supplementary Motor Area,” Neurosurgery
52, no. 3 (2003): 506–516.12590674
10.1227/01.neu.0000047670.56996.534

J. E.
Florman
, 
H.
Duffau
, and 
A. I.
Rughani
, “Lower Motor Neuron Findings After Upper Motor Neuron Injury: Insights From Postoperative Supplementary Motor Area Syndrome,” Frontiers in Human Neuroscience
7 (2013): 85.23508473
10.3389/fnhum.2013.00085PMC36005715

A.
Krainik
, 
S.
Lehéricy
, 
H.
Duffau
, et al., “Role of the Supplementary Motor Area in Motor Deficit Following Medial Frontal Lobe Surgery,” Neurology
57, no. 5 (2001): 871–878.11552019
10.1212/wnl.57.5.8716

D.
Laplane
, 
J.
Talairach
, 
V.
Meininger
, 
J.
Bancaud
, and 
J. M.
Orgogozo
, “Clinical Consequences of Corticectomies Involving the Supplementary Motor Area in Man,” Journal of the Neurological Sciences
34, no. 3 (1977): 301–314.591992
10.1016/0022-510x(77)90148-47

K.
Rosenberg
, 
E.
Nossek
, 
R.
Liebling
, et al., “Prediction of Neurological Deficits and Recovery After Surgery in the Supplementary Motor Area: A Prospective Study in 26 Patients,” Journal of Neurosurgery
113, no. 6 (2010): 1152–1163.20635854
10.3171/2010.6.JNS10908

R.
Nakajima
, 
M.
Kinoshita
, 
T.
Yahata
, and 
M.
Nakada
, “Recovery Time From Supplementary Motor Area Syndrome: Relationship to Postoperative Day 7 Paralysis and Damage of the Cingulum,” Journal of Neurosurgery
132, no. 3 (2019): 865–874.30738403
10.3171/2018.10.JNS1823919

L.
Bulubas
, 
N.
Sardesh
, 
T.
Traut
, et al., “Motor Cortical Network Plasticity in Patients With Recurrent Brain Tumors,” Frontiers in Human Neuroscience
14 (2020): 118.32317952
10.3389/fnhum.2020.00118PMC714605010

A. R.
Potgieser
, 
B. M.
de Jong
, 
M.
Wagemakers
, 
E. W.
Hoving
, and 
R. J.
Groen
, “Insights From the Supplementary Motor Area Syndrome in Balancing Movement Initiation and Inhibition,” Frontiers in Human Neuroscience
8 (2014): 960.25506324
10.3389/fnhum.2014.00960PMC424665911

K.
Oda
, 
F.
Yamaguchi
, 
H.
Enomoto
, 
T.
Higuchi
, and 
A.
Morita
, “Prediction of Recovery From Supplementary Motor Area Syndrome After Brain Tumor Surgery: Preoperative Diffusion Tensor Tractography Analysis and Postoperative Neurological Clinical Course,” Neurosurgical Focus
44, no. 6 (2018): E3.10.3171/2017.12.FOCUS175642985276412

M.
Vassal
, 
C.
Charroud
, 
J.
Deverdun
, et al., “Recovery of Functional Connectivity of the Sensorimotor Network After Surgery for Diffuse Low‐Grade Gliomas Involving the Supplementary Motor Area,” Journal of Neurosurgery
126, no. 4 (2017): 1181–1190.27315027
10.3171/2016.4.JNS15248413

S.
Fang
, 
L.
Li
, 
S.
Weng
, et al., “Increasing Nodal Vulnerability and Nodal Efficiency Implied Recovery Time Prolonging in Patients With Supplementary Motor Area Syndrome,” Human Brain Mapping
43, no. 13 (2022): 3958–3969.35507429
10.1002/hbm.25896PMC937488614

D.
Fontaine
, 
L.
Capelle
, and 
H.
Duffau
, “Somatotopy of the Supplementary Motor Area: Evidence From Correlation of the Extent of Surgical Resection With the Clinical Patterns of Deficit,” Neurosurgery
50, no. 2 (2002): 297–303.11844264
10.1097/00006123-200202000-0001115

Z.
Cui
, 
S.
Zhong
, 
P.
Xu
, 
Y.
He
, and 
G.
Gong
, “PANDA: A Pipeline Toolbox for Analyzing Brain Diffusion Images,” Frontiers in Human Neuroscience
7, no. 42 (2013): 42, 10.3389/fnhum.2013.00042.23439846
PMC357820816

J. D.
Tournier
, 
R.
Smith
, 
D.
Raffelt
, et al., “MRtrix3: A Fast, Flexible and Open Software Framework for Medical Image Processing and Visualisation,” NeuroImage
202 (2019): 116137.31473352
10.1016/j.neuroimage.2019.11613717

T.
Wende
, 
E.
Güresir
, 
J.
Wach
, et al., “Radiomic White Matter Parameters of Functional Integrity of the Corticospinal Tract in High‐Grade Glioma,” Scientific Reports
14, no. 1 (2024): 12891.38839940
10.1038/s41598-024-63813-2PMC1115321118

F.
Zhang
, 
A.
Daducci
, 
Y.
He
, et al., “Quantitative Mapping of the Brain's Structural Connectivity Using Diffusion MRI Tractography: A Review,” NeuroImage
249 (2022): 118870.34979249
10.1016/j.neuroimage.2021.118870PMC925789119

J.
Wang
, 
X.
Wang
, 
M.
Xia
, 
X.
Liao
, 
A.
Evans
, and 
Y.
He
, “GRETNA: A Graph Theoretical Network Analysis Toolbox for Imaging Connectomics,” Frontiers in Human Neuroscience
9 (2015): 386.26175682
10.3389/fnhum.2015.00386PMC448507120

Y.
Li
, 
K.
Yoshida
, 
J. S.
Kaufman
, and 
M. B.
Mathur
, “A Brief Primer on Conducting Regression‐Based Causal Mediation Analysis,” Psychological Trauma
15, no. 6 (2023): 930–938.36701540
10.1037/tra0001421PMC1036879121

B.
Bozkurt
, 
K.
Yagmurlu
, 
E. H.
Middlebrooks
, et al., “Microsurgical and Tractographic Anatomy of the Supplementary Motor Area Complex in Humans,” World Neurosurgery
95 (2016): 99–107.27476690
10.1016/j.wneu.2016.07.07222

F.
Vergani
, 
L.
Lacerda
, 
J.
Martino
, et al., “White Matter Connections of the Supplementary Motor Area in Humans,” Journal of Neurology, Neurosurgery, and Psychiatry
85, no. 12 (2014): 1377–1385.24741063
10.1136/jnnp-2013-30749223

F. R.
Willett
, 
D. R.
Deo
, 
D. T.
Avansino
, et al., “Hand Knob Area of Premotor Cortex Represents the Whole Body in a Compositional Way,” Cell
181, no. 2 (2020): 396–409.32220308
10.1016/j.cell.2020.02.043PMC716619924

X. N.
Zuo
, 
R.
Ehmke
, 
M.
Mennes
, et al., “Network Centrality in the Human Functional Connectome,” Cerebral Cortex
22, no. 8 (2012): 1862–1875.21968567
10.1093/cercor/bhr26925

M. E.
Fox
 and 
T. Z.
King
, “Functional Connectivity in Adult Brain Tumor Patients: A Systematic Review,” Brain Connectivity
8, no. 7 (2018): 381–397.30141339
10.1089/brain.2018.062326

M. L.
Otten
, 
C. B.
Mikell
, 
B. E.
Youngerman
, et al., “Motor Deficits Correlate With Resting State Motor Network Connectivity in Patients With Brain Tumours,” Brain
135, no. Pt 4 (2012): 1017–1026, 10.1093/brain/aws041.22408270
PMC332625927

M. A.
Acioly
, 
A. M.
Cunha
, 
M.
Parise
, 
E.
Rodrigues
, and 
F.
Tovar‐Moll
, “Recruitment of Contralateral Supplementary Motor Area in Functional Recovery Following Medial Frontal Lobe Surgery: An fMRI Case Study,” Journal of Neurological Surgery Part A: Central European Neurosurgery
76, no. 6 (2015): 508–512.26291886
10.1055/s-0035-155840828

S.
Chivukula
, 
B. K.
Pikul
, 
K. L.
Black
, 
N.
Pouratian
, and 
S. Y.
Bookheimer
, “Contralateral Functional Reorganization of the Speech Supplementary Motor Area Following Neurosurgical Tumor Resection,” Brain and Language
183 (2018): 41–46.29783125
10.1016/j.bandl.2018.05.006PMC649962529

J. A.
Quirarte
, 
V. A.
Kumar
, 
H. L.
Liu
, 
K. R.
Noll
, 
J. S.
Wefel
, and 
F. F.
Lang
, “Language Supplementary Motor Area Syndrome Correlated With Dynamic Changes in Perioperative Task‐Based Functional MRI Activations: Case Report,” Journal of Neurosurgery
134, no. 6 (2020): 1738–1742.32502992
10.3171/2020.4.JNS19325030

R. N.
Lemon
, “Descending Pathways in Motor Control,” Annual Review of Neuroscience
31 (2008): 195–218.10.1146/annurev.neuro.31.060407.1255471855885331

P.
Nachev
, 
C.
Kennard
, and 
M.
Husain
, “Functional Role of the Supplementary and Pre‐Supplementary Motor Areas,” Nature Reviews. Neuroscience
9, no. 11 (2008): 856–869.18843271
10.1038/nrn247832

P. L.
Dittuno
 and 
J. F.
Ditunno, Jr.
, “Walking Index for Spinal Cord Injury (WISCI II): Scale Revision,” Spinal Cord
39, no. 12 (2001): 654–656.11781863
10.1038/sj.sc.310122333

T.
Wende
, 
A.
Hoffmann
, 
C.
Scherlach
, et al., “Preserved White Matter Integrity and Recovery After Brain Tumor Surgery: A Prospective Pilot Study on the Frontal Aslant Tract,” Brain Connectivity
13, no. 10 (2023): 589–597.37646398
10.1089/brain.2023.003334

R. G.
Briggs
, 
P. G.
Allan
, 
A.
Poologaindran
, et al., “The Frontal Aslant Tract and Supplementary Motor Area Syndrome: Moving Towards a Connectomic Initiation Axis,” Cancers (Basel)
13, no. 5 (2021): 1116.33807749
10.3390/cancers13051116PMC796136435

J. S.
Young
, 
A. J.
Gogos
, 
A. A.
Aabedi
, et al., “Resection of Supplementary Motor Area Gliomas: Revisiting Supplementary Motor Syndrome and the Role of the Frontal Aslant Tract,” Journal of Neurosurgery
136, no. 5 (2022): 1278–1284.34598138
10.3171/2021.4.JNS2118736

J. M.
Aliaga‐Arias
, 
J.
Jung
, 
J. P.
Lavrador
, et al., “Asymmetry of the Frontal Aslant Tract and Development of Supplementary Motor Area Syndrome,” Cancers (Basel)
16, no. 22 (2024): 3739.39594695
10.3390/cancers16223739PMC1159234137

A. F.
Hayes
 and 
K. J.
Preacher
, “Quantifying and Testing Indirect Effects in Simple Mediation Models When the Constituent Paths Are Nonlinear,” Multivariate Behavioral Research
45, no. 4 (2010): 627–660.26735713
10.1080/00273171.2010.49829038

C.
Kuang
, 
Y.
Zha
, 
C.
Liu
, and 
J.
Chen
, “Altered Topological Properties of Brain Structural Covariance Networks in Patients With Cervical Spondylotic Myelopathy,” Frontiers in Human Neuroscience
14 (2020): 364.33100992
10.3389/fnhum.2020.00364PMC7500316

## Supporting information


Data S1.

## Data Availability

Data will be available on reasonable request.
