# Supplementary material for: The Variation of White Matter Connectome After Surgery Revealed Factors Affecting Supplementary Syndrome Recovery Time in Low‐Grade Glioma Patients
Source: CNS Neurosci Ther. 2025 May 10;31(5):e70426. doi: 10.1111/cns.70426 (PMC12064937; doi:10.1111/cns.70426)
Supplement: Supplementary file 1 — Data S1. [file CNS-31-e70426-s001.docx]

## Supplementary Materials

## Part 1. Electromyography monitoring and Motor mapping procedure

Electromyography monitoring was used to find central sulcus before direct cortical and subcortical motor and sensory mapping. Motor and sensory mapping was performed by authors with more than 15 years of experience in functions mapping. Ojemann stimulators were used to mapping motor eloquent areas (Radionics, Burlington, Massachusetts; intensity 1–6 mA, square wave, frequency 60 Hz, and duration 1 s). Patients lay on the side and palm upwards. Bipolar stimulation current began at 1.0 mA and gradually increased (0.5 mA each time). Threshold of stimulation was determined when first positive motor reaction occurred. motor mapping on the precentral gyrus and sensory mapping on the postcentral gyrus were performed in order.

In motor mapping, stimulation induced contralateral fingers or wrist unconsciously flexed or extended, or the corner of mouth unconsciously twitching was defined as positive reactions. In sensory mapping, stimulation induced contralateral fingers, palm, wrist, or lips feeling numbness (like electrical sensation) were defined as positive reaction. All sites were stimulated three times. Twice stimulations induced positive reactions was defined as positive for the site. The positive sites were subsequently labeled by sterile markers of 5 mm diameters.

## Part 2. Graph theory properties

This part explains the graph theory properties used in this research. Graph theory properties include global properties and nodal properties. **Global properties** describe the overall structure and behavior of a brain network, these properties help in understanding the nature of the brain structural network, including its connectivity, symmetry, and traversal efficiency. Global properties mentioned in this research are explained as follow:

**・Global efficiency** measures how efficiently information can be exchanged between any two nodes in a network. It is based on the average of the inverse of the shortest path length between all pairs of nodes in the brain network.

**・Local efficiency** measures how efficiently information is exchanged between the neighbors of a node when that node is removed. It focuses on the robustness of local neighborhoods within the network.

**・Clustering coefficient** measures the degree to which nodes in a graph tend to cluster together, forming triangles. It quantifies the likelihood that two neighbors of a node are also connected to each other.

**・Shortest path length** between two nodes is the minimum number of edges that must be traversed to travel from one node to another.

**・Transitivity** is a global measure of clustering in a graph and is closely related to the clustering coefficient. It measures the likelihood that two adjacent nodes of a given node are also connected.

**・Vulnerability** refers to the susceptibility of a network to node or edge failures, and how removing certain nodes or edges can disrupt the connectivity or performance of the entire network.

**・Fault tolerance** refers to the ability of a network to continue functioning correctly even when some of its components (nodes or edges) fail or are removed.

**・The small-world properties** in graph theory refers to a characteristic of certain types of networks, where most nodes are not directly connected to each other, but the path length between any two nodes is relatively short. The normal brain structural network is a small-world network, the change of small-world property helps in understanding the traversal efficiency variation. The small-world properties include three properties: **Gamma (γ)**: the ratio of the clustering coefficient of the graph to the clustering coefficient of a random graph with the same number of nodes and edges (normalized clustering coefficient); **Lambda (λ)**: the ratio of the characteristic path length of the graph to the characteristic path length of a random graph with the same number of nodes and edges. (normalized characteristic path length); **Sigma (σ)**: the ratio of γ and λ (small-world coefficient).

**Nodal properties** describe characteristics related to individual cortex nodes rather than focusing on the entire structural network. These properties help in understanding the local behavior of individual cortex nodes and their relationships with other nodes.

**・Nodal Efficiency**: Measures how efficiently a node can communicate with the rest of the network.

**・Nodal Degree Centrality**: Quantifies the number of direct connections a node has, indicating its local importance.

**・Nodal Local Efficiency**: Evaluates the robustness of the node's local neighborhood in terms of communication efficiency when the node is removed.

**・Nodal Betweenness**: Indicates how often a node lies on the shortest paths between other nodes, showing its role as a bridge.

**・Nodal Clustering Coefficient**: Measures the tendency of a node's neighbors to also be connected, indicating local cohesion.

**・Nodal Vulnerability**: Assesses the impact of removing a node on the overall network's connectivity and functionality.

| **Table S1. Montreal Neurological Institute locations of 30 nodes in the sensorimotor network for left glioma** | | | | | | | |
| --- | --- | --- | --- | --- | --- | --- | --- |
| **Regions of interesting** | **Modified Cyto-architectonic** | **Lesional hemisphere** | | | **Healthy hemisphere** | | |
|  |  | X | Y | Z | X | Y | Z |
| A6dl_C | Dorsolateral area BA 6 | - | - | - | 20 | 4 | 64 |
| A6m_C | Medial area BA 6 | - | - | - | 7 | -4 | 60 |
| A6vl_C | Ventrolateral BA 6 | - | - | - | 34 | 8 | 54 |
| A6cdl_C | Caudal dorsolateral BA 6 | - | - | - | 33 | -7 | 57 |
| A4ul_L(C) | Area BA 4 (upper limb) | -26 | -25 | 63 | 34 | -19 | 59 |
| A4t_L(C) | Area BA 4 (trunk) | -13 | -20 | 73 | 15 | -22 | 71 |
| A4tl_L(C) | Area BA 4 (tongue and larynx) | -52 | 0 | 8 | 54 | 4 | 9 |
| A6cvl_L(C) | Caudal ventrolateral BA 6 | -49 | 5 | 30 | 51 | 7 | 30 |
| A1/2/3ll_C | Area BA 1/2/3 (lower limb) | - | - | - | 10 | -34 | 54 |
| A4ll_C | Area BA 4 (lower limb) | - | - | - | 5 | -21 | 61 |
| A1/2/3ulhf_L(C) | Area BA 1/2/3 (upper limb and face) | -50 | -16 | 43 | 50 | -14 | 44 |
| A1/2/3tonIa_L(C) | Area BA 1/2/3 (tongue and larynx) | -56 | -14 | 16 | 56 | -10 | 15 |
| A2_L(C) | Area BA 2 | -46 | -30 | 50 | 48 | -24 | 48 |
| A1/2/3tru_L(C) | Area BA 1/2/3 (trunk) | -21 | -35 | 68 | 20 | -33 | 69 |
| A24cd_ L(C) | Caudal dorsal BA 24 | -5 | 7 | 37 | 4 | 6 | 38 |
| A23c_ L(C) | Caudal area BA 23 | -7 | -23 | 41 | 6 | -20 | 40 |
| MPMtha_L(C) | Pre-motor thalamus | -18 | -13 | 3 | 12 | -14 | 1 |
| Stha_L(C) | Sensory thalamus | -18 | -23 | 4 | 18 | -22 | 3 |
| *BA = Brodmann area. L: lesioned-hemispheric; C: contralateral. | | | | | | | |

## Part 2. Supplementary tables

| **Table S2. Montreal Neurological Institute locations of 30 nodes in the sensorimotor network for right glioma** | | | | | | | |
| --- | --- | --- | --- | --- | --- | --- | --- |
| **Regions of interesting** | **Modified Cyto-architectonic** | **Lesional hemisphere** | | | **Healthy hemisphere** | | |
|  |  | X | Y | Z | X | Y | Z |
| A6dl_C | Dorsolateral area BA 6 | -18 | -1 | 65 | - | - | - |
| A6m_C | Medial area BA 6 | -6 | -5 | 68 | - | - | - |
| A6vl_C | Ventrolateral BA 6 | -32 | 4 | 55 | - | - | - |
| A6cdl_C | Caudal dorsolateral BA 6 | -32 | -9 | 58 | - | - | - |
| A4ul_L(C) | Area BA 4 (upper limb) | -26 | -25 | 63 | 34 | -19 | 59 |
| A4t_L(C) | Area BA 4 (trunk) | -13 | -20 | 73 | 15 | -22 | 71 |
| A4tl_L(C) | Area BA 4 (tongue and larynx) | -52 | 0 | 8 | 54 | 4 | 9 |
| A6cvl_L(C) | Caudal ventrolateral BA 6 | -49 | 5 | 30 | 51 | 7 | 30 |
| A1/2/3ll_C | Area BA 1/2/3 (lower limb) | -8 | -38 | 58 | - | - | - |
| A4ll_C | Area BA 4 (lower limb) | -4 | -23 | 61 | - | - | - |
| A1/2/3ulhf_L(C) | Area BA 1/2/3 (upper limb and face) | -50 | -16 | 43 | 50 | -14 | 44 |
| A1/2/3tonIa_L(C) | Area BA 1/2/3 (tongue and larynx) | -56 | -14 | 16 | 56 | -10 | 15 |
| A2_L(C) | Area BA 2 | -46 | -30 | 50 | 48 | -24 | 48 |
| A1/2/3tru_L(C) | Area BA 1/2/3 (trunk) | -21 | -35 | 68 | 20 | -33 | 69 |
| A24cd_ L(C) | Caudal dorsal BA 24 | -5 | 7 | 37 | 4 | 6 | 38 |
| A23c_ L(C) | Caudal area BA 23 | -7 | -23 | 41 | 6 | -20 | 40 |
| MPMtha_L(C) | Pre-motor thalamus | -18 | -13 | 3 | 12 | -14 | 1 |
| Stha_L(C) | Sensory thalamus | -18 | -23 | 4 | 18 | -22 | 3 |
| *BA = Brodmann area. | | | | | | | |

The p value with label ‘*’ meant the result was significant after Bonferroni correction (p < 0.05).

| **Table S3. Global properties between fast-recovery and slow-recovery groups (left** **lesion)** | | | | | | | | |
| --- | --- | --- | --- | --- | --- | --- | --- | --- |
| Global properties | Preoperative | | Postoperative | | *p* value  (Two-sample *t* test) | | *p* value  (Paired t test) | |
|  | Fast-recovery | Slow-recovery | Fast-recovery | Slow-recovery | Preoperative | Postoperative | Fast-recovery | Slow-recovery |
|  |  |  |  |  | Fast-recovery *vs.* Slow-recovery | Fast-recovery *vs.* Slow-recovery | Preoperative *vs.* Postoperative | Preoperative *vs.* Postoperative |
| Global efficiency | 0.151 ± 0.007 | 0.150 ± 0.007 | 0.162 ± 0.005 | 0.144 ± 0.008 | 0.8689 | 0.0603 | 0.0792 | 0.5677 |
| Local efficiency | 0.190 ± 0.010 | 0.193 ± 0.009 | 0.195 ± 0.009 | 0.202 ± 0.010 | 0.8568 | 0.6229 | 0.6532 | 0.4834 |
| Clustering coefficient | 0.270 ± 0.010 | 0.297 ± 0.012 | 0.277 ± 0.011 | 0.302 ± 0.015 | 0.1069 | 0.1933 | 0.6211 | 0.7892 |
| Shortest path length | 6.865 ± 0.338 | 6.880 ± 0.318 | 6.273 ± 0.178 | 7.177 ± 0.380 | 0.9762 | 0.0333 | 0.0602 | 0.5322 |
| Transitivity | 0.159 ± 0.005 | 0.159 ± 0.006 | 0.162 ± 0.004 | 0.175 ± 0.004 | 0.9686 | 0.0426 | 0.6017 | 0.0175 |
| Vulnerability | 0.175 ± 0.021 | 0.212 ± 0.021 | 0.162 ± 0.012 | 0.169 ± 0.025 | 0.2536 | 0.7827 | 0.6138 | 0.1791 |
| Fault tolerance | 1.353 ± 0.052 | 1.407 ± 0.061 | 1.392 ± 0.044 | 1.420 ± 0.064 | 0.5217 | 0.7194 | 0.4984 | 0.8039 |
| GAMMA | 3.039 ± 0.274 | 2.964 ± 0.136 | 2.390 ± 0.101 | 2.968 ± 0.266 | 0.8311 | 0.0401 | 0.0416 | 0.9878 |
| LAMBDA | 1.173 ± 0.017 | 1.131 ± 0.019 | 1.146 ± 0.018 | 1.175 ± 0.032 | 0.1233 | 0.4252 | 0.2423 | 0.2441 |
| SIGMA | 2.594 ± 0.240 | 2.622 ± 0.119 | 2.089 ± 0.089 | 2.501 ± 0.183 | 0.9265 | 0.0450 | 0.0777 | 0.5293 |

| **Table S4. Postoperative variations of Global properties between fast-recovery and slow-recovery groups (left lesion)** | | | |
| --- | --- | --- | --- |
| Global properties | Fast-recovery | Slow-recovery | *p* value  (Two-sample *t* test) |
| Global efficiency | 0.010 ± 0.005 | -0.005 ± 0.009 | 0.1325 |
| Local efficiency | 0.005 ± 0.010 | 0.009 ± 0.013 | 0.7880 |
| Clustering coefficient | 0.007 ± 0.014 | 0.005 ± 0.018 | 0.9283 |
| Shortest path length | -0.592 ± 0.284 | 0.297 ± 0.443 | 0.1006 |
| Transitivity | 0.003 ± 0.006 | 0.016 ± 0.006 | 0.1387 |
| Vulnerability | -0.014 ± 0.025 | -0.043 ± 0.029 | 0.4687 |
| Fault tolerance | 0.039 ± 0.055 | 0.013 ± 0.050 | 0.7462 |
| GAMMA | -0.648 ± 0.284 | 0.004 ± 0.273 | 0.1289 |
| LAMBDA | -0.027 ± 0.021 | 0.044 ± 0.034 | 0.0894 |
| SIGMA | -0.505 ± 0.260 | -0.121 ± 0.179 | 0.2789 |
| The *p* value with label ‘*’ meant the result was significant after Bonferroni correction (p < 0.05). | | | |

| **Table S5. Global properties between fast-recovery and slow-recovery groups (right lesion)** | | | | | | | | |
| --- | --- | --- | --- | --- | --- | --- | --- | --- |
| Global properties | Preoperative | | Postoperative | | *p* value  (Two-sample *t* test) | | *p* value  (Paired t test) | |
|  | Fast-recovery | Slow-recovery | Fast-recovery | Slow-recovery | Preoperative | Postoperative | Fast-recovery | Slow-recovery |
|  |  |  |  |  | Fast-recovery *vs.* Slow-recovery | Fast-recovery *vs.* Slow-recovery | Preoperative *vs.* Postoperative | Preoperative *vs.* Postoperative |
| Global efficiency | 0.148 ± 0.012 | 0.131 ± 0.010 | 0.154 ± 0.011 | 0.145 ± 0.007 | 0.3646 | 0.5123 | 0.5448 | 0.0567 |
| Local efficiency | 0.199 ± 0.014 | 0.154 ± 0.008 | 0.185 ± 0.011 | 0.165 ± 0.018 | 0.0336 | 0.4137 | 0.0620 | 0.5979 |
| Clustering coefficient | 0.307 ± 0.021 | 0.247 ± 0.014 | 0.282 ± 0.025 | 0.263 ± 0.023 | 0.0569 | 0.6109 | 0.1791 | 0.6464 |
| Shortest path length | 7.242 ± 0.594 | 7.876 ± 0.573 | 6.899 ± 0.585 | 7.017 ± 0.330 | 0.4995 | 0.8762 | 0.4657 | 0.0713 |
| Transitivity | 0.168 ± 0.009 | 0.136 ± 0.007 | 0.169 ± 0.009 | 0.142 ± 0.010 | 0.0314 | 0.1097 | 0.8409 | 0.6190 |
| Vulnerability | 0.288 ± 0.029 | 0.300 ± 0.055 | 0.197 ± 0.034 | 0.260 ± 0.012 | 0.8614 | 0.1361 | 0.0165 | 0.5587 |
| Fault tolerance | 1.272 ± 0.081 | 1.187 ± 0.065 | 1.300 ± 0.093 | 1.207 ± 0.077 | 0.4729 | 0.4951 | 0.7050 | 0.8623 |
| GAMMA | 2.925 ± 0.254 | 3.196 ± 0.176 | 2.776 ± 0.346 | 3.023 ± 0.272 | 0.4430 | 0.6208 | 0.6411 | 0.6866 |
| LAMBDA | 1.177 ± 0.074 | 1.151 ± 0.014 | 1.183 ± 0.078 | 1.141 ± 0.020 | 0.7550 | 0.6402 | 0.8829 | 0.6486 |
| SIGMA | 2.529 ± 0.189 | 2.781 ± 0.161 | 2.374 ± 0.253 | 2.631 ± 0.203 | 0.3740 | 0.4854 | 0.4851 | 0.6713 |

The p value with label ‘*’ meant the result was significant after Bonferroni correction (p < 0.05).

| **Table S6. Postoperative variations of Global properties between fast-recovery and slow-recovery groups (right lesion)** | | | |
| --- | --- | --- | --- |
| Global properties | Fast-recovery | Slow-recovery | *p* value  (Two-sample *t* test) |
| Global efficiency | 0.007 ± 0.010 | 0.013 ± 0.005 | 0.6086 |
| Local efficiency | -0.014 ± 0.005 | 0.011 ± 0.018 | 0.2464 |
| Clustering coefficient | -0.024 ± 0.014 | 0.016 ± 0.031 | 0.2969 |
| Shortest path length | -0.343 ± 0.397 | -0.859 ± 0.343 | 0.3903 |
| Transitivity | 0.001 ± 0.005 | 0.006 ± 0.010 | 0.7133 |
| Vulnerability | -0.091 ± 0.023 | -0.040 ± 0.058 | 0.4700 |
| Fault tolerance | 0.029 ± 0.066 | 0.020 ± 0.099 | 0.9468 |
| GAMMA | -0.148 ± 0.273 | -0.173 ± 0.369 | 0.9620 |
| LAMBDA | 0.006 ± 0.037 | -0.010 ± 0.018 | 0.7305 |
| SIGMA | -0.155 ± 0.188 | -0.150 ± 0.305 | 0.9911 |
| The *p* value with label ‘*’ meant the result was significant after Bonferroni correction (p < 0.05). | | | |

| **Table S7. Nodal efficiency between fast-recovery and slow-recovery groups (left** **lesion)** | | | | | | | | |
| --- | --- | --- | --- | --- | --- | --- | --- | --- |
| Nodal | Preoperative | | Postoperative | | *p* value (Two-sample t test) | | *p* value (Paired t test) | |
|  | Fast-recovery | Slow-recovery | Fast-recovery | Slow-recovery | Preoperative | Postoperative | Fast-recovery | Slow-recovery |
|  |  |  |  |  | Fast-recovery *vs.* Slow-recovery | Fast-recovery *vs.* Slow-recovery | Preoperative *vs.* Postoperative | Preoperative *vs.* Postoperative |
| A6dl_C | 0.186 ± 0.009 | 0.178 ± 0.007 | 0.192 ± 0.007 | 0.161 ± 0.008 | 0.5268 | 0.0096 | 0.5923 | 0.0572 |
| A6m_C | 0.200 ± 0.008 | 0.183 ± 0.010 | 0.210 ± 0.007 | 0.181 ± 0.015 | 0.2071 | 0.0766 | 0.2841 | 0.9405 |
| A6vl_C | 0.157 ± 0.006 | 0.150 ± 0.005 | 0.162 ± 0.005 | 0.148 ± 0.007 | 0.3690 | 0.1251 | 0.5460 | 0.7979 |
| A6cdl_C | 0.208 ± 0.009 | 0.215 ± 0.007 | 0.221 ± 0.007 | 0.199 ± 0.008 | 0.5742 | 0.0511 | 0.2146 | 0.0870 |
| A4ul_L | 0.182 ± 0.009 | 0.205 ± 0.012 | 0.231 ± 0.008 | 0.189 ± 0.017 | 0.1450 | 0.0322 | < 0.0001* | 0.2772 |
| A4ul_C | 0.193 ± 0.008 | 0.193 ± 0.005 | 0.208 ± 0.007 | 0.186 ± 0.006 | 0.9946 | 0.0374 | 0.0347 | 0.4102 |
| A4t_L | 0.204 ± 0.012 | 0.165 ± 0.019 | 0.213 ± 0.008 | 0.164 ± 0.017 | 0.0911 | 0.0117 | 0.4583 | 0.9563 |
| A4t_C | 0.212 ± 0.010 | 0.213 ± 0.010 | 0.237 ± 0.008 | 0.208 ± 0.012 | 0.9923 | 0.0564 | 0.0069 | 0.7561 |
| A4tl_L | 0.067 ± 0.014 | 0.055 ± 0.015 | 0.065 ± 0.012 | 0.051 ± 0.014 | 0.5701 | 0.4951 | 0.8605 | 0.8325 |
| A4tl_C | 0.108 ± 0.012 | 0.095 ± 0.015 | 0.105 ± 0.014 | 0.099 ± 0.012 | 0.5261 | 0.7606 | 0.8831 | 0.6741 |
| A6cvl_L | 0.081 ± 0.015 | 0.113 ± 0.015 | 0.095 ± 0.018 | 0.070 ± 0.019 | 0.1700 | 0.3703 | 0.5335 | 0.0556 |
| A6cvl_C | 0.149 ± 0.008 | 0.132 ± 0.018 | 0.144 ± 0.015 | 0.136 ± 0.018 | 0.3651 | 0.7493 | 0.6986 | 0.7495 |
| A1_2_3ll_C | 0.187 ± 0.011 | 0.170 ± 0.010 | 0.194 ± 0.008 | 0.167 ± 0.013 | 0.2994 | 0.0910 | 0.5081 | 0.8804 |
| A4ll_C | 0.181 ± 0.009 | 0.157 ± 0.016 | 0.185 ± 0.010 | 0.163 ± 0.019 | 0.2200 | 0.3182 | 0.6318 | 0.7327 |
| A1_2_3ulhf_L | 0.139 ± 0.009 | 0.155 ± 0.008 | 0.162 ± 0.005 | 0.145 ± 0.011 | 0.2212 | 0.1612 | 0.0443 | 0.4399 |
| A1_2_3ulhf_C | 0.161 ± 0.007 | 0.167 ± 0.007 | 0.170 ± 0.006 | 0.157 ± 0.008 | 0.5761 | 0.1806 | 0.2000 | 0.2318 |
| A1_2_3tonIa_L | 0.073 ± 0.013 | 0.065 ± 0.016 | 0.069 ± 0.014 | 0.068 ± 0.013 | 0.6775 | 0.9406 | 0.8062 | 0.8642 |
| A1_2_3tonIa_C | 0.105 ± 0.007 | 0.080 ± 0.015 | 0.095 ± 0.010 | 0.108 ± 0.010 | 0.1219 | 0.3726 | 0.4136 | 0.2310 |
| A2_L | 0.124 ± 0.014 | 0.146 ± 0.007 | 0.155 ± 0.007 | 0.136 ± 0.012 | 0.2152 | 0.1662 | 0.0383 | 0.4129 |
| A2_C | 0.159 ± 0.009 | 0.167 ± 0.007 | 0.177 ± 0.006 | 0.157 ± 0.014 | 0.5202 | 0.1852 | 0.0017 | 0.3814 |
| A1_2_3tru_L | 0.197 ± 0.012 | 0.181 ± 0.016 | 0.225 ± 0.007 | 0.181 ± 0.018 | 0.4402 | 0.0255 | 0.0320 | 0.9954 |
| A1_2_3tru_C | 0.197 ± 0.012 | 0.197 ± 0.009 | 0.214 ± 0.006 | 0.197 ± 0.010 | 0.9988 | 0.1386 | 0.1327 | 0.9869 |
| A24cd_L | 0.111 ± 0.016 | 0.102 ± 0.015 | 0.118 ± 0.011 | 0.106 ± 0.017 | 0.6828 | 0.5555 | 0.6939 | 0.8206 |
| A24cd_C | 0.130 ± 0.013 | 0.138 ± 0.006 | 0.131 ± 0.009 | 0.130 ± 0.009 | 0.6021 | 0.9936 | 0.9563 | 0.4630 |
| A23c_L | 0.119 ± 0.017 | 0.139 ± 0.020 | 0.147 ± 0.015 | 0.134 ± 0.020 | 0.4553 | 0.6065 | 0.2035 | 0.8156 |
| A23c_C | 0.169 ± 0.008 | 0.168 ± 0.010 | 0.168 ± 0.005 | 0.164 ± 0.010 | 0.9476 | 0.7455 | 0.9639 | 0.8455 |
| mPMtha_L | 0.124 ± 0.018 | 0.129 ± 0.016 | 0.143 ± 0.017 | 0.124 ± 0.016 | 0.8372 | 0.4393 | 0.4043 | 0.7598 |
| mPMtha_C | 0.160 ± 0.012 | 0.151 ± 0.009 | 0.155 ± 0.008 | 0.141 ± 0.013 | 0.6157 | 0.3777 | 0.7447 | 0.5852 |
| Stha_L | 0.109 ± 0.018 | 0.134 ± 0.013 | 0.127 ± 0.017 | 0.109 ± 0.019 | 0.3107 | 0.5145 | 0.3797 | 0.2810 |
| Stha_C | 0.149 ± 0.016 | 0.148 ± 0.010 | 0.136 ± 0.014 | 0.147 ± 0.010 | 0.9288 | 0.5505 | 0.4272 | 0.9848 |

The *p* value with label ‘*’ meant the result was significant after Bonferroni correction (p < 0.05/30).

| **Table S8. Nodal efficiency between fast-recovery and slow-recovery groups (right lesion)** | | | | | | | | |
| --- | --- | --- | --- | --- | --- | --- | --- | --- |
| Nodal | Preoperative | | Postoperative | | *p* value (Two-sample t test) | | *p* value (Paired t test) | |
|  | Fast-recovery | Slow-recovery | Fast-recovery | Slow-recovery | Preoperative | Postoperative | Fast-recovery | Slow-recovery |
|  |  |  |  |  | Fast-recovery *vs.* Slow-recovery | Fast-recovery *vs.* Slow-recovery | Preoperative *vs.* Postoperative | Preoperative *vs.* Postoperative |
| A6dl_C | 0.169 ± 0.016 | 0.163 ± 0.015 | 0.176 ± 0.017 | 0.169 ± 0.012 | 0.7973 | 0.7657 | 0.6271 | 0.6952 |
| A6m_C | 0.176 ± 0.013 | 0.167 ± 0.013 | 0.181 ± 0.017 | 0.168 ± 0.009 | 0.6600 | 0.5529 | 0.7947 | 0.9134 |
| A6vl_C | 0.145 ± 0.019 | 0.141 ± 0.009 | 0.149 ± 0.015 | 0.158 ± 0.012 | 0.8489 | 0.6659 | 0.8445 | 0.0239 |
| A6cdl_C | 0.183 ± 0.021 | 0.174 ± 0.014 | 0.190 ± 0.016 | 0.192 ± 0.014 | 0.7556 | 0.9512 | 0.7178 | 0.1433 |
| A4ul_L | 0.222 ± 0.019 | 0.187 ± 0.010 | 0.213 ± 0.016 | 0.212 ± 0.011 | 0.1667 | 0.9734 | 0.6627 | 0.1333 |
| A4ul_C | 0.157 ± 0.021 | 0.158 ± 0.012 | 0.195 ± 0.018 | 0.167 ± 0.006 | 0.9685 | 0.2072 | 0.0011* | 0.2827 |
| A4t_L | 0.164 ± 0.021 | 0.139 ± 0.027 | 0.209 ± 0.016 | 0.181 ± 0.011 | 0.5114 | 0.2154 | 0.1249 | 0.1459 |
| A4t_C | 0.238 ± 0.020 | 0.193 ± 0.010 | 0.217 ± 0.019 | 0.198 ± 0.010 | 0.0926 | 0.4394 | 0.2359 | 0.5050 |
| A4tl_L | 0.039 ± 0.024 | 0.060 ± 0.023 | 0.038 ± 0.020 | 0.081 ± 0.027 | 0.5675 | 0.2723 | 0.9658 | 0.5280 |
| A4tl_C | 0.123 ± 0.015 | 0.107 ± 0.009 | 0.117 ± 0.010 | 0.087 ± 0.016 | 0.3969 | 0.1744 | 0.4387 | 0.2256 |
| A6cvl_L | 0.169 ± 0.020 | 0.126 ± 0.026 | 0.160 ± 0.009 | 0.162 ± 0.014 | 0.2625 | 0.9370 | 0.6788 | 0.1438 |
| A6cvl_C | 0.178 ± 0.010 | 0.116 ± 0.023 | 0.125 ± 0.026 | 0.097 ± 0.020 | 0.0494 | 0.4479 | 0.1140 | 0.5610 |
| A1_2_3ll_C | 0.161 ± 0.009 | 0.122 ± 0.023 | 0.168 ± 0.009 | 0.165 ± 0.018 | 0.1884 | 0.9007 | 0.3297 | 0.2052 |
| A4ll_C | 0.192 ± 0.017 | 0.176 ± 0.018 | 0.207 ± 0.013 | 0.169 ± 0.012 | 0.5604 | 0.0836 | 0.5434 | 0.3444 |
| A1_2_3ulhf_L | 0.164 ± 0.011 | 0.153 ± 0.009 | 0.173 ± 0.011 | 0.164 ± 0.006 | 0.5180 | 0.5597 | 0.4415 | 0.1173 |
| A1_2_3ulhf_C | 0.160 ± 0.010 | 0.129 ± 0.007 | 0.155 ± 0.012 | 0.138 ± 0.005 | 0.0391 | 0.2429 | 0.5737 | 0.1126 |
| A1_2_3tonIa_L | 0.109 ± 0.007 | 0.061 ± 0.024 | 0.061 ± 0.022 | 0.077 ± 0.023 | 0.1104 | 0.6432 | 0.1034 | 0.6569 |
| A1_2_3tonIa_C | 0.104 ± 0.009 | 0.087 ± 0.009 | 0.113 ± 0.007 | 0.091 ± 0.016 | 0.2233 | 0.2746 | 0.0886 | 0.7618 |
| A2_L | 0.144 ± 0.015 | 0.091 ± 0.027 | 0.118 ± 0.009 | 0.142 ± 0.010 | 0.1443 | 0.1439 | 0.1692 | 0.1206 |
| A2_C | 0.158 ± 0.010 | 0.136 ± 0.010 | 0.156 ± 0.015 | 0.139 ± 0.007 | 0.1944 | 0.3571 | 0.8566 | 0.6773 |
| A1_2_3tru_L | 0.223 ± 0.015 | 0.165 ± 0.011 | 0.224 ± 0.016 | 0.192 ± 0.015 | 0.0172 | 0.1949 | 0.7888 | 0.1288 |
| A1_2_3tru_C | 0.202 ± 0.015 | 0.168 ± 0.011 | 0.189 ± 0.019 | 0.184 ± 0.013 | 0.1274 | 0.8363 | 0.4731 | 0.4961 |
| A24cd_L | 0.143 ± 0.012 | 0.127 ± 0.007 | 0.167 ± 0.011 | 0.135 ± 0.012 | 0.3160 | 0.0976 | 0.1689 | 0.5566 |
| A24cd_C | 0.044 ± 0.021 | 0.086 ± 0.016 | 0.133 ± 0.015 | 0.081 ± 0.023 | 0.1804 | 0.1208 | 0.0473 | 0.7848 |
| A23c_L | 0.163 ± 0.013 | 0.147 ± 0.006 | 0.162 ± 0.032 | 0.166 ± 0.013 | 0.3427 | 0.9222 | 0.9839 | 0.1352 |
| A23c_C | 0.083 ± 0.030 | 0.118 ± 0.022 | 0.116 ± 0.024 | 0.108 ± 0.021 | 0.4122 | 0.8286 | 0.4175 | 0.8143 |
| mPMtha_L | 0.095 ± 0.029 | 0.115 ± 0.034 | 0.128 ± 0.027 | 0.135 ± 0.026 | 0.6939 | 0.8545 | 0.3186 | 0.4740 |
| mPMtha_C | 0.136 ± 0.027 | 0.088 ± 0.023 | 0.142 ± 0.026 | 0.121 ± 0.023 | 0.2401 | 0.5848 | 0.8774 | 0.3751 |
| Stha_L | 0.059 ± 0.034 | 0.143 ± 0.012 | 0.156 ± 0.011 | 0.146 ± 0.028 | 0.0598 | 0.7618 | 0.0417 | 0.9501 |
| Stha_C | 0.122 ± 0.023 | 0.096 ± 0.028 | 0.095 ± 0.029 | 0.112 ± 0.021 | 0.5224 | 0.6773 | 0.3198 | 0.7543 |

The *p* value with label ‘*’ meant the result was significant after Bonferroni correction (p < 0.05/30).

| **Table S9. Nodal degree centrality between fast-recovery and slow-recovery groups (left** **lesion)** | | | | | | | | |
| --- | --- | --- | --- | --- | --- | --- | --- | --- |
| Nodal | Preoperative | | Postoperative | | *p* value (Two-sample t test) | | *p* value (Paired t test) | |
|  | Fast-recovery | Slow-recovery | Fast-recovery | Slow-recovery | Preoperative | Postoperative | Fast-recovery | Slow-recovery |
|  |  |  |  |  | Fast-recovery *vs.* Slow-recovery | Fast-recovery *vs.* Slow-recovery | Preoperative *vs.* Postoperative | Preoperative *vs.* Postoperative |
| A6dl_C | 2.122 ± 0.184 | 1.835 ± 0.186 | 1.976 ± 0.173 | 1.580 ± 0.154 | 0.3061 | 0.1219 | 0.5333 | 0.1503 |
| A6m_C | 2.721 ± 0.211 | 2.034 ± 0.229 | 2.686 ± 0.223 | 2.492 ± 0.307 | 0.0435 | 0.6176 | 0.9015 | 0.2373 |
| A6vl_C | 1.194 ± 0.072 | 0.991 ± 0.098 | 1.169 ± 0.097 | 1.152 ± 0.154 | 0.1100 | 0.9232 | 0.8105 | 0.3296 |
| A6cdl_C | 2.932 ± 0.188 | 3.156 ± 0.155 | 3.123 ± 0.191 | 2.844 ± 0.211 | 0.4026 | 0.3532 | 0.3748 | 0.1694 |
| A4ul_L | 1.985 ± 0.166 | 2.587 ± 0.241 | 3.195 ± 0.230 | 2.595 ± 0.360 | 0.0498 | 0.1687 | < 0.0001* | 0.9820 |
| A4ul_C | 2.331 ± 0.165 | 2.337 ± 0.115 | 2.453 ± 0.177 | 2.264 ± 0.113 | 0.9808 | 0.4225 | 0.4698 | 0.5964 |
| A4t_L | 2.270 ± 0.313 | 1.538 ± 0.305 | 2.328 ± 0.206 | 1.720 ± 0.231 | 0.1246 | 0.0684 | 0.8520 | 0.5593 |
| A4t_C | 2.900 ± 0.262 | 2.909 ± 0.293 | 3.503 ± 0.251 | 2.998 ± 0.347 | 0.9822 | 0.2529 | 0.0476 | 0.7567 |
| A4tl_L | 0.614 ± 0.094 | 0.416 ± 0.087 | 0.494 ± 0.051 | 0.304 ± 0.079 | 0.1566 | 0.0517 | 0.2149 | 0.3112 |
| A4tl_C | 0.737 ± 0.115 | 0.634 ± 0.116 | 0.790 ± 0.117 | 0.659 ± 0.103 | 0.5525 | 0.4417 | 0.6602 | 0.8170 |
| A6cvl_L | 0.557 ± 0.113 | 0.619 ± 0.112 | 0.650 ± 0.130 | 0.358 ± 0.120 | 0.7160 | 0.1327 | 0.6382 | 0.1077 |
| A6cvl_C | 1.167 ± 0.134 | 1.067 ± 0.198 | 1.114 ± 0.197 | 1.387 ± 0.282 | 0.6786 | 0.4354 | 0.7502 | 0.1840 |
| A1_2_3ll_C | 2.047 ± 0.203 | 1.675 ± 0.188 | 1.997 ± 0.197 | 1.799 ± 0.243 | 0.2162 | 0.5415 | 0.7929 | 0.5861 |
| A4ll_C | 1.923 ± 0.176 | 1.465 ± 0.258 | 1.844 ± 0.194 | 1.979 ± 0.276 | 0.1537 | 0.6932 | 0.6882 | 0.0609 |
| A1_2_3ulhf_L | 1.251 ± 0.057 | 1.351 ± 0.104 | 1.210 ± 0.107 | 1.378 ± 0.131 | 0.3962 | 0.3408 | 0.7615 | 0.8532 |
| A1_2_3ulhf_C | 1.560 ± 0.109 | 1.742 ± 0.112 | 1.649 ± 0.112 | 1.568 ± 0.165 | 0.2770 | 0.6904 | 0.5521 | 0.2455 |
| A1_2_3tonIa_L | 0.526 ± 0.057 | 0.492 ± 0.098 | 0.490 ± 0.062 | 0.388 ± 0.077 | 0.7583 | 0.3178 | 0.6533 | 0.3627 |
| A1_2_3tonIa_C | 0.663 ± 0.063 | 0.504 ± 0.096 | 0.554 ± 0.072 | 0.696 ± 0.076 | 0.1773 | 0.2082 | 0.1126 | 0.2688 |
| A2_L | 1.090 ± 0.168 | 1.200 ± 0.102 | 1.105 ± 0.113 | 1.102 ± 0.133 | 0.6182 | 0.9865 | 0.9254 | 0.5981 |
| A2_C | 1.533 ± 0.166 | 1.719 ± 0.124 | 1.767 ± 0.154 | 1.735 ± 0.180 | 0.4162 | 0.8950 | 0.0496 | 0.9143 |
| A1_2_3tru_L | 2.280 ± 0.271 | 1.971 ± 0.340 | 2.849 ± 0.247 | 2.386 ± 0.321 | 0.4915 | 0.2710 | 0.1004 | 0.3279 |
| A1_2_3tru_C | 2.520 ± 0.274 | 2.548 ± 0.181 | 2.820 ± 0.154 | 2.606 ± 0.229 | 0.9400 | 0.4459 | 0.3834 | 0.8108 |
| A24cd_L | 0.880 ± 0.128 | 0.595 ± 0.112 | 0.693 ± 0.074 | 0.859 ± 0.163 | 0.1291 | 0.3428 | 0.1625 | 0.2437 |
| A24cd_C | 1.011 ± 0.135 | 1.049 ± 0.124 | 0.940 ± 0.120 | 0.953 ± 0.122 | 0.8478 | 0.9413 | 0.6194 | 0.5842 |
| A23c_L | 0.978 ± 0.149 | 1.343 ± 0.190 | 1.236 ± 0.156 | 1.355 ± 0.243 | 0.1494 | 0.6818 | 0.2646 | 0.9651 |
| A23c_C | 1.718 ± 0.134 | 1.726 ± 0.175 | 1.595 ± 0.095 | 1.899 ± 0.112 | 0.9699 | 0.0546 | 0.3727 | 0.4899 |
| mPMtha_L | 0.730 ± 0.137 | 0.679 ± 0.116 | 0.911 ± 0.141 | 0.766 ± 0.110 | 0.7944 | 0.4630 | 0.3262 | 0.4411 |
| mPMtha_C | 1.265 ± 0.139 | 0.972 ± 0.150 | 0.922 ± 0.139 | 0.966 ± 0.125 | 0.1806 | 0.8274 | 0.0487 | 0.9801 |
| Stha_L | 0.569 ± 0.095 | 0.747 ± 0.119 | 0.680 ± 0.109 | 0.772 ± 0.162 | 0.2625 | 0.6403 | 0.4222 | 0.8383 |
| Stha_C | 1.121 ± 0.165 | 0.889 ± 0.125 | 0.768 ± 0.116 | 1.033 ± 0.137 | 0.3138 | 0.1633 | 0.0459 | 0.3480 |

The *p* value with label ‘*’ meant the result was significant after Bonferroni correction (p < 0.05/30).

| **Table S10. Nodal degree centrality between fast-recovery and slow-recovery groups (right lesion)** | | | | | | | | |
| --- | --- | --- | --- | --- | --- | --- | --- | --- |
| Nodal | Preoperative | | Postoperative | | *p* value (Two-sample t test) | | *p* value (Paired t test) | |
|  | Fast-recovery | Slow-recovery | Fast-recovery | Slow-recovery | Preoperative | Postoperative | Fast-recovery | Slow-recovery |
|  |  |  |  |  | Fast-recovery *vs.* Slow-recovery | Fast-recovery *vs.* Slow-recovery | Preoperative *vs.* Postoperative | Preoperative *vs.* Postoperative |
| A6dl_C | 2.045 ± 0.241 | 1.698 ± 0.283 | 1.837 ± 0.229 | 1.784 ± 0.347 | 0.4138 | 0.9100 | 0.1402 | 0.8280 |
| A6m_C | 1.728 ± 0.160 | 1.844 ± 0.274 | 1.950 ± 0.210 | 1.700 ± 0.247 | 0.7453 | 0.4965 | 0.4376 | 0.7214 |
| A6vl_C | 1.287 ± 0.280 | 1.119 ± 0.177 | 1.007 ± 0.163 | 1.298 ± 0.223 | 0.6544 | 0.3577 | 0.3855 | 0.2510 |
| A6cdl_C | 2.210 ± 0.313 | 2.194 ± 0.247 | 1.959 ± 0.278 | 2.418 ± 0.262 | 0.9725 | 0.2983 | 0.1841 | 0.2777 |
| A4ul_L | 3.185 ± 0.403 | 2.524 ± 0.167 | 3.044 ± 0.360 | 3.131 ± 0.274 | 0.1967 | 0.8653 | 0.8060 | 0.1960 |
| A4ul_C | 1.620 ± 0.389 | 1.691 ± 0.154 | 2.411 ± 0.452 | 1.778 ± 0.109 | 0.8794 | 0.2421 | 0.0005* | 0.5556 |
| A4t_L | 1.571 ± 0.382 | 1.335 ± 0.309 | 2.422 ± 0.359 | 1.825 ± 0.301 | 0.6701 | 0.2727 | 0.1360 | 0.1629 |
| A4t_C | 3.468 ± 0.413 | 2.176 ± 0.270 | 2.553 ± 0.427 | 2.172 ± 0.257 | 0.0380 | 0.5008 | 0.0496 | 0.9907 |
| A4tl_L | 0.190 ± 0.121 | 0.392 ± 0.131 | 0.288 ± 0.086 | 0.519 ± 0.232 | 0.3254 | 0.4142 | 0.2123 | 0.5882 |
| A4tl_C | 0.705 ± 0.104 | 0.824 ± 0.099 | 0.877 ± 0.126 | 0.624 ± 0.111 | 0.4682 | 0.1981 | 0.2119 | 0.1131 |
| A6cvl_L | 2.115 ± 0.545 | 1.298 ± 0.357 | 1.117 ± 0.109 | 1.638 ± 0.290 | 0.2795 | 0.1560 | 0.1208 | 0.4531 |
| A6cvl_C | 1.671 ± 0.224 | 1.142 ± 0.225 | 0.988 ± 0.403 | 0.568 ± 0.171 | 0.1593 | 0.4021 | 0.1770 | 0.0691 |
| A1_2_3ll_C | 1.294 ± 0.175 | 0.897 ± 0.279 | 1.224 ± 0.128 | 1.487 ± 0.325 | 0.2966 | 0.5071 | 0.7643 | 0.3236 |
| A4ll_C | 1.950 ± 0.350 | 2.092 ± 0.437 | 2.825 ± 0.302 | 1.586 ± 0.245 | 0.8212 | 0.0156 | 0.2394 | 0.1297 |
| A1_2_3ulhf_L | 1.340 ± 0.133 | 1.492 ± 0.173 | 1.645 ± 0.112 | 1.519 ± 0.094 | 0.5398 | 0.4476 | 0.0820 | 0.8723 |
| A1_2_3ulhf_C | 1.631 ± 0.137 | 1.308 ± 0.120 | 1.690 ± 0.202 | 1.329 ± 0.098 | 0.1358 | 0.1738 | 0.5270 | 0.8017 |
| A1_2_3tonIa_L | 0.403 ± 0.065 | 0.489 ± 0.182 | 0.370 ± 0.074 | 0.341 ± 0.109 | 0.6930 | 0.8434 | 0.2019 | 0.5632 |
| A1_2_3tonIa_C | 0.533 ± 0.110 | 0.533 ± 0.113 | 0.777 ± 0.065 | 0.739 ± 0.111 | 0.9969 | 0.7929 | 0.0181 | 0.2143 |
| A2_L | 0.959 ± 0.234 | 0.635 ± 0.219 | 0.481 ± 0.120 | 1.025 ± 0.215 | 0.3781 | 0.0712 | 0.1998 | 0.2234 |
| A2_C | 1.723 ± 0.186 | 1.353 ± 0.211 | 1.411 ± 0.275 | 1.364 ± 0.153 | 0.2581 | 0.8935 | 0.2376 | 0.9060 |
| A1_2_3tru_L | 3.048 ± 0.231 | 1.807 ± 0.257 | 3.439 ± 0.434 | 2.286 ± 0.400 | 0.0083 | 0.1048 | 0.3724 | 0.3413 |
| A1_2_3tru_C | 2.699 ± 0.220 | 2.016 ± 0.251 | 2.359 ± 0.336 | 2.292 ± 0.341 | 0.0913 | 0.9013 | 0.3503 | 0.6409 |
| A24cd_L | 1.206 ± 0.148 | 0.994 ± 0.061 | 1.497 ± 0.136 | 1.068 ± 0.226 | 0.2523 | 0.1685 | 0.2837 | 0.7822 |
| A24cd_C | 0.359 ± 0.084 | 0.464 ± 0.103 | 1.024 ± 0.259 | 0.632 ± 0.170 | 0.4876 | 0.2746 | 0.0731 | 0.1589 |
| A23c_L | 1.566 ± 0.154 | 1.425 ± 0.060 | 2.026 ± 0.448 | 1.887 ± 0.301 | 0.4544 | 0.8197 | 0.3713 | 0.1747 |
| A23c_C | 0.706 ± 0.159 | 0.914 ± 0.196 | 0.798 ± 0.437 | 0.824 ± 0.143 | 0.4710 | 0.9605 | 0.8609 | 0.7674 |
| mPMtha_L | 0.484 ± 0.155 | 0.960 ± 0.291 | 0.600 ± 0.244 | 0.781 ± 0.218 | 0.2163 | 0.6262 | 0.7153 | 0.3981 |
| mPMtha_C | 0.878 ± 0.173 | 0.590 ± 0.133 | 1.026 ± 0.155 | 0.808 ± 0.197 | 0.2573 | 0.4470 | 0.2135 | 0.3185 |
| Stha_L | 0.371 ± 0.220 | 0.997 ± 0.182 | 0.759 ± 0.130 | 1.267 ± 0.270 | 0.0732 | 0.1529 | 0.1055 | 0.4713 |
| Stha_C | 0.658 ± 0.086 | 0.738 ± 0.182 | 0.662 ± 0.171 | 0.657 ± 0.148 | 0.7231 | 0.9851 | 0.9815 | 0.8060 |

The *p* value with label ‘*’ meant the result was significant after Bonferroni correction (p < 0.05/30).

| **Table S11. Nodal local efficiency between fast-recovery and slow-recovery groups (left** **lesion)** | | | | | | | | |
| --- | --- | --- | --- | --- | --- | --- | --- | --- |
| Nodal | Preoperative | | Postoperative | | *p* value (Two-sample t test) | | *p* value (Paired t test) | |
|  | Fast-recovery | Slow-recovery | Fast-recovery | Slow-recovery | Preoperative | Postoperative | Fast-recovery | Slow-recovery |
|  |  |  |  |  | Fast-recovery *vs.* Slow-recovery | Fast-recovery *vs.* Slow-recovery | Preoperative *vs.* Postoperative | Preoperative *vs.* Postoperative |
| A6dl_C | 0.215 ± 0.022 | 0.243 ± 0.033 | 0.265 ± 0.024 | 0.328 ± 0.023 | 0.4839 | 0.0811 | 0.0931 | 0.0812 |
| A6m_C | 0.200 ± 0.020 | 0.204 ± 0.026 | 0.198 ± 0.020 | 0.227 ± 0.025 | 0.9040 | 0.3755 | 0.9381 | 0.4923 |
| A6vl_C | 0.362 ± 0.011 | 0.385 ± 0.012 | 0.349 ± 0.025 | 0.333 ± 0.038 | 0.1925 | 0.7246 | 0.6460 | 0.2106 |
| A6cdl_C | 0.217 ± 0.017 | 0.205 ± 0.014 | 0.235 ± 0.012 | 0.249 ± 0.014 | 0.6228 | 0.4799 | 0.1924 | 0.0392 |
| A4ul_L | 0.253 ± 0.022 | 0.139 ± 0.027 | 0.202 ± 0.018 | 0.232 ± 0.024 | 0.0036 | 0.3202 | 0.0886 | 0.0178 |
| A4ul_C | 0.265 ± 0.019 | 0.307 ± 0.011 | 0.276 ± 0.013 | 0.286 ± 0.017 | 0.0955 | 0.6679 | 0.6218 | 0.3721 |
| A4t_L | 0.201 ± 0.031 | 0.147 ± 0.047 | 0.264 ± 0.035 | 0.252 ± 0.041 | 0.3453 | 0.8362 | 0.1856 | 0.1420 |
| A4t_C | 0.221 ± 0.020 | 0.216 ± 0.016 | 0.231 ± 0.015 | 0.205 ± 0.020 | 0.8556 | 0.3073 | 0.6940 | 0.6920 |
| A4tl_L | 0.071 ± 0.033 | 0.078 ± 0.041 | 0.069 ± 0.037 | 0.075 ± 0.039 | 0.8942 | 0.9166 | 0.9772 | 0.9556 |
| A4tl_C | 0.041 ± 0.020 | 0.074 ± 0.035 | 0.058 ± 0.022 | 0.095 ± 0.041 | 0.4170 | 0.4259 | 0.5738 | 0.6304 |
| A6cvl_L | 0.064 ± 0.034 | 0.080 ± 0.038 | 0.130 ± 0.042 | 0.055 ± 0.036 | 0.7644 | 0.2221 | 0.2987 | 0.6668 |
| A6cvl_C | 0.251 ± 0.040 | 0.213 ± 0.042 | 0.175 ± 0.044 | 0.157 ± 0.037 | 0.5406 | 0.7774 | 0.2222 | 0.1160 |
| A1_2_3ll_C | 0.212 ± 0.024 | 0.238 ± 0.032 | 0.227 ± 0.024 | 0.198 ± 0.035 | 0.5136 | 0.5080 | 0.6507 | 0.4613 |
| A4ll_C | 0.247 ± 0.027 | 0.240 ± 0.044 | 0.288 ± 0.023 | 0.230 ± 0.031 | 0.8962 | 0.1544 | 0.1852 | 0.8641 |
| A1_2_3ulhf_L | 0.176 ± 0.033 | 0.183 ± 0.034 | 0.218 ± 0.040 | 0.185 ± 0.034 | 0.8759 | 0.5599 | 0.3614 | 0.9743 |
| A1_2_3ulhf_C | 0.249 ± 0.028 | 0.277 ± 0.022 | 0.273 ± 0.030 | 0.290 ± 0.032 | 0.4763 | 0.7055 | 0.4885 | 0.6715 |
| A1_2_3tonIa_L | 0.117 ± 0.044 | 0.105 ± 0.043 | 0.070 ± 0.037 | 0.049 ± 0.032 | 0.8649 | 0.6989 | 0.2800 | 0.1334 |
| A1_2_3tonIa_C | 0.104 ± 0.039 | 0.094 ± 0.042 | 0.100 ± 0.037 | 0.126 ± 0.042 | 0.8694 | 0.6531 | 0.9126 | 0.6227 |
| A2_L | 0.164 ± 0.036 | 0.237 ± 0.040 | 0.210 ± 0.038 | 0.247 ± 0.047 | 0.2061 | 0.5620 | 0.3712 | 0.8503 |
| A2_C | 0.273 ± 0.024 | 0.299 ± 0.019 | 0.291 ± 0.024 | 0.254 ± 0.027 | 0.4514 | 0.3337 | 0.6723 | 0.1584 |
| A1_2_3tru_L | 0.201 ± 0.028 | 0.216 ± 0.034 | 0.226 ± 0.017 | 0.239 ± 0.021 | 0.7420 | 0.6330 | 0.5068 | 0.5291 |
| A1_2_3tru_C | 0.209 ± 0.025 | 0.243 ± 0.021 | 0.249 ± 0.013 | 0.234 ± 0.017 | 0.3330 | 0.4781 | 0.1965 | 0.7779 |
| A24cd_L | 0.139 ± 0.035 | 0.046 ± 0.031 | 0.076 ± 0.031 | 0.123 ± 0.044 | 0.0723 | 0.3909 | 0.2400 | 0.2053 |
| A24cd_C | 0.215 ± 0.040 | 0.276 ± 0.038 | 0.206 ± 0.040 | 0.262 ± 0.050 | 0.3027 | 0.4015 | 0.8836 | 0.8083 |
| A23c_L | 0.112 ± 0.032 | 0.088 ± 0.030 | 0.116 ± 0.028 | 0.118 ± 0.034 | 0.5964 | 0.9596 | 0.9340 | 0.5713 |
| A23c_C | 0.231 ± 0.026 | 0.264 ± 0.028 | 0.242 ± 0.026 | 0.206 ± 0.031 | 0.4095 | 0.4035 | 0.8091 | 0.1168 |
| mPMtha_L | 0.075 ± 0.034 | 0.148 ± 0.047 | 0.157 ± 0.043 | 0.222 ± 0.050 | 0.2216 | 0.3418 | 0.1488 | 0.2317 |
| mPMtha_C | 0.219 ± 0.039 | 0.123 ± 0.039 | 0.157 ± 0.042 | 0.181 ± 0.049 | 0.1091 | 0.7306 | 0.3052 | 0.3466 |
| Stha_L | 0.160 ± 0.047 | 0.210 ± 0.051 | 0.149 ± 0.047 | 0.158 ± 0.044 | 0.4932 | 0.9033 | 0.8726 | 0.1347 |
| Stha_C | 0.240 ± 0.044 | 0.201 ± 0.045 | 0.144 ± 0.042 | 0.245 ± 0.044 | 0.5476 | 0.1239 | 0.1286 | 0.4365 |

The *p* value with label ‘*’ meant the result was significant after Bonferroni correction (p < 0.05/30).

| **Table S12. Nodal local efficiency between fast-recovery and slow-recovery groups (right lesion)** | | | | | | | | |
| --- | --- | --- | --- | --- | --- | --- | --- | --- |
| Nodal | Preoperative | | Postoperative | | *p* value (Two-sample t test) | | *p* value (Paired t test) | |
|  | Fast-recovery | Slow-recovery | Fast-recovery | Slow-recovery | Preoperative | Postoperative | Fast-recovery | Slow-recovery |
|  |  |  |  |  | Fast-recovery *vs.* Slow-recovery | Fast-recovery *vs.* Slow-recovery | Preoperative *vs.* Postoperative | Preoperative *vs.* Postoperative |
| A6dl_C | 0.175 ± 0.028 | 0.240 ± 0.042 | 0.166 ± 0.023 | 0.133 ± 0.030 | 0.2649 | 0.4517 | 0.8016 | 0.1629 |
| A6m_C | 0.176 ± 0.020 | 0.198 ± 0.041 | 0.188 ± 0.029 | 0.161 ± 0.027 | 0.6702 | 0.5516 | 0.5543 | 0.4157 |
| A6vl_C | 0.380 ± 0.033 | 0.254 ± 0.054 | 0.327 ± 0.036 | 0.252 ± 0.059 | 0.0981 | 0.3448 | 0.2599 | 0.9688 |
| A6cdl_C | 0.305 ± 0.050 | 0.211 ± 0.029 | 0.236 ± 0.033 | 0.191 ± 0.038 | 0.1655 | 0.4374 | 0.1036 | 0.5970 |
| A4ul_L | 0.276 ± 0.021 | 0.177 ± 0.030 | 0.258 ± 0.024 | 0.172 ± 0.024 | 0.0348 | 0.0460 | 0.5042 | 0.8742 |
| A4ul_C | 0.353 ± 0.033 | 0.218 ± 0.022 | 0.190 ± 0.028 | 0.196 ± 0.021 | 0.0112 | 0.8912 | 0.0337 | 0.5509 |
| A4t_L | 0.360 ± 0.049 | 0.145 ± 0.034 | 0.258 ± 0.040 | 0.187 ± 0.060 | 0.0078 | 0.3878 | 0.0976 | 0.5421 |
| A4t_C | 0.130 ± 0.020 | 0.136 ± 0.045 | 0.204 ± 0.061 | 0.177 ± 0.023 | 0.9194 | 0.7162 | 0.1651 | 0.5676 |
| A4tl_L | 0.000 ± 0.000 | 0.000 ± 0.000 | 0.000 ± 0.000 | 0.098 ± 0.069 | 1.0000 | 0.2250 | 1.0000 | 0.2525 |
| A4tl_C | 0.088 ± 0.058 | 0.045 ± 0.026 | 0.110 ± 0.044 | 0.116 ± 0.067 | 0.5494 | 0.9434 | 0.6544 | 0.1752 |
| A6cvl_L | 0.148 ± 0.044 | 0.107 ± 0.050 | 0.228 ± 0.081 | 0.164 ± 0.054 | 0.5883 | 0.5596 | 0.2951 | 0.3101 |
| A6cvl_C | 0.280 ± 0.045 | 0.125 ± 0.047 | 0.090 ± 0.061 | 0.116 ± 0.068 | 0.0560 | 0.7959 | 0.0253 | 0.8424 |
| A1_2_3ll_C | 0.154 ± 0.035 | 0.111 ± 0.055 | 0.339 ± 0.038 | 0.162 ± 0.056 | 0.5623 | 0.0375 | 0.0058 | 0.6464 |
| A4ll_C | 0.316 ± 0.047 | 0.154 ± 0.039 | 0.256 ± 0.021 | 0.188 ± 0.052 | 0.0364 | 0.2901 | 0.3622 | 0.4549 |
| A1_2_3ulhf_L | 0.112 ± 0.026 | 0.121 ± 0.024 | 0.145 ± 0.034 | 0.156 ± 0.046 | 0.8221 | 0.8566 | 0.4539 | 0.4363 |
| A1_2_3ulhf_C | 0.349 ± 0.021 | 0.233 ± 0.034 | 0.239 ± 0.043 | 0.273 ± 0.039 | 0.0247 | 0.5994 | 0.0264 | 0.2541 |
| A1_2_3tonIa_L | 0.000 ± 0.000 | 0.041 ± 0.024 | 0.000 ± 0.000 | 0.076 ± 0.069 | 0.1457 | 0.3409 | - | 0.7044 |
| A1_2_3tonIa_C | 0.056 ± 0.051 | 0.054 ± 0.049 | 0.275 ± 0.068 | 0.140 ± 0.065 | 0.9844 | 0.2163 | 0.0572 | 0.1486 |
| A2_L | 0.120 ± 0.069 | 0.133 ± 0.060 | 0.064 ± 0.058 | 0.118 ± 0.052 | 0.9017 | 0.5398 | 0.6403 | 0.8745 |
| A2_C | 0.321 ± 0.026 | 0.261 ± 0.026 | 0.310 ± 0.031 | 0.255 ± 0.043 | 0.1640 | 0.3595 | 0.8166 | 0.8897 |
| A1_2_3tru_L | 0.202 ± 0.024 | 0.186 ± 0.041 | 0.203 ± 0.036 | 0.232 ± 0.019 | 0.7705 | 0.5367 | 0.9651 | 0.3986 |
| A1_2_3tru_C | 0.277 ± 0.022 | 0.193 ± 0.030 | 0.210 ± 0.043 | 0.217 ± 0.049 | 0.0635 | 0.9203 | 0.0658 | 0.6311 |
| A24cd_L | 0.191 ± 0.030 | 0.256 ± 0.058 | 0.246 ± 0.055 | 0.226 ± 0.067 | 0.3904 | 0.8390 | 0.5133 | 0.7146 |
| A24cd_C | 0.066 ± 0.060 | 0.133 ± 0.077 | 0.109 ± 0.046 | 0.101 ± 0.059 | 0.5472 | 0.9200 | 0.6807 | 0.2208 |
| A23c_L | 0.248 ± 0.027 | 0.198 ± 0.044 | 0.232 ± 0.045 | 0.159 ± 0.035 | 0.3970 | 0.2650 | 0.7856 | 0.6491 |
| A23c_C | 0.073 ± 0.051 | 0.115 ± 0.045 | 0.040 ± 0.036 | 0.219 ± 0.072 | 0.5924 | 0.0709 | 0.6816 | 0.1933 |
| mPMtha_L | 0.212 ± 0.089 | 0.155 ± 0.058 | 0.025 ± 0.023 | 0.048 ± 0.028 | 0.6331 | 0.5826 | 0.1549 | 0.1198 |
| mPMtha_C | 0.194 ± 0.064 | 0.138 ± 0.080 | 0.152 ± 0.061 | 0.090 ± 0.044 | 0.6345 | 0.4614 | 0.6062 | 0.5937 |
| Stha_L | 0.114 ± 0.066 | 0.144 ± 0.069 | 0.148 ± 0.085 | 0.268 ± 0.059 | 0.7845 | 0.3120 | 0.7511 | 0.3226 |
| Stha_C | 0.300 ± 0.090 | 0.144 ± 0.067 | 0.305 ± 0.092 | 0.066 ± 0.045 | 0.2330 | 0.0584 | 0.6869 | 0.5013 |

The *p* value with label ‘*’ meant the result was significant after Bonferroni correction (p < 0.05/30).

The *p* value with label ‘*’ meant the result was significant after Bonferroni correction (p < 0.05/30).

| **Table S13. Nodal betweenness between fast-recovery and slow-recovery groups (left** **lesion)** | | | | | | | | | | | | |
| --- | --- | --- | --- | --- | --- | --- | --- | --- | --- | --- | --- | --- |
| Nodal | | Preoperative | | | | Postoperative | | *p* value (Two-sample t test) | | | *p* value (Paired t test) | |
|  |  | Fast-recovery | | Slow-recovery | | Fast-recovery | Slow-recovery | Preoperative | | Postoperative | Fast-recovery | Slow-recovery |
|  |  |  |  |  |  |  |  | Fast-recovery *vs.* Slow-recovery | | Fast-recovery *vs.* Slow-recovery | Preoperative *vs.* Postoperative | Preoperative *vs.* Postoperative |
| A6dl_C | 36.176 ± 9.493 | | 33.923 ± 13.395 | | 20.471 ± 6.948 | | 9.538 ± 6.747 | | 0.8926 | 0.2950 | 0.0804 | 0.1054 |
| A6m_C | 56.294 ± 14.296 | | 37.077 ± 9.413 | | 44.294 ± 5.359 | | 37.385 ± 9.054 | | 0.3191 | 0.5104 | 0.4409 | 0.9808 |
| A6vl_C | 0.882 ± 0.423 | | 0.154 ± 0.148 | | 1.412 ± 1.022 | | 4.615 ± 4.120 | | 0.1710 | 0.4208 | 0.6644 | 0.3205 |
| A6cdl_C | 53.941 ± 11.962 | | 79.077 ± 14.230 | | 58.412 ± 10.774 | | 45.615 ± 6.522 | | 0.1998 | 0.3703 | 0.7293 | 0.0254 |
| A4ul_L | 49.294 ± 12.580 | | 92.000 ± 13.246 | | 70.118 ± 11.328 | | 70.154 ± 13.228 | | 0.0337 | 0.9984 | 0.2777 | 0.2587 |
| A4ul_C | 39.588 ± 8.573 | | 19.692 ± 3.685 | | 35.353 ± 5.310 | | 27.615 ± 6.242 | | 0.0730 | 0.3677 | 0.6650 | 0.3376 |
| A4t_L | 57.000 ± 13.651 | | 27.154 ± 9.403 | | 26.059 ± 5.631 | | 21.308 ± 10.202 | | 0.1134 | 0.6797 | 0.0565 | 0.5467 |
| A4t_C | 68.529 ± 13.977 | | 59.692 ± 12.946 | | 72.706 ± 12.474 | | 66.923 ± 11.364 | | 0.6663 | 0.7503 | 0.8485 | 0.6798 |
| A4tl_L | 4.235 ± 2.188 | | 2.000 ± 1.922 | | 1.824 ± 1.590 | | 1.923 ± 1.848 | | 0.4796 | 0.9687 | 0.4261 | 0.9792 |
| A4tl_C | 8.647 ± 2.888 | | 6.231 ± 2.687 | | 8.529 ± 2.848 | | 6.615 ± 2.960 | | 0.5692 | 0.6601 | 0.9709 | 0.9371 |
| A6cvl_L | 3.000 ± 2.910 | | 15.000 ± 9.293 | | 4.176 ± 2.826 | | 0.077 ± 0.074 | | 0.1975 | 0.2307 | 0.7910 | 0.1492 |
| A6cvl_C | 8.941 ± 3.676 | | 7.615 ± 3.531 | | 15.294 ± 4.668 | | 14.000 ± 6.359 | | 0.8078 | 0.8723 | 0.2111 | 0.3765 |
| A1_2_3ll_C | 30.647 ± 6.221 | | 34.692 ± 13.249 | | 26.941 ± 6.411 | | 39.385 ± 14.556 | | 0.7759 | 0.4200 | 0.5777 | 0.7976 |
| A4ll_C | 20.588 ± 8.643 | | 16.769 ± 6.189 | | 11.765 ± 3.829 | | 28.077 ± 10.516 | | 0.7458 | 0.1330 | 0.3708 | 0.3788 |
| A1_2_3ulhf_L | 29.941 ± 7.588 | | 36.462 ± 7.816 | | 24.118 ± 7.267 | | 26.692 ± 6.910 | | 0.5730 | 0.8105 | 0.6056 | 0.3365 |
| A1_2_3ulhf_C | 24.765 ± 6.227 | | 21.846 ± 5.785 | | 17.765 ± 5.549 | | 14.923 ± 7.445 | | 0.7492 | 0.7649 | 0.3891 | 0.3281 |
| A1_2_3tonIa_L | 7.765 ± 3.685 | | 2.154 ± 2.069 | | 6.176 ± 2.704 | | 4.308 ± 2.467 | | 0.2472 | 0.6357 | 0.7058 | 0.5581 |
| A1_2_3tonIa_C | 6.235 ± 2.485 | | 6.231 ± 4.059 | | 0.235 ± 0.132 | | 5.385 ± 2.612 | | 0.9992 | 0.0384 | 0.0288 | 0.8504 |
| A2_L | 22.412 ± 6.950 | | 14.538 ± 5.475 | | 16.824 ± 6.038 | | 21.154 ± 7.656 | | 0.4193 | 0.6669 | 0.4509 | 0.5426 |
| A2_C | 13.824 ± 3.245 | | 22.846 ± 7.577 | | 16.471 ± 4.678 | | 22.538 ± 4.825 | | 0.2607 | 0.3969 | 0.6024 | 0.9715 |
| A1_2_3tru_L | 73.235 ± 12.066 | | 40.538 ± 16.959 | | 58.765 ± 11.555 | | 53.769 ± 14.678 | | 0.1301 | 0.7953 | 0.3624 | 0.5087 |
| A1_2_3tru_C | 46.706 ± 8.724 | | 44.538 ± 15.047 | | 38.941 ± 7.635 | | 49.923 ± 11.549 | | 0.9000 | 0.4335 | 0.4040 | 0.7788 |
| A24cd_L | 4.706 ± 2.183 | | 0.077 ± 0.074 | | 4.118 ± 2.164 | | 3.154 ± 1.309 | | 0.0842 | 0.7353 | 0.8178 | 0.0456 |
| A24cd_C | 9.000 ± 4.582 | | 5.692 ± 3.387 | | 8.412 ± 4.154 | | 0.231 ± 0.160 | | 0.5997 | 0.1074 | 0.9065 | 0.1498 |
| A23c_L | 9.176 ± 2.980 | | 29.846 ± 9.369 | | 14.765 ± 2.770 | | 16.923 ± 6.355 | | 0.0325 | 0.7469 | 0.2007 | 0.1252 |
| A23c_C | 19.059 ± 6.107 | | 22.462 ± 7.517 | | 16.176 ± 5.058 | | 26.538 ± 7.454 | | 0.7343 | 0.2604 | 0.7482 | 0.6063 |
| mPMtha_L | 6.647 ± 3.910 | | 2.769 ± 2.000 | | 4.824 ± 3.396 | | 3.692 ± 2.119 | | 0.4418 | 0.8014 | 0.7020 | 0.5076 |
| mPMtha_C | 9.059 ± 4.091 | | 11.615 ± 4.042 | | 5.941 ± 2.924 | | 4.231 ± 2.416 | | 0.6768 | 0.6793 | 0.5469 | 0.1644 |
| Stha_L | 0.000 ± 0.000 | | 1.077 ± 0.709 | | 0.059 ± 0.057 | | 3.000 ± 1.657 | | 0.1044 | 0.0601 | 0.3322 | 0.2987 |
| Stha_C | 6.000 ± 3.987 | | 3.000 ± 1.764 | | 5.353 ± 2.286 | | 2.846 ± 2.038 | | 0.5519 | 0.4503 | 0.8944 | 0.9512 |

| **Table S14. Nodal betweenness between fast-recovery and slow-recovery groups (right lesion)** | | | | | | | | | | | | |
| --- | --- | --- | --- | --- | --- | --- | --- | --- | --- | --- | --- | --- |
| Nodal | | Preoperative | | | Postoperative | | | *p* value (Two-sample t test) | | | *p* value (Paired t test) | |
|  |  | Fast-recovery | | Slow-recovery | Fast-recovery | Slow-recovery | | Preoperative | | Postoperative | Fast-recovery | Slow-recovery |
|  |  |  |  |  |  |  |  | Fast-recovery *vs.* Slow-recovery | | Fast-recovery *vs.* Slow-recovery | Preoperative *vs.* Postoperative | Preoperative *vs.* Postoperative |
| A6dl_C | 28.289 ± 8.263 | | 34.833 ± 17.001 | | 20.011 ± 4.588 | | 33.333 ± 12.301 | | 0.7585 | 0.3761 | 0.4720 | 0.9529 |
| A6m_C | 37.589 ± 9.672 | | 32.667 ± 9.845 | | 19.678 ± 9.499 | | 38.667 ± 16.769 | | 0.7515 | 0.3896 | 0.3639 | 0.7316 |
| A6vl_C | 1.667 ± 1.521 | | 10.500 ± 6.288 | | 1.500 ± 1.369 | | 4.167 ± 2.733 | | 0.2410 | 0.4443 | 0.9485 | 0.4331 |
| A6cdl_C | 29.711 ± 9.853 | | 47.833 ± 20.765 | | 28.678 ± 12.050 | | 52.667 ± 20.592 | | 0.4881 | 0.3803 | 0.9031 | 0.8515 |
| A4ul_L | 83.133 ± 16.359 | | 66.833 ± 13.706 | | 58.033 ± 14.842 | | 97.667 ± 21.797 | | 0.5016 | 0.2000 | 0.0766 | 0.2076 |
| A4ul_C | 22.000 ± 13.300 | | 56.667 ± 13.725 | | 92.511 ± 14.499 | | 53.667 ± 14.323 | | 0.1288 | 0.1125 | 0.0129 | 0.8991 |
| A4t_L | 16.333 ± 12.529 | | 37.500 ± 20.549 | | 33.056 ± 7.647 | | 44.667 ± 19.281 | | 0.4407 | 0.6204 | 0.4355 | 0.5624 |
| A4t_C | 135.856 ± 20.103 | | 116.667 ± 12.059 | | 105.767 ± 31.689 | | 92.667 ± 23.873 | | 0.4721 | 0.7693 | 0.0934 | 0.4186 |
| A4tl_L | 0.667 ± 0.609 | | 0.667 ± 0.451 | | 0.167 ± 0.152 | | 1.500 ± 1.369 | | 1.0000 | 0.3977 | 0.3632 | 0.4618 |
| A4tl_C | 17.033 ± 5.103 | | 12.000 ± 4.655 | | 20.578 ± 6.479 | | 8.000 ± 4.619 | | 0.5210 | 0.1796 | 0.1890 | 0.6016 |
| A6cvl_L | 21.022 ± 6.329 | | 21.000 ± 9.104 | | 5.167 ± 3.593 | | 15.333 ± 5.933 | | 0.9986 | 0.2105 | 0.0973 | 0.6391 |
| A6cvl_C | 40.900 ± 12.583 | | 28.667 ± 10.702 | | 6.833 ± 6.238 | | 0.000 ± 0.000 | | 0.5143 | 0.3409 | 0.0688 | 0.0583 |
| A1_2_3ll_C | 10.844 ± 3.919 | | 14.167 ± 10.012 | | 1.733 ± 0.611 | | 27.000 ± 8.916 | | 0.7836 | 0.0274 | 0.0996 | 0.4922 |
| A4ll_C | 28.167 ± 13.795 | | 94.500 ± 37.784 | | 89.689 ± 35.437 | | 20.000 ± 7.498 | | 0.1631 | 0.1096 | 0.2903 | 0.0924 |
| A1_2_3ulhf_L | 25.922 ± 10.790 | | 24.833 ± 6.202 | | 41.911 ± 6.833 | | 30.167 ± 10.702 | | 0.9379 | 0.4182 | 0.1365 | 0.7062 |
| A1_2_3ulhf_C | 10.444 ± 4.407 | | 17.167 ± 6.673 | | 43.433 ± 14.387 | | 6.333 ± 3.509 | | 0.4606 | 0.0452 | 0.0333 | 0.2344 |
| A1_2_3tonIa_L | 4.333 ± 3.956 | | 0.500 ± 0.204 | | 4.500 ± 3.588 | | 4.500 ± 4.108 | | 0.3977 | 1.0000 | 0.8090 | 0.4247 |
| A1_2_3tonIa_C | 4.333 ± 3.956 | | 0.000 ± 0.000 | | 4.000 ± 3.651 | | 12.167 ± 7.373 | | 0.3409 | 0.3862 | 0.3632 | 0.1923 |
| A2_L | 0.333 ± 0.304 | | 3.833 ± 3.499 | | 0.000 ± 0.000 | | 11.333 ± 6.810 | | 0.3844 | 0.1596 | 0.3632 | 0.3278 |
| A2_C | 17.400 ± 8.722 | | 12.667 ± 6.846 | | 11.833 ± 6.379 | | 31.000 ± 9.854 | | 0.7049 | 0.1669 | 0.3620 | 0.1929 |
| A1_2_3tru_L | 50.544 ± 11.924 | | 19.167 ± 5.123 | | 80.944 ± 21.411 | | 33.833 ± 18.917 | | 0.0518 | 0.1632 | 0.0790 | 0.4769 |
| A1_2_3tru_C | 51.400 ± 7.169 | | 51.667 ± 16.232 | | 19.933 ± 4.338 | | 74.833 ± 22.224 | | 0.9893 | 0.0513 | 0.0376 | 0.5596 |
| A24cd_L | 2.544 ± 0.660 | | 0.333 ± 0.304 | | 19.333 ± 10.335 | | 9.333 ± 7.813 | | 0.0196 | 0.4971 | 0.2037 | 0.3451 |
| A24cd_C | 0.000 ± 0.000 | | 0.000 ± 0.000 | | 17.578 ± 5.297 | | 4.667 ± 4.080 | | - | 0.1084 | 0.0291 | 0.3443 |
| A23c_L | 11.022 ± 5.476 | | 21.000 ± 6.051 | | 23.089 ± 6.132 | | 47.5 ± 11.817 | | 0.2905 | 0.1251 | 0.3271 | 0.1553 |
| A23c_C | 9.333 ± 5.470 | | 20.000 ± 6.553 | | 13.667 ± 8.465 | | 10.000 ± 5.817 | | 0.2806 | 0.7512 | 0.4405 | 0.4158 |
| mPMtha_L | 0.000 ± 0.000 | | 11.167 ± 4.907 | | 2.500 ± 2.282 | | 6.833 ± 3.218 | | 0.0645 | 0.3397 | 0.3632 | 0.5028 |
| mPMtha_C | 1.000 ± 0.913 | | 4.000 ± 3.651 | | 20.178 ± 8.247 | | 32.500 ± 25.538 | | 0.4835 | 0.6840 | 0.1001 | 0.2888 |
| Stha_L | 0.000 ± 0.000 | | 7.333 ± 4.260 | | 0.500 ± 0.456 | | 3.667 ± 2.143 | | 0.1472 | 0.2165 | 0.3632 | 0.5781 |
| Stha_C | 4.333 ± 3.956 | | 4.667 ± 4.260 | | 0.000 ± 0.000 | | 4.333 ± 3.956 | | 0.9593 | 0.3409 | 0.3632 | 0.9637 |

The *p* value with label ‘*’ meant the result was significant after Bonferroni correction (p < 0.05/30).

The *p* value with label ‘*’ meant the result was significant after Bonferroni correction (p < 0.05/30).

| **Table S15. Nodal clustering coefficient between fast-recovery and slow-recovery groups (left lesion)** | | | | | | | | |
| --- | --- | --- | --- | --- | --- | --- | --- | --- |
| Nodal | Preoperative | | Postoperative | | *p* value (Two-sample t test) | | *p* value (Paired t test) | |
|  | Fast-recovery | Slow-recovery | Fast-recovery | Slow-recovery | Preoperative | Postoperative | Fast-recovery | Slow-recovery |
|  |  |  |  |  | Fast-recovery *vs.* Slow-recovery | Fast-recovery *vs.* Slow-recovery | Preoperative *vs.* Postoperative | Preoperative *vs.* Postoperative |
| A6dl_C | 0.285 ± 0.025 | 0.353 ± 0.054 | 0.349 ± 0.037 | 0.491 ± 0.039 | 0.2434 | 0.0185 | 0.1248 | 0.0690 |
| A6m_C | 0.240 ± 0.033 | 0.314 ± 0.054 | 0.227 ± 0.016 | 0.288 ± 0.039 | 0.2474 | 0.1342 | 0.7474 | 0.6961 |
| A6vl_C | 0.571 ± 0.026 | 0.678 ± 0.035 | 0.523 ± 0.045 | 0.580 ± 0.072 | 0.0224 | 0.5046 | 0.3546 | 0.2402 |
| A6cdl_C | 0.246 ± 0.015 | 0.242 ± 0.015 | 0.266 ± 0.017 | 0.303 ± 0.022 | 0.8816 | 0.1996 | 0.2671 | 0.0602 |
| A4ul_L | 0.335 ± 0.029 | 0.185 ± 0.032 | 0.238 ± 0.016 | 0.293 ± 0.041 | 0.0025 | 0.1947 | 0.0047 | 0.0492 |
| A4ul_C | 0.323 ± 0.026 | 0.430 ± 0.022 | 0.368 ± 0.024 | 0.387 ± 0.032 | 0.0069 | 0.6404 | 0.2166 | 0.2864 |
| A4t_L | 0.288 ± 0.042 | 0.208 ± 0.062 | 0.381 ± 0.054 | 0.338 ± 0.063 | 0.2937 | 0.6174 | 0.1439 | 0.2179 |
| A4t_C | 0.233 ± 0.019 | 0.253 ± 0.026 | 0.245 ± 0.020 | 0.234 ± 0.018 | 0.5388 | 0.7082 | 0.6524 | 0.5707 |
| A4tl_L | 0.099 ± 0.046 | 0.137 ± 0.070 | 0.116 ± 0.061 | 0.144 ± 0.073 | 0.6478 | 0.7731 | 0.7937 | 0.9319 |
| A4tl_C | 0.062 ± 0.030 | 0.120 ± 0.056 | 0.103 ± 0.039 | 0.159 ± 0.070 | 0.3570 | 0.4730 | 0.4024 | 0.5925 |
| A6cvl_L | 0.117 ± 0.062 | 0.157 ± 0.076 | 0.221 ± 0.073 | 0.088 ± 0.058 | 0.6912 | 0.1974 | 0.3551 | 0.5045 |
| A6cvl_C | 0.398 ± 0.066 | 0.345 ± 0.074 | 0.243 ± 0.065 | 0.224 ± 0.055 | 0.6132 | 0.8313 | 0.1083 | 0.0709 |
| A1_2_3ll_C | 0.289 ± 0.028 | 0.368 ± 0.054 | 0.335 ± 0.041 | 0.305 ± 0.062 | 0.1877 | 0.6858 | 0.3133 | 0.5180 |
| A4ll_C | 0.319 ± 0.038 | 0.374 ± 0.071 | 0.386 ± 0.037 | 0.331 ± 0.054 | 0.4880 | 0.4106 | 0.1833 | 0.6668 |
| A1_2_3ulhf_L | 0.259 ± 0.047 | 0.279 ± 0.054 | 0.343 ± 0.066 | 0.272 ± 0.046 | 0.7918 | 0.4330 | 0.2748 | 0.9364 |
| A1_2_3ulhf_C | 0.351 ± 0.042 | 0.421 ± 0.033 | 0.399 ± 0.048 | 0.423 ± 0.052 | 0.2391 | 0.7460 | 0.4131 | 0.9660 |
| A1_2_3tonIa_L | 0.168 ± 0.064 | 0.158 ± 0.065 | 0.114 ± 0.060 | 0.078 ± 0.053 | 0.9166 | 0.6721 | 0.4552 | 0.1623 |
| A1_2_3tonIa_C | 0.146 ± 0.053 | 0.161 ± 0.072 | 0.140 ± 0.051 | 0.204 ± 0.069 | 0.8664 | 0.4644 | 0.8905 | 0.6874 |
| A2_L | 0.255 ± 0.057 | 0.387 ± 0.066 | 0.340 ± 0.066 | 0.386 ± 0.074 | 0.1536 | 0.6607 | 0.2689 | 0.9899 |
| A2_C | 0.396 ± 0.044 | 0.491 ± 0.048 | 0.433 ± 0.042 | 0.379 ± 0.041 | 0.1685 | 0.3882 | 0.6059 | 0.0928 |
| A1_2_3tru_L | 0.255 ± 0.031 | 0.304 ± 0.047 | 0.263 ± 0.018 | 0.317 ± 0.030 | 0.3966 | 0.1211 | 0.8529 | 0.7337 |
| A1_2_3tru_C | 0.228 ± 0.026 | 0.298 ± 0.025 | 0.272 ± 0.015 | 0.278 ± 0.022 | 0.0784 | 0.8379 | 0.1763 | 0.6402 |
| A24cd_L | 0.237 ± 0.065 | 0.071 ± 0.047 | 0.121 ± 0.047 | 0.176 ± 0.063 | 0.0696 | 0.4981 | 0.2029 | 0.2257 |
| A24cd_C | 0.318 ± 0.062 | 0.457 ± 0.066 | 0.325 ± 0.064 | 0.446 ± 0.091 | 0.1531 | 0.2924 | 0.9422 | 0.8932 |
| A23c_L | 0.200 ± 0.057 | 0.147 ± 0.048 | 0.192 ± 0.047 | 0.190 ± 0.053 | 0.5136 | 0.9884 | 0.9079 | 0.5968 |
| A23c_C | 0.333 ± 0.042 | 0.410 ± 0.059 | 0.371 ± 0.043 | 0.311 ± 0.050 | 0.2974 | 0.3875 | 0.5721 | 0.0911 |
| mPMtha_L | 0.141 ± 0.063 | 0.241 ± 0.081 | 0.257 ± 0.071 | 0.426 ± 0.092 | 0.3517 | 0.1629 | 0.2488 | 0.0710 |
| mPMtha_C | 0.341 ± 0.061 | 0.185 ± 0.057 | 0.259 ± 0.071 | 0.306 ± 0.086 | 0.0916 | 0.6853 | 0.4467 | 0.2409 |
| Stha_L | 0.300 ± 0.088 | 0.353 ± 0.091 | 0.258 ± 0.078 | 0.297 ± 0.085 | 0.6876 | 0.7514 | 0.7293 | 0.3885 |
| Stha_C | 0.360 ± 0.067 | 0.375 ± 0.085 | 0.221 ± 0.062 | 0.400 ± 0.077 | 0.8951 | 0.0886 | 0.1693 | 0.7927 |

| **Table S16. Nodal clustering coefficient between fast-recovery and slow-recovery groups (right lesion)** | | | | | | | | |
| --- | --- | --- | --- | --- | --- | --- | --- | --- |
| Nodal | Preoperative | | Postoperative | | *p* value (Two-sample t test) | | *p* value (Paired t test) | |
|  | Fast-recovery | Slow-recovery | Fast-recovery | Slow-recovery | Preoperative | Postoperative | Fast-recovery | Slow-recovery |
|  |  |  |  |  | Fast-recovery *vs.* Slow-recovery | Fast-recovery *vs.* Slow-recovery | Preoperative *vs.* Postoperative | Preoperative *vs.* Postoperative |
| A6dl_C | 0.258 ± 0.035 | 0.388 ± 0.099 | 0.207 ± 0.021 | 0.185 ± 0.035 | 0.2864 | 0.6415 | 0.2085 | 0.1845 |
| A6m_C | 0.226 ± 0.021 | 0.303 ± 0.065 | 0.274 ± 0.055 | 0.257 ± 0.038 | 0.3294 | 0.8159 | 0.3689 | 0.5506 |
| A6vl_C | 0.674 ± 0.080 | 0.427 ± 0.106 | 0.579 ± 0.084 | 0.399 ± 0.096 | 0.1195 | 0.2296 | 0.3768 | 0.7994 |
| A6cdl_C | 0.460 ± 0.076 | 0.284 ± 0.035 | 0.313 ± 0.040 | 0.248 ± 0.036 | 0.0843 | 0.3027 | 0.0453 | 0.3945 |
| A4ul_L | 0.297 ± 0.031 | 0.230 ± 0.021 | 0.287 ± 0.029 | 0.215 ± 0.023 | 0.1388 | 0.1060 | 0.8395 | 0.6161 |
| A4ul_C | 0.589 ± 0.081 | 0.325 ± 0.028 | 0.259 ± 0.030 | 0.315 ± 0.024 | 0.0185 | 0.2191 | 0.0211 | 0.8300 |
| A4t_L | 0.651 ± 0.125 | 0.220 ± 0.042 | 0.370 ± 0.080 | 0.278 ± 0.093 | 0.0134 | 0.5064 | 0.0521 | 0.5621 |
| A4t_C | 0.194 ± 0.018 | 0.182 ± 0.057 | 0.300 ± 0.076 | 0.246 ± 0.033 | 0.8658 | 0.5648 | 0.1911 | 0.4706 |
| A4tl_L | 0.000 ± 0.000 | 0.000 ± 0.000 | 0.000 ± 0.000 | 0.168 ± 0.116 | - | 0.2139 | - | 0.2418 |
| A4tl_C | 0.158 ± 0.107 | 0.083 ± 0.048 | 0.218 ± 0.082 | 0.202 ± 0.117 | 0.5732 | 0.9203 | 0.5188 | 0.1777 |
| A6cvl_L | 0.180 ± 0.054 | 0.169 ± 0.082 | 0.447 ± 0.160 | 0.235 ± 0.080 | 0.9238 | 0.3062 | 0.1381 | 0.2168 |
| A6cvl_C | 0.482 ± 0.085 | 0.206 ± 0.070 | 0.157 ± 0.105 | 0.260 ± 0.150 | 0.0448 | 0.6192 | 0.0134 | 0.6850 |
| A1_2_3ll_C | 0.216 ± 0.043 | 0.214 ± 0.110 | 0.433 ± 0.059 | 0.282 ± 0.095 | 0.9847 | 0.2472 | 0.0109 | 0.7389 |
| A4ll_C | 0.393 ± 0.064 | 0.192 ± 0.053 | 0.287 ± 0.024 | 0.264 ± 0.070 | 0.0518 | 0.7863 | 0.2544 | 0.2606 |
| A1_2_3ulhf_L | 0.187 ± 0.042 | 0.190 ± 0.039 | 0.278 ± 0.070 | 0.277 ± 0.081 | 0.9646 | 0.9924 | 0.2573 | 0.3266 |
| A1_2_3ulhf_C | 0.563 ± 0.039 | 0.373 ± 0.081 | 0.342 ± 0.065 | 0.447 ± 0.060 | 0.0817 | 0.3059 | 0.0039 | 0.4462 |
| A1_2_3tonIa_L | 0.000 ± 0.000 | 0.079 ± 0.046 | 0.000 ± 0.000 | 0.118 ± 0.108 | 0.1481 | 0.3409 | - | 0.7927 |
| A1_2_3tonIa_C | 0.081 ± 0.073 | 0.076 ± 0.069 | 0.455 ± 0.123 | 0.201 ± 0.089 | 0.9682 | 0.1564 | 0.0685 | 0.1329 |
| A2_L | 0.165 ± 0.095 | 0.200 ± 0.090 | 0.107 ± 0.098 | 0.211 ± 0.102 | 0.8116 | 0.5176 | 0.7489 | 0.9498 |
| A2_C | 0.495 ± 0.045 | 0.422 ± 0.052 | 0.565 ± 0.092 | 0.439 ± 0.080 | 0.3620 | 0.3692 | 0.5667 | 0.8271 |
| A1_2_3tru_L | 0.242 ± 0.019 | 0.259 ± 0.052 | 0.242 ± 0.058 | 0.317 ± 0.047 | 0.7862 | 0.3784 | 0.9967 | 0.4442 |
| A1_2_3tru_C | 0.342 ± 0.026 | 0.250 ± 0.024 | 0.284 ± 0.033 | 0.304 ± 0.093 | 0.0389 | 0.8539 | 0.0608 | 0.5620 |
| A24cd_L | 0.340 ± 0.048 | 0.455 ± 0.110 | 0.452 ± 0.113 | 0.366 ± 0.112 | 0.4043 | 0.6301 | 0.4932 | 0.5480 |
| A24cd_C | 0.103 ± 0.094 | 0.220 ± 0.127 | 0.201 ± 0.085 | 0.160 ± 0.093 | 0.5160 | 0.7763 | 0.5828 | 0.1747 |
| A23c_L | 0.406 ± 0.056 | 0.350 ± 0.081 | 0.313 ± 0.061 | 0.270 ± 0.073 | 0.6148 | 0.6869 | 0.2861 | 0.5905 |
| A23c_C | 0.136 ± 0.092 | 0.259 ± 0.106 | 0.056 ± 0.051 | 0.320 ± 0.102 | 0.4431 | 0.0609 | 0.5540 | 0.5998 |
| mPMtha_L | 0.391 ± 0.165 | 0.259 ± 0.086 | 0.043 ± 0.039 | 0.098 ± 0.057 | 0.5320 | 0.4837 | 0.1475 | 0.1215 |
| mPMtha_C | 0.282 ± 0.094 | 0.256 ± 0.148 | 0.242 ± 0.090 | 0.206 ± 0.105 | 0.8988 | 0.8155 | 0.7252 | 0.7595 |
| Stha_L | 0.239 ± 0.138 | 0.267 ± 0.126 | 0.252 ± 0.146 | 0.482 ± 0.120 | 0.8921 | 0.2945 | 0.9488 | 0.3867 |
| Stha_C | 0.453 ± 0.136 | 0.263 ± 0.123 | 0.510 ± 0.155 | 0.120 ± 0.078 | 0.3661 | 0.0669 | 0.0877 | 0.4952 |

The *p* value with label ‘*’ meant the result was significant after Bonferroni correction (p < 0.05/30).

| **Table S17. Nodal vulnerability between fast-recovery and slow-recovery groups (left lesion)** | | | | | | | | |
| --- | --- | --- | --- | --- | --- | --- | --- | --- |
| Nodal | Preoperative | | Postoperative | | *p* value (Two-sample t test) | | *p* value (Paired t test) | |
|  | Fast-recovery | Slow-recovery | Fast-recovery | Slow-recovery | Preoperative | Postoperative | Fast-recovery | Slow-recovery |
|  |  |  |  |  | Fast-recovery *vs.* Slow-recovery | Fast-recovery *vs.* Slow-recovery | Preoperative *vs.* Postoperative | Preoperative *vs.* Postoperative |
| A6dl_C | 0.036 ± 0.008 | 0.047 ± 0.023 | 0.027 ± 0.008 | 0.013 ± 0.005 | 0.6396 | 0.2079 | 0.3883 | 0.1368 |
| A6m_C | 0.066 ± 0.021 | 0.049 ± 0.018 | 0.044 ± 0.006 | 0.035 ± 0.009 | 0.5774 | 0.4042 | 0.3279 | 0.4919 |
| A6vl_C | 0.004 ± 0.002 | 0.001 ± 0.003 | 0.001 ± 0.002 | 0.005 ± 0.004 | 0.4884 | 0.3874 | 0.1530 | 0.5036 |
| A6cdl_C | 0.067 ± 0.019 | 0.098 ± 0.021 | 0.062 ± 0.011 | 0.053 ± 0.009 | 0.2960 | 0.5815 | 0.7362 | 0.0180 |
| A4ul_L | 0.054 ± 0.020 | 0.134 ± 0.026 | 0.101 ± 0.016 | 0.068 ± 0.017 | 0.0218 | 0.1817 | 0.1195 | 0.0376 |
| A4ul_C | 0.040 ± 0.008 | 0.027 ± 0.004 | 0.031 ± 0.004 | 0.037 ± 0.007 | 0.2093 | 0.4422 | 0.2636 | 0.2774 |
| A4t_L | 0.088 ± 0.022 | 0.039 ± 0.024 | 0.037 ± 0.007 | 0.032 ± 0.020 | 0.1552 | 0.8131 | 0.0346 | 0.6492 |
| A4t_C | 0.087 ± 0.022 | 0.075 ± 0.016 | 0.082 ± 0.014 | 0.067 ± 0.012 | 0.6978 | 0.4752 | 0.8516 | 0.6838 |
| A4tl_L | -0.038 ± 0.006 | -0.045 ± 0.008 | -0.041 ± 0.006 | -0.045 ± 0.008 | 0.5095 | 0.7128 | 0.6793 | 0.9793 |
| A4tl_C | -0.014 ± 0.007 | -0.020 ± 0.009 | -0.017 ± 0.008 | -0.012 ± 0.009 | 0.5838 | 0.6698 | 0.7268 | 0.3783 |
| A6cvl_L | -0.032 ± 0.007 | -0.002 ± 0.018 | -0.027 ± 0.009 | -0.040 ± 0.008 | 0.1159 | 0.3610 | 0.6865 | 0.1153 |
| A6cvl_C | 0.005 ± 0.005 | -0.005 ± 0.009 | 0.002 ± 0.009 | 0.006 ± 0.014 | 0.3147 | 0.8497 | 0.7601 | 0.4449 |
| A1_2_3ll_C | 0.025 ± 0.005 | 0.042 ± 0.016 | 0.023 ± 0.005 | 0.046 ± 0.023 | 0.2983 | 0.3118 | 0.6834 | 0.9008 |
| A4ll_C | 0.037 ± 0.017 | 0.012 ± 0.010 | 0.013 ± 0.004 | 0.020 ± 0.012 | 0.2816 | 0.5497 | 0.2186 | 0.5073 |
| A1_2_3ulhf_L | 0.034 ± 0.014 | 0.040 ± 0.012 | 0.030 ± 0.012 | 0.041 ± 0.015 | 0.7626 | 0.5565 | 0.7912 | 0.9486 |
| A1_2_3ulhf_C | 0.029 ± 0.008 | 0.027 ± 0.009 | 0.021 ± 0.008 | 0.022 ± 0.011 | 0.8574 | 0.9690 | 0.4687 | 0.7185 |
| A1_2_3tonIa_L | -0.032 ± 0.008 | -0.040 ± 0.008 | -0.034 ± 0.008 | -0.032 ± 0.008 | 0.4523 | 0.9068 | 0.8582 | 0.4179 |
| A1_2_3tonIa_C | -0.013 ± 0.006 | -0.025 ± 0.010 | -0.030 ± 0.005 | -0.007 ± 0.009 | 0.2790 | 0.0290 | 0.0561 | 0.1883 |
| A2_L | 0.008 ± 0.011 | 0.012 ± 0.008 | 0.014 ± 0.009 | 0.014 ± 0.010 | 0.8360 | 0.9774 | 0.7030 | 0.8588 |
| A2_C | 0.008 ± 0.004 | 0.027 ± 0.011 | 0.019 ± 0.006 | 0.029 ± 0.015 | 0.0990 | 0.5630 | 0.1252 | 0.8750 |
| A1_2_3tru_L | 0.099 ± 0.022 | 0.047 ± 0.022 | 0.073 ± 0.015 | 0.071 ± 0.028 | 0.1268 | 0.9449 | 0.3831 | 0.4839 |
| A1_2_3tru_C | 0.039 ± 0.007 | 0.040 ± 0.009 | 0.038 ± 0.005 | 0.050 ± 0.009 | 0.9396 | 0.2611 | 0.8974 | 0.4621 |
| A24cd_L | -0.015 ± 0.008 | -0.024 ± 0.006 | -0.014 ± 0.007 | -0.016 ± 0.009 | 0.3989 | 0.8821 | 0.9789 | 0.3123 |
| A24cd_C | 0.004 ± 0.012 | -0.003 ± 0.003 | -0.005 ± 0.007 | -0.006 ± 0.005 | 0.6468 | 0.9312 | 0.4399 | 0.5612 |
| A23c_L | -0.006 ± 0.010 | 0.038 ± 0.022 | 0.012 ± 0.010 | 0.027 ± 0.025 | 0.0672 | 0.5526 | 0.2905 | 0.5379 |
| A23c_C | 0.035 ± 0.010 | 0.025 ± 0.008 | 0.020 ± 0.008 | 0.034 ± 0.008 | 0.5039 | 0.2141 | 0.2885 | 0.3735 |
| mPMtha_L | -0.010 ± 0.009 | -0.007 ± 0.009 | -0.007 ± 0.007 | -0.006 ± 0.010 | 0.8645 | 0.9174 | 0.8169 | 0.8684 |
| mPMtha_C | 0.011 ± 0.008 | 0.014 ± 0.008 | 0.005 ± 0.006 | 0.005 ± 0.008 | 0.7966 | 0.9984 | 0.5593 | 0.4773 |
| Stha_L | -0.022 ± 0.008 | -0.008 ± 0.006 | -0.016 ± 0.007 | -0.017 ± 0.009 | 0.2094 | 0.9421 | 0.5853 | 0.4807 |
| Stha_C | 0.001 ± 0.008 | 0.003 ± 0.005 | -0.004 ± 0.008 | 0.005 ± 0.005 | 0.8486 | 0.4229 | 0.6866 | 0.6489 |

The *p* value with label ‘*’ meant the result was significant after Bonferroni correction (p < 0.05/30).

| **Table S18. Nodal vulnerability between fast-recovery and slow-recovery groups (right lesion)** | | | | | | | | |
| --- | --- | --- | --- | --- | --- | --- | --- | --- |
| Nodal | Preoperative | | Postoperative | | *p* value (Two-sample t test) | | *p* value (Paired t test) | |
|  | Fast-recovery | Slow-recovery | Fast-recovery | Slow-recovery | Preoperative | Postoperative | Fast-recovery | Slow-recovery |
|  |  |  |  |  | Fast-recovery *vs.* Slow-recovery | Fast-recovery *vs.* Slow-recovery | Preoperative *vs.* Postoperative | Preoperative *vs.* Postoperative |
| A6dl_C | 0.200 ± 0.035 | 0.201 ± 0.050 | 0.126 ± 0.015 | 0.125 ± 0.028 | 0.9948 | 0.9801 | 0.0891 | 0.1814 |
| A6m_C | 0.055 ± 0.037 | 0.039 ± 0.018 | 0.014 ± 0.004 | 0.046 ± 0.031 | 0.7196 | 0.3818 | 0.3298 | 0.7370 |
| A6vl_C | -0.003 ± 0.002 | 0.004 ± 0.003 | -0.003 ± 0.002 | 0.004 ± 0.003 | 0.0717 | 0.0687 | 0.9992 | 0.9572 |
| A6cdl_C | 0.026 ± 0.015 | 0.080 ± 0.037 | 0.034 ± 0.014 | 0.065 ± 0.032 | 0.2413 | 0.4444 | 0.3335 | 0.7031 |
| A4ul_L | 0.052 ± 0.009 | 0.083 ± 0.036 | 0.036 ± 0.008 | 0.109 ± 0.041 | 0.4616 | 0.1371 | 0.0288 | 0.6488 |
| A4ul_C | 0.003 ± 0.012 | 0.023 ± 0.007 | 0.039 ± 0.009 | 0.055 ± 0.022 | 0.2028 | 0.5344 | 0.0150 | 0.1442 |
| A4t_L | 0.007 ± 0.007 | 0.054 ± 0.044 | 0.033 ± 0.006 | 0.024 ± 0.009 | 0.3618 | 0.4397 | 0.1118 | 0.5154 |
| A4t_C | 0.249 ± 0.042 | 0.135 ± 0.040 | 0.152 ± 0.050 | 0.151 ± 0.044 | 0.1023 | 0.9926 | 0.0015* | 0.8636 |
| A4tl_L | -0.012 ± 0.002 | -0.011 ± 0.003 | -0.019 ± 0.002 | -0.009 ± 0.003 | 0.8029 | 0.0414 | 0.0635 | 0.6385 |
| A4tl_C | 0.016 ± 0.011 | 0.003 ± 0.005 | 0.013 ± 0.010 | -0.008 ± 0.008 | 0.3402 | 0.1737 | 0.0944 | 0.2640 |
| A6cvl_L | 0.044 ± 0.021 | 0.013 ± 0.009 | 0.002 ± 0.004 | 0.006 ± 0.004 | 0.2396 | 0.5299 | 0.1422 | 0.5409 |
| A6cvl_C | 0.055 ± 0.019 | 0.026 ± 0.013 | -0.010 ± 0.010 | -0.018 ± 0.004 | 0.2692 | 0.5428 | 0.0687 | 0.0294 |
| A1_2_3ll_C | 0.001 ± 0.004 | -0.002 ± 0.005 | 0.001 ± 0.002 | 0.005 ± 0.007 | 0.7088 | 0.5790 | 0.9638 | 0.5011 |
| A4ll_C | 0.019 ± 0.010 | 0.154 ± 0.078 | 0.036 ± 0.013 | 0.026 ± 0.011 | 0.1478 | 0.5980 | 0.5279 | 0.1825 |
| A1_2_3ulhf_L | 0.042 ± 0.021 | 0.018 ± 0.011 | 0.071 ± 0.008 | 0.044 ± 0.018 | 0.3728 | 0.2416 | 0.1413 | 0.1119 |
| A1_2_3ulhf_C | -0.004 ± 0.003 | 0.002 ± 0.010 | 0.044 ± 0.018 | 0.010 ± 0.014 | 0.6491 | 0.2056 | 0.0714 | 0.6656 |
| A1_2_3tonIa_L | -0.006 ± 0.007 | -0.004 ± 0.007 | 0.020 ± 0.011 | 0.005 ± 0.011 | 0.8900 | 0.3661 | 0.0625 | 0.2704 |
| A1_2_3tonIa_C | -0.013 ± 0.009 | -0.026 ± 0.002 | -0.015 ± 0.009 | -0.004 ± 0.012 | 0.2099 | 0.4999 | 0.2508 | 0.1898 |
| A2_L | -0.007 ± 0.002 | -0.005 ± 0.003 | -0.014 ± 0.002 | 0.011 ± 0.012 | 0.6455 | 0.0931 | 0.1835 | 0.2328 |
| A2_C | 0.018 ± 0.015 | -0.003 ± 0.004 | -0.002 ± 0.006 | 0.009 ± 0.010 | 0.2504 | 0.4338 | 0.1433 | 0.3374 |
| A1_2_3tru_L | 0.062 ± 0.025 | 0.027 ± 0.010 | 0.110 ± 0.026 | 0.044 ± 0.022 | 0.2591 | 0.1066 | 0.2754 | 0.4750 |
| A1_2_3tru_C | 0.025 ± 0.002 | 0.051 ± 0.030 | 0.014 ± 0.003 | 0.055 ± 0.037 | 0.4393 | 0.3398 | 0.0548 | 0.9496 |
| A24cd_L | -0.005 ± 0.002 | -0.009 ± 0.003 | 0.030 ± 0.018 | 0.002 ± 0.011 | 0.4002 | 0.2530 | 0.1545 | 0.4818 |
| A24cd_C | -0.020 ± 0.002 | -0.014 ± 0.003 | 0.026 ± 0.014 | 0.010 ± 0.011 | 0.1080 | 0.4165 | 0.0344 | 0.1017 |
| A23c_L | 0.005 ± 0.005 | 0.013 ± 0.007 | 0.016 ± 0.007 | 0.044 ± 0.016 | 0.4549 | 0.1621 | 0.3450 | 0.1643 |
| A23c_C | 0.027 ± 0.009 | 0.021 ± 0.009 | 0.003 ± 0.014 | -0.008 ± 0.007 | 0.6980 | 0.5304 | 0.2312 | 0.0715 |
| mPMtha_L | -0.008 ± 0.002 | 0.009 ± 0.004 | -0.004 ± 0.005 | 0.001 ± 0.004 | 0.0080 | 0.4705 | 0.5309 | 0.1521 |
| mPMtha_C | 0.005 ± 0.006 | 0.006 ± 0.010 | 0.002 ± 0.005 | 0.046 ± 0.039 | 0.9581 | 0.3256 | 0.6643 | 0.3287 |
| Stha_L | -0.006 ± 0.002 | 0.004 ± 0.006 | -0.006 ± 0.001 | 0.003 ± 0.003 | 0.1724 | 0.0414 | 0.8177 | 0.9165 |
| Stha_C | -0.005 ± 0.009 | 0.010 ± 0.012 | -0.006 ± 0.007 | -0.002 ± 0.010 | 0.3943 | 0.7946 | 0.8739 | 0.6201 |

The *p* value with label ‘*’ meant the result was significant after Bonferroni correction (p < 0.05/30).

| **Table S19. Postoperative variations of Nodal efficiency between fast-recovery and slow-recovery groups (left lesion)** | | | |
| --- | --- | --- | --- |
| Nodal | Fast-recovery | Slow-recovery | *p* value  (Two-sample *t* test) |
| A6dl_C | 0.006 ± 0.010 | -0.016 ± 0.007 | 0.1196 |
| A6m_C | 0.010 ± 0.009 | -0.001 ± 0.017 | 0.5465 |
| A6vl_C | 0.005 ± 0.008 | -0.002 ± 0.007 | 0.5462 |
| A6cdl_C | 0.013 ± 0.010 | -0.016 ± 0.008 | 0.0461 |
| A4ul_L | 0.049 ± 0.007 | -0.015 ± 0.013 | 0.0002* |
| A4ul_C | 0.014 ± 0.006 | -0.007 ± 0.008 | 0.0451 |
| A4t_L | 0.009 ± 0.011 | -0.001 ± 0.015 | 0.6152 |
| A4t_C | 0.025 ± 0.008 | -0.004 ± 0.013 | 0.0592 |
| A4tl_L | -0.003 ± 0.014 | -0.004 ± 0.018 | 0.9492 |
| A4tl_C | -0.003 ± 0.016 | 0.004 ± 0.009 | 0.7579 |
| A6cvl_L | 0.014 ± 0.021 | -0.043 ± 0.019 | 0.0744 |
| A6cvl_C | -0.005 ± 0.013 | 0.004 ± 0.012 | 0.6225 |
| A1_2_3ll_C | 0.007 ± 0.010 | -0.002 ± 0.015 | 0.6122 |
| A4ll_C | 0.004 ± 0.008 | 0.006 ± 0.017 | 0.9047 |
| A1_2_3ulhf_L | 0.023 ± 0.010 | -0.009 ± 0.011 | 0.0509 |
| A1_2_3ulhf_C | 0.010 ± 0.007 | -0.010 ± 0.007 | 0.0809 |
| A1_2_3tonIa_L | -0.004 ± 0.017 | 0.003 ± 0.016 | 0.7713 |
| A1_2_3tonIa_C | -0.011 ± 0.012 | 0.028 ± 0.021 | 0.1213 |
| A2_L | 0.030 ± 0.013 | -0.010 ± 0.012 | 0.0387 |
| A2_C | 0.018 ± 0.005 | -0.010 ± 0.011 | 0.0178 |
| A1_2_3tru_L | 0.028 ± 0.011 | 0.000 ± 0.017 | 0.1825 |
| A1_2_3tru_C | 0.017 ± 0.011 | 0.000 ± 0.013 | 0.3220 |
| A24cd_L | 0.006 ± 0.016 | 0.004 ± 0.016 | 0.9177 |
| A24cd_C | 0.001 ± 0.013 | -0.008 ± 0.010 | 0.6330 |
| A23c_L | 0.028 ± 0.021 | -0.005 ± 0.020 | 0.2858 |
| A23c_C | 0.000 ± 0.006 | -0.003 ± 0.015 | 0.8567 |
| mPMtha_L | 0.019 ± 0.022 | -0.006 ± 0.017 | 0.4167 |
| mPMtha_C | -0.005 ± 0.014 | -0.010 ± 0.018 | 0.8017 |
| Stha_L | 0.018 ± 0.019 | -0.024 ± 0.021 | 0.1637 |
| Stha_C | -0.013 ± 0.016 | 0.000 ± 0.012 | 0.5522 |
| The *p* value with label ‘*’ meant the result was significant after Bonferroni correction (p < 0.05/30). | | | |

| **Table S20. Postoperative variations of Nodal efficiency between fast-recovery and slow-recovery groups (right lesion)** | | | |
| --- | --- | --- | --- |
| Nodal | Fast-recovery | Slow-recovery | *p* value  (Two-sample *t* test) |
| A6dl_C | 0.007 ± 0.012 | 0.006 ± 0.014 | 0.9752 |
| A6m_C | 0.005 ± 0.015 | 0.001 ± 0.010 | 0.8756 |
| A6vl_C | 0.004 ± 0.016 | 0.018 ± 0.005 | 0.4693 |
| A6cdl_C | 0.007 ± 0.016 | 0.017 ± 0.009 | 0.6208 |
| A4ul_L | -0.009 ± 0.018 | 0.025 ± 0.013 | 0.1868 |
| A4ul_C | 0.038 ± 0.005 | 0.009 ± 0.007 | 0.0093 |
| A4t_L | 0.045 ± 0.022 | 0.042 ± 0.022 | 0.9357 |
| A4t_C | -0.022 ± 0.015 | 0.005 ± 0.006 | 0.1591 |
| A4tl_L | 0.000 ± 0.004 | 0.021 ± 0.028 | 0.5137 |
| A4tl_C | -0.006 ± 0.007 | -0.019 ± 0.013 | 0.4256 |
| A6cvl_L | -0.008 ± 0.017 | 0.036 ± 0.019 | 0.1447 |
| A6cvl_C | -0.052 ± 0.025 | -0.020 ± 0.029 | 0.4505 |
| A1_2_3ll_C | 0.007 ± 0.006 | 0.043 ± 0.027 | 0.2614 |
| A4ll_C | 0.014 ± 0.020 | -0.007 ± 0.006 | 0.3718 |
| A1_2_3ulhf_L | 0.009 ± 0.010 | 0.011 ± 0.005 | 0.8727 |
| A1_2_3ulhf_C | -0.005 ± 0.007 | 0.009 ± 0.004 | 0.1711 |
| A1_2_3tonIa_L | -0.048 ± 0.022 | 0.016 ± 0.031 | 0.1571 |
| A1_2_3tonIa_C | 0.009 ± 0.004 | 0.004 ± 0.012 | 0.7608 |
| A2_L | -0.026 ± 0.015 | 0.051 ± 0.025 | 0.0356 |
| A2_C | -0.002 ± 0.008 | 0.003 ± 0.006 | 0.6868 |
| A1_2_3tru_L | 0.001 ± 0.004 | 0.027 ± 0.013 | 0.1322 |
| A1_2_3tru_C | -0.012 ± 0.014 | 0.016 ± 0.020 | 0.3196 |
| A24cd_L | 0.024 ± 0.014 | 0.008 ± 0.012 | 0.4486 |
| A24cd_C | 0.089 ± 0.031 | -0.005 ± 0.016 | 0.0341 |
| A23c_L | -0.001 ± 0.029 | 0.019 ± 0.010 | 0.5764 |
| A23c_C | 0.034 ± 0.035 | -0.010 ± 0.035 | 0.4448 |
| mPMtha_L | 0.032 ± 0.026 | 0.020 ± 0.024 | 0.7617 |
| mPMtha_C | 0.006 ± 0.034 | 0.033 ± 0.031 | 0.6023 |
| Stha_L | 0.097 ± 0.032 | 0.002 ± 0.031 | 0.0826 |
| Stha_C | -0.028 ± 0.023 | 0.015 ± 0.042 | 0.4335 |
| The *p* value with label ‘*’ meant the result was significant after Bonferroni correction (p < 0.05/30). | | | |

| **Table S21. Postoperative variations of Nodal degree centrality between fast-recovery and slow-recovery groups (left lesion)** | | | |
| --- | --- | --- | --- |
| Nodal | Fast-recovery | Slow-recovery | *p* value  (Two-sample *t* test) |
| A6dl_C | -0.146 ± 0.222 | -0.255 ± 0.159 | 0.7175 |
| A6m_C | -0.035 ± 0.270 | 0.458 ± 0.354 | 0.2852 |
| A6vl_C | -0.025 ± 0.101 | 0.161 ± 0.152 | 0.3153 |
| A6cdl_C | 0.192 ± 0.204 | -0.311 ± 0.205 | 0.1091 |
| A4ul_L | 1.210 ± 0.187 | 0.008 ± 0.324 | 0.0028 |
| A4ul_C | 0.122 ± 0.160 | -0.073 ± 0.129 | 0.3879 |
| A4t_L | 0.059 ± 0.300 | 0.181 ± 0.290 | 0.7834 |
| A4t_C | 0.604 ± 0.273 | 0.089 ± 0.270 | 0.2145 |
| A4tl_L | -0.120 ± 0.090 | -0.112 ± 0.101 | 0.9559 |
| A4tl_C | 0.052 ± 0.113 | 0.025 ± 0.102 | 0.8693 |
| A6cvl_L | 0.093 ± 0.187 | -0.261 ± 0.144 | 0.1805 |
| A6cvl_C | -0.053 ± 0.160 | 0.320 ± 0.218 | 0.1827 |
| A1_2_3ll_C | -0.050 ± 0.182 | 0.124 ± 0.213 | 0.5521 |
| A4ll_C | -0.079 ± 0.189 | 0.514 ± 0.239 | 0.0665 |
| A1_2_3ulhf_L | -0.041 ± 0.129 | 0.027 ± 0.138 | 0.7321 |
| A1_2_3ulhf_C | 0.089 ± 0.142 | -0.174 ± 0.137 | 0.2189 |
| A1_2_3tonIa_L | -0.036 ± 0.077 | -0.104 ± 0.106 | 0.6103 |
| A1_2_3tonIa_C | -0.108 ± 0.063 | 0.192 ± 0.159 | 0.0743 |
| A2_L | 0.016 ± 0.160 | -0.098 ± 0.174 | 0.6481 |
| A2_C | 0.234 ± 0.107 | 0.015 ± 0.132 | 0.2196 |
| A1_2_3tru_L | 0.569 ± 0.316 | 0.416 ± 0.391 | 0.7685 |
| A1_2_3tru_C | 0.299 ± 0.324 | 0.058 ± 0.229 | 0.5849 |
| A24cd_L | -0.187 ± 0.124 | 0.264 ± 0.207 | 0.0683 |
| A24cd_C | -0.071 ± 0.136 | -0.096 ± 0.164 | 0.9114 |
| A23c_L | 0.258 ± 0.217 | 0.012 ± 0.255 | 0.4812 |
| A23c_C | -0.123 ± 0.130 | 0.173 ± 0.233 | 0.2670 |
| mPMtha_L | 0.181 ± 0.173 | 0.087 ± 0.105 | 0.6818 |
| mPMtha_C | -0.342 ± 0.156 | -0.005 ± 0.206 | 0.2091 |
| Stha_L | 0.111 ± 0.131 | 0.025 ± 0.117 | 0.6510 |
| Stha_C | -0.353 ± 0.158 | 0.145 ± 0.142 | 0.0371 |
| The *p* value with label ‘*’ meant the result was significant after Bonferroni correction (p < 0.05/30). | | | |

| **Table S22. Postoperative variations of Nodal degree centrality between fast-recovery and slow-recovery groups (right lesion)** | | | |
| --- | --- | --- | --- |
| Nodal | Fast-recovery | Slow-recovery | *p* value  (Two-sample *t* test) |
| A6dl_C | -0.209 ± 0.109 | 0.086 ± 0.342 | 0.4711 |
| A6m_C | 0.222 ± 0.241 | -0.145 ± 0.350 | 0.4487 |
| A6vl_C | -0.280 ± 0.269 | 0.179 ± 0.126 | 0.1889 |
| A6cdl_C | -0.251 ± 0.149 | 0.224 ± 0.168 | 0.0820 |
| A4ul_L | -0.141 ± 0.497 | 0.607 ± 0.371 | 0.2970 |
| A4ul_C | 0.791 ± 0.090 | 0.086 ± 0.125 | 0.0019 |
| A4t_L | 0.851 ± 0.438 | 0.491 ± 0.274 | 0.5384 |
| A4t_C | -0.915 ± 0.324 | -0.004 ± 0.273 | 0.0782 |
| A4tl_L | 0.099 ± 0.063 | 0.128 ± 0.202 | 0.9031 |
| A4tl_C | 0.172 ± 0.110 | -0.200 ± 0.095 | 0.0415 |
| A6cvl_L | -0.998 ± 0.488 | 0.339 ± 0.381 | 0.0769 |
| A6cvl_C | -0.682 ± 0.397 | -0.573 ± 0.227 | 0.8316 |
| A1_2_3ll_C | -0.070 ± 0.202 | 0.591 ± 0.493 | 0.2836 |
| A4ll_C | 0.876 ± 0.599 | -0.506 ± 0.255 | 0.0813 |
| A1_2_3ulhf_L | 0.306 ± 0.128 | 0.027 ± 0.147 | 0.2214 |
| A1_2_3ulhf_C | 0.059 ± 0.079 | 0.022 ± 0.075 | 0.7638 |
| A1_2_3tonIa_L | -0.033 ± 0.021 | -0.149 ± 0.219 | 0.6428 |
| A1_2_3tonIa_C | 0.244 ± 0.065 | 0.206 ± 0.132 | 0.8153 |
| A2_L | -0.478 ± 0.295 | 0.390 ± 0.256 | 0.0703 |
| A2_C | -0.312 ± 0.212 | 0.010 ± 0.076 | 0.2213 |
| A1_2_3tru_L | 0.391 ± 0.365 | 0.479 ± 0.416 | 0.8875 |
| A1_2_3tru_C | -0.340 ± 0.301 | 0.277 ± 0.509 | 0.3640 |
| A24cd_L | 0.291 ± 0.221 | 0.074 ± 0.231 | 0.5497 |
| A24cd_C | 0.665 ± 0.268 | 0.168 ± 0.092 | 0.1408 |
| A23c_L | 0.460 ± 0.428 | 0.462 ± 0.267 | 0.9961 |
| A23c_C | 0.092 ± 0.454 | -0.090 ± 0.263 | 0.7584 |
| mPMtha_L | 0.117 ± 0.276 | -0.180 ± 0.177 | 0.4290 |
| mPMtha_C | 0.148 ± 0.095 | 0.218 ± 0.180 | 0.7577 |
| Stha_L | 0.388 ± 0.179 | 0.270 ± 0.316 | 0.7721 |
| Stha_C | 0.004 ± 0.146 | -0.081 ± 0.286 | 0.8140 |
| The *p* value with label ‘*’ meant the result was significant after Bonferroni correction (p < 0.05/30). | | | |

| **Table S23. Postoperative variations of Nodal local efficiency between fast-recovery and slow-recovery groups (left lesion)** | | | |
| --- | --- | --- | --- |
| Nodal | Fast-recovery | Slow-recovery | *p* value  (Two-sample *t* test) |
| A6dl_C | 0.050 ± 0.027 | 0.085 ± 0.043 | 0.4943 |
| A6m_C | -0.002 ± 0.029 | 0.023 ± 0.031 | 0.5770 |
| A6vl_C | -0.013 ± 0.028 | -0.052 ± 0.038 | 0.4235 |
| A6cdl_C | 0.017 ± 0.012 | 0.043 ± 0.018 | 0.2500 |
| A4ul_L | -0.051 ± 0.027 | 0.093 ± 0.033 | 0.0027 |
| A4ul_C | 0.011 ± 0.022 | -0.022 ± 0.022 | 0.3262 |
| A4t_L | 0.063 ± 0.044 | 0.106 ± 0.065 | 0.5870 |
| A4t_C | 0.010 ± 0.023 | -0.011 ± 0.027 | 0.5731 |
| A4tl_L | -0.001 ± 0.040 | -0.002 ± 0.042 | 0.9830 |
| A4tl_C | 0.017 ± 0.029 | 0.022 ± 0.042 | 0.9342 |
| A6cvl_L | 0.066 ± 0.060 | -0.024 ± 0.053 | 0.2984 |
| A6cvl_C | -0.076 ± 0.058 | -0.056 ± 0.032 | 0.7880 |
| A1_2_3ll_C | 0.015 ± 0.032 | -0.040 ± 0.050 | 0.3599 |
| A4ll_C | 0.041 ± 0.029 | -0.010 ± 0.054 | 0.3982 |
| A1_2_3ulhf_L | 0.042 ± 0.044 | 0.002 ± 0.048 | 0.5515 |
| A1_2_3ulhf_C | 0.023 ± 0.032 | 0.013 ± 0.030 | 0.8322 |
| A1_2_3tonIa_L | -0.047 ± 0.041 | -0.056 ± 0.034 | 0.8706 |
| A1_2_3tonIa_C | -0.004 ± 0.033 | 0.032 ± 0.062 | 0.6004 |
| A2_L | 0.046 ± 0.049 | 0.010 ± 0.048 | 0.6169 |
| A2_C | 0.018 ± 0.041 | -0.045 ± 0.028 | 0.2624 |
| A1_2_3tru_L | 0.025 ± 0.035 | 0.023 ± 0.034 | 0.9743 |
| A1_2_3tru_C | 0.040 ± 0.029 | -0.010 ± 0.032 | 0.2779 |
| A24cd_L | -0.063 ± 0.050 | 0.076 ± 0.055 | 0.0824 |
| A24cd_C | -0.008 ± 0.054 | -0.014 ± 0.053 | 0.9464 |
| A23c_L | 0.003 ± 0.037 | 0.030 ± 0.050 | 0.6711 |
| A23c_C | 0.011 ± 0.044 | -0.058 ± 0.033 | 0.2582 |
| mPMtha_L | 0.082 ± 0.053 | 0.074 ± 0.057 | 0.9204 |
| mPMtha_C | -0.062 ± 0.057 | 0.058 ± 0.057 | 0.1676 |
| Stha_L | -0.011 ± 0.065 | -0.053 ± 0.031 | 0.6127 |
| Stha_C | -0.096 ± 0.058 | 0.045 ± 0.053 | 0.1053 |
| The *p* value with label ‘*’ meant the result was significant after Bonferroni correction (p < 0.05/30). | | | |

| **Table S24. Postoperative variations of Nodal local efficiency between fast-recovery and slow-recovery groups (right lesion)** | | | |
| --- | --- | --- | --- |
| Nodal | Fast-recovery | Slow-recovery | *p* value  (Two-sample *t* test) |
| A6dl_C | -0.009 ± 0.032 | -0.108 ± 0.060 | 0.2169 |
| A6m_C | 0.011 ± 0.017 | -0.037 ± 0.038 | 0.3116 |
| A6vl_C | -0.054 ± 0.039 | -0.002 ± 0.048 | 0.4640 |
| A6cdl_C | -0.069 ± 0.032 | -0.020 ± 0.032 | 0.3403 |
| A4ul_L | -0.018 ± 0.023 | -0.005 ± 0.029 | 0.7562 |
| A4ul_C | -0.162 ± 0.051 | -0.022 ± 0.032 | 0.0590 |
| A4t_L | -0.102 ± 0.046 | 0.042 ± 0.058 | 0.1074 |
| A4t_C | 0.073 ± 0.041 | 0.041 ± 0.061 | 0.6974 |
| A4tl_L | 0.000 ± 0.000 | 0.098 ± 0.069 | 0.2250 |
| A4tl_C | 0.021 ± 0.041 | 0.071 ± 0.041 | 0.4538 |
| A6cvl_L | 0.080 ± 0.062 | 0.056 ± 0.046 | 0.7887 |
| A6cvl_C | -0.190 ± 0.055 | -0.009 ± 0.039 | 0.0343 |
| A1_2_3ll_C | 0.185 ± 0.037 | 0.051 ± 0.095 | 0.2574 |
| A4ll_C | -0.059 ± 0.054 | 0.034 ± 0.038 | 0.2275 |
| A1_2_3ulhf_L | 0.033 ± 0.037 | 0.035 ± 0.038 | 0.9656 |
| A1_2_3ulhf_C | -0.110 ± 0.032 | 0.040 ± 0.028 | 0.0097 |
| A1_2_3tonIa_L | 0.000 ± 0.000 | 0.035 ± 0.080 | 0.6963 |
| A1_2_3tonIa_C | 0.219 ± 0.081 | 0.085 ± 0.046 | 0.2197 |
| A2_L | -0.056 ± 0.103 | -0.015 ± 0.081 | 0.7795 |
| A2_C | -0.011 ± 0.039 | -0.006 ± 0.036 | 0.9366 |
| A1_2_3tru_L | 0.002 ± 0.037 | 0.046 ± 0.045 | 0.5088 |
| A1_2_3tru_C | -0.068 ± 0.026 | 0.024 ± 0.042 | 0.1255 |
| A24cd_L | 0.055 ± 0.071 | -0.029 ± 0.069 | 0.4570 |
| A24cd_C | 0.043 ± 0.091 | -0.032 ± 0.021 | 0.4774 |
| A23c_L | -0.016 ± 0.050 | -0.040 ± 0.075 | 0.8154 |
| A23c_C | -0.034 ± 0.070 | 0.104 ± 0.063 | 0.2136 |
| mPMtha_L | -0.187 ± 0.102 | -0.107 ± 0.052 | 0.5369 |
| mPMtha_C | -0.041 ± 0.069 | -0.049 ± 0.078 | 0.9494 |
| Stha_L | 0.033 ± 0.090 | 0.124 ± 0.104 | 0.5577 |
| Stha_C | 0.005 ± 0.010 | -0.078 ± 0.098 | 0.4626 |
| The *p* value with label ‘*’ meant the result was significant after Bonferroni correction (p < 0.05/30). | | | |

| **Table S25. Postoperative variations of Nodal betweenness between fast-recovery and slow-recovery groups (left lesion)** | | | | |
| --- | --- | --- | --- | --- |
| Nodal | Fast-recovery | Slow-recovery | | *p* value  (Two-sample *t* test) |
| A6dl_C | -15.706 ± 8.164 | | -24.385 ± 13.376 | 0.5798 |
| A6m_C | -12.000 ± 14.729 | | 0.308 ± 12.038 | 0.5542 |
| A6vl_C | 0.529 ± 1.162 | | 4.462 ± 4.136 | 0.3327 |
| A6cdl_C | 4.471 ± 12.316 | | -33.462 ± 12.605 | 0.0499 |
| A4ul_L | 20.824 ± 17.978 | | -21.846 ± 17.703 | 0.1204 |
| A4ul_C | -4.235 ± 9.313 | | 7.923 ± 7.622 | 0.3578 |
| A4t_L | -30.941 ± 14.602 | | -5.846 ± 9.055 | 0.2002 |
| A4t_C | 4.176 ± 20.863 | | 7.231 ± 16.424 | 0.9164 |
| A4tl_L | -2.412 ± 2.865 | | -0.077 ± 2.774 | 0.5845 |
| A4tl_C | -0.118 ± 3.082 | | 0.385 ± 4.583 | 0.9282 |
| A6cvl_L | 1.176 ± 4.235 | | -14.923 ± 9.303 | 0.1116 |
| A6cvl_C | 6.353 ± 4.731 | | 6.385 ± 6.679 | 0.9970 |
| A1_2_3ll_C | -3.706 ± 6.327 | | 4.692 ± 17.195 | 0.6306 |
| A4ll_C | -8.824 ± 9.295 | | 11.308 ± 11.889 | 0.2015 |
| A1_2_3ulhf_L | -5.824 ± 10.727 | | -9.769 ± 9.374 | 0.7980 |
| A1_2_3ulhf_C | -7.000 ± 7.671 | | -6.923 ± 6.525 | 0.9944 |
| A1_2_3tonIa_L | -1.588 ± 4.009 | | 2.154 ± 3.435 | 0.5149 |
| A1_2_3tonIa_C | -6.000 ± 2.424 | | -0.846 ± 4.217 | 0.2897 |
| A2_L | -5.588 ± 7.015 | | 6.615 ± 10.141 | 0.3326 |
| A2_C | 2.647 ± 4.831 | | -0.308 ± 8.111 | 0.7532 |
| A1_2_3tru_L | -14.471 ± 14.972 | | 13.231 ± 18.663 | 0.2675 |
| A1_2_3tru_C | -7.765 ± 8.787 | | 5.385 ± 18.005 | 0.5020 |
| A24cd_L | -0.588 ± 2.437 | | 3.077 ± 1.325 | 0.2506 |
| A24cd_C | -0.588 ± 4.784 | | -5.462 ± 3.410 | 0.4562 |
| A23c_L | 5.588 ± 4.062 | | -12.923 ± 7.533 | 0.0348 |
| A23c_C | -2.882 ± 8.562 | | 4.077 ± 7.402 | 0.5713 |
| mPMtha_L | -1.824 ± 4.541 | | 0.923 ± 1.298 | 0.6216 |
| mPMtha_C | -3.118 ± 4.914 | | -7.385 ± 4.791 | 0.5612 |
| Stha_L | 0.059 ± 0.057 | | 1.923 ± 1.701 | 0.2365 |
| Stha_C | -0.647 ± 4.656 | | -0.154 ± 2.363 | 0.9341 |
| The *p* value with label ‘*’ meant the result was significant after Bonferroni correction (p < 0.05/30). | | | | |

| **Table S26. Postoperative variations of Nodal betweenness between fast-recovery and slow-recovery groups (right lesion)** | | | | |
| --- | --- | --- | --- | --- |
| Nodal | Fast-recovery | Slow-recovery | | *p* value  (Two-sample *t* test) |
| A6dl_C | -8.278 ± 9.718 | | -1.500 ± 22.038 | 0.8025 |
| A6m_C | -17.911 ± 16.375 | | 6.000 ± 15.098 | 0.3502 |
| A6vl_C | -0.167 ± 2.241 | | -6.333 ± 6.785 | 0.4491 |
| A6cdl_C | -1.033 ± 7.363 | | 4.833 ± 22.387 | 0.8248 |
| A4ul_L | -25.100 ± 10.294 | | 30.833 ± 19.454 | 0.0428 |
| A4ul_C | 70.511 ± 17.045 | | -3.000 ± 20.535 | 0.0307 |
| A4t_L | 16.722 ± 18.018 | | 7.167 ± 10.553 | 0.6850 |
| A4t_C | -30.089 ± 13.279 | | -24.000 ± 24.867 | 0.8476 |
| A4tl_L | -0.500 ± 0.456 | | 0.833 ± 0.955 | 0.2769 |
| A4tl_C | 3.544 ± 2.129 | | -4.000 ± 6.557 | 0.3414 |
| A6cvl_L | -15.856 ± 7.106 | | -5.667 ± 10.373 | 0.4765 |
| A6cvl_C | -34.067 ± 13.456 | | -28.667 ± 10.702 | 0.7802 |
| A1_2_3ll_C | -9.111 ± 4.122 | | 12.833 ± 15.819 | 0.2485 |
| A4ll_C | 61.522 ± 47.505 | | -74.500 ± 32.744 | 0.0569 |
| A1_2_3ulhf_L | 15.989 ± 8.235 | | 5.333 ± 12.197 | 0.5236 |
| A1_2_3ulhf_C | 32.989 ± 10.337 | | -10.833 ± 7.316 | 0.0102 |
| A1_2_3tonIa_L | 0.167 ± 0.597 | | 4.000 ± 4.203 | 0.4290 |
| A1_2_3tonIa_C | -0.333 ± 0.304 | | 12.167 ± 7.373 | 0.1530 |
| A2_L | -0.333 ± 0.304 | | 7.500 ± 6.315 | 0.2844 |
| A2_C | -5.567 ± 5.068 | | 18.333 ± 11.127 | 0.1047 |
| A1_2_3tru_L | 30.400 ± 12.607 | | 14.667 ± 17.424 | 0.5194 |
| A1_2_3tru_C | -31.467 ± 10.224 | | 23.167 ± 33.857 | 0.1888 |
| A24cd_L | 16.789 ± 10.487 | | 9.000 ± 7.885 | 0.5997 |
| A24cd_C | 17.578 ± 5.297 | | 4.667 ± 4.080 | 0.1084 |
| A23c_L | 12.067 ± 10.145 | | 26.500 ± 14.466 | 0.4730 |
| A23c_C | 4.333 ± 4.724 | | -10.000 ± 10.293 | 0.2748 |
| mPMtha_L | 2.500 ± 2.282 | | -4.333 ± 5.481 | 0.3181 |
| mPMtha_C | 19.178 ± 8.693 | | 28.500 ± 21.929 | 0.7258 |
| Stha_L | 0.500 ± 0.456 | | -3.667 ± 5.631 | 0.5160 |
| Stha_C | -4.333 ± 3.956 | | -0.333 ± 6.367 | 0.6367 |
| The *p* value with label ‘*’ meant the result was significant after Bonferroni correction (p < 0.05/30). | | | | |

| **Table S27. Postoperative variations of Nodal clustering coefficient between fast-recovery and slow-recovery groups (left lesion)** | | | |
| --- | --- | --- | --- |
| Nodal | Fast-recovery | Slow-recovery | *p* value  (Two-sample *t* test) |
| A6dl_C | 0.063 ± 0.038 | 0.138 ± 0.066 | 0.3297 |
| A6m_C | -0.014 ± 0.040 | -0.026 ± 0.062 | 0.8694 |
| A6vl_C | -0.048 ± 0.048 | -0.098 ± 0.076 | 0.5789 |
| A6cdl_C | 0.020 ± 0.017 | 0.061 ± 0.028 | 0.2193 |
| A4ul_L | -0.097 ± 0.029 | 0.108 ± 0.048 | 0.0008* |
| A4ul_C | 0.045 ± 0.034 | -0.042 ± 0.036 | 0.1044 |
| A4t_L | 0.093 ± 0.059 | 0.130 ± 0.096 | 0.7402 |
| A4t_C | 0.013 ± 0.027 | -0.019 ± 0.031 | 0.4632 |
| A4tl_L | 0.017 ± 0.061 | 0.007 ± 0.075 | 0.9205 |
| A4tl_C | 0.040 ± 0.045 | 0.040 ± 0.069 | 0.9944 |
| A6cvl_L | 0.104 ± 0.106 | -0.069 ± 0.097 | 0.2670 |
| A6cvl_C | -0.155 ± 0.088 | -0.121 ± 0.059 | 0.7790 |
| A1_2_3ll_C | 0.046 ± 0.043 | -0.063 ± 0.091 | 0.2689 |
| A4ll_C | 0.068 ± 0.047 | -0.042 ± 0.092 | 0.2819 |
| A1_2_3ulhf_L | 0.083 ± 0.072 | -0.007 ± 0.081 | 0.4278 |
| A1_2_3ulhf_C | 0.048 ± 0.055 | 0.002 ± 0.048 | 0.5661 |
| A1_2_3tonIa_L | -0.054 ± 0.068 | -0.080 ± 0.052 | 0.7792 |
| A1_2_3tonIa_C | -0.006 ± 0.040 | 0.043 ± 0.101 | 0.6373 |
| A2_L | 0.085 ± 0.072 | -0.001 ± 0.083 | 0.4551 |
| A2_C | 0.038 ± 0.070 | -0.112 ± 0.059 | 0.1383 |
| A1_2_3tru_L | 0.008 ± 0.040 | 0.014 ± 0.037 | 0.9205 |
| A1_2_3tru_C | 0.044 ± 0.030 | -0.020 ± 0.040 | 0.2178 |
| A24cd_L | -0.115 ± 0.084 | 0.105 ± 0.079 | 0.0834 |
| A24cd_C | 0.007 ± 0.091 | -0.011 ± 0.080 | 0.8890 |
| A23c_L | -0.008 ± 0.068 | 0.044 ± 0.077 | 0.6298 |
| A23c_C | 0.039 ± 0.065 | -0.099 ± 0.052 | 0.1384 |
| mPMtha_L | 0.115 ± 0.093 | 0.185 ± 0.090 | 0.6135 |
| mPMtha_C | -0.082 ± 0.102 | 0.121 ± 0.094 | 0.1805 |
| Stha_L | -0.042 ± 0.115 | -0.057 ± 0.061 | 0.9184 |
| Stha_C | -0.139 ± 0.094 | 0.024 ± 0.087 | 0.2400 |
| The *p* value with label ‘*’ meant the result was significant after Bonferroni correction (p < 0.05/30). | | | |

| **Table S28. Postoperative variations of Nodal clustering coefficient between fast-recovery and slow-recovery groups (right lesion)** | | | |
| --- | --- | --- | --- |
| Nodal | Fast-recovery | Slow-recovery | *p* value  (Two-sample *t* test) |
| A6dl_C | -0.052 ± 0.033 | -0.202 ± 0.120 | 0.2944 |
| A6m_C | 0.048 ± 0.045 | -0.046 ± 0.065 | 0.3032 |
| A6vl_C | -0.095 ± 0.090 | -0.027 ± 0.094 | 0.6438 |
| A6cdl_C | -0.147 ± 0.051 | -0.036 ± 0.035 | 0.1288 |
| A4ul_L | -0.010 ± 0.043 | -0.015 ± 0.026 | 0.9265 |
| A4ul_C | -0.330 ± 0.091 | -0.009 ± 0.038 | 0.0141 |
| A4t_L | -0.280 ± 0.101 | 0.058 ± 0.086 | 0.0418 |
| A4t_C | 0.106 ± 0.064 | 0.063 ± 0.074 | 0.6981 |
| A4tl_L | 0.000 ± 0.000 | 0.168 ± 0.116 | 0.2139 |
| A4tl_C | 0.060 ± 0.079 | 0.119 ± 0.069 | 0.6221 |
| A6cvl_L | 0.267 ± 0.138 | 0.066 ± 0.043 | 0.2328 |
| A6cvl_C | -0.324 ± 0.079 | 0.054 ± 0.114 | 0.0324 |
| A1_2_3ll_C | 0.217 ± 0.050 | 0.069 ± 0.178 | 0.4822 |
| A4ll_C | -0.106 ± 0.075 | 0.072 ± 0.052 | 0.1054 |
| A1_2_3ulhf_L | 0.092 ± 0.065 | 0.088 ± 0.074 | 0.9712 |
| A1_2_3ulhf_C | -0.221 ± 0.040 | 0.074 ± 0.082 | 0.0142 |
| A1_2_3tonIa_L | 0.000 ± 0.000 | 0.039 ± 0.130 | 0.7872 |
| A1_2_3tonIa_C | 0.375 ± 0.148 | 0.125 ± 0.064 | 0.1863 |
| A2_L | -0.058 ± 0.157 | 0.010 ± 0.144 | 0.7749 |
| A2_C | 0.070 ± 0.104 | 0.017 ± 0.066 | 0.7033 |
| A1_2_3tru_L | 0.000 ± 0.053 | 0.058 ± 0.064 | 0.5399 |
| A1_2_3tru_C | -0.058 ± 0.022 | 0.054 ± 0.079 | 0.2425 |
| A24cd_L | 0.112 ± 0.138 | -0.089 ± 0.126 | 0.3503 |
| A24cd_C | 0.097 ± 0.151 | -0.059 ± 0.034 | 0.3781 |
| A23c_L | -0.093 ± 0.071 | -0.081 ± 0.128 | 0.9387 |
| A23c_C | -0.081 ± 0.117 | 0.061 ± 0.099 | 0.4176 |
| mPMtha_L | -0.348 ± 0.185 | -0.161 ± 0.079 | 0.4171 |
| mPMtha_C | -0.039 ± 0.096 | -0.050 ± 0.142 | 0.9532 |
| Stha_L | 0.013 ± 0.180 | 0.214 ± 0.206 | 0.5179 |
| Stha_C | 0.057 ± 0.025 | -0.143 ± 0.178 | 0.3318 |
| The *p* value with label ‘*’ meant the result was significant after Bonferroni correction (p < 0.05/30). | | | |

| **Table S29. Postoperative variations of Nodal vulnerability between fast-recovery and slow-recovery groups (left lesion)** | | | |
| --- | --- | --- | --- |
| Nodal | Fast-recovery | Slow-recovery | *p* value  (Two-sample *t* test) |
| A6dl_C | -0.010 ± 0.011 | -0.034 ± 0.021 | 0.2897 |
| A6m_C | -0.022 ± 0.021 | -0.014 ± 0.019 | 0.8077 |
| A6vl_C | -0.003 ± 0.002 | 0.003 ± 0.005 | 0.1943 |
| A6cdl_C | -0.005 ± 0.014 | -0.045 ± 0.016 | 0.0800 |
| A4ul_L | 0.048 ± 0.028 | -0.066 ± 0.027 | 0.0104 |
| A4ul_C | -0.009 ± 0.008 | 0.010 ± 0.008 | 0.1203 |
| A4t_L | -0.051 ± 0.021 | -0.007 ± 0.014 | 0.1322 |
| A4t_C | -0.005 ± 0.027 | -0.008 ± 0.019 | 0.9375 |
| A4tl_L | -0.003 ± 0.007 | 0.000 ± 0.011 | 0.8047 |
| A4tl_C | -0.003 ± 0.009 | 0.008 ± 0.009 | 0.3786 |
| A6cvl_L | 0.005 ± 0.011 | -0.038 ± 0.021 | 0.0811 |
| A6cvl_C | -0.003 ± 0.008 | 0.011 ± 0.013 | 0.3936 |
| A1_2_3ll_C | -0.002 ± 0.006 | 0.004 ± 0.027 | 0.8144 |
| A4ll_C | -0.024 ± 0.018 | 0.008 ± 0.011 | 0.1907 |
| A1_2_3ulhf_L | -0.004 ± 0.016 | 0.001 ± 0.018 | 0.8225 |
| A1_2_3ulhf_C | -0.008 ± 0.011 | -0.006 ± 0.014 | 0.8811 |
| A1_2_3tonIa_L | -0.002 ± 0.011 | 0.008 ± 0.009 | 0.5210 |
| A1_2_3tonIa_C | -0.016 ± 0.008 | 0.018 ± 0.013 | 0.0247 |
| A2_L | 0.005 ± 0.013 | 0.002 ± 0.013 | 0.8867 |
| A2_C | 0.011 ± 0.007 | 0.002 ± 0.012 | 0.4914 |
| A1_2_3tru_L | -0.025 ± 0.027 | 0.024 ± 0.032 | 0.2654 |
| A1_2_3tru_C | -0.001 ± 0.008 | 0.010 ± 0.012 | 0.4618 |
| A24cd_L | 0.000 ± 0.008 | 0.008 ± 0.008 | 0.5144 |
| A24cd_C | -0.009 ± 0.011 | -0.003 ± 0.005 | 0.6721 |
| A23c_L | 0.018 ± 0.016 | -0.011 ± 0.017 | 0.2407 |
| A23c_C | -0.015 ± 0.013 | 0.010 ± 0.010 | 0.1845 |
| mPMtha_L | 0.002 ± 0.010 | 0.001 ± 0.008 | 0.9439 |
| mPMtha_C | -0.006 ± 0.009 | -0.009 ± 0.012 | 0.8391 |
| Stha_L | 0.006 ± 0.010 | -0.009 ± 0.012 | 0.3635 |
| Stha_C | -0.004 ± 0.010 | 0.003 ± 0.005 | 0.5944 |
| The *p* value with label ‘*’ meant the result was significant after Bonferroni correction (p < 0.05/30). | | | |

| **Table S30. Postoperative variations of vulnerability between fast-recovery and slow-recovery groups (right lesion)** | | | |
| --- | --- | --- | --- |
| Nodal | Fast-recovery | Slow-recovery | *p* value  (Two-sample *t* test) |
| A6dl_C | -0.074 ± 0.032 | -0.075 ± 0.044 | 0.9827 |
| A6m_C | -0.042 ± 0.035 | 0.007 ± 0.018 | 0.2875 |
| A6vl_C | 0.000 ± 0.002 | 0.000 ± 0.003 | 0.9664 |
| A6cdl_C | 0.009 ± 0.007 | -0.015 ± 0.034 | 0.5476 |
| A4ul_L | -0.016 ± 0.005 | 0.027 ± 0.050 | 0.4634 |
| A4ul_C | 0.036 ± 0.009 | 0.033 ± 0.017 | 0.8787 |
| A4t_L | 0.026 ± 0.012 | -0.030 ± 0.039 | 0.2384 |
| A4t_C | -0.097 ± 0.014 | 0.016 ± 0.082 | 0.2409 |
| A4tl_L | -0.007 ± 0.003 | 0.002 ± 0.004 | 0.1007 |
| A4tl_C | -0.004 ± 0.002 | -0.012 ± 0.008 | 0.4310 |
| A6cvl_L | -0.042 ± 0.022 | -0.007 ± 0.009 | 0.2049 |
| A6cvl_C | -0.066 ± 0.026 | -0.044 ± 0.013 | 0.5162 |
| A1_2_3ll_C | 0.000 ± 0.005 | 0.008 ± 0.010 | 0.5561 |
| A4ll_C | 0.017 ± 0.022 | -0.128 ± 0.076 | 0.1247 |
| A1_2_3ulhf_L | 0.030 ± 0.015 | 0.026 ± 0.012 | 0.8805 |
| A1_2_3ulhf_C | 0.048 ± 0.019 | 0.008 ± 0.017 | 0.1848 |
| A1_2_3tonIa_L | 0.026 ± 0.010 | 0.009 ± 0.007 | 0.2176 |
| A1_2_3tonIa_C | -0.002 ± 0.002 | 0.022 ± 0.013 | 0.1276 |
| A2_L | -0.007 ± 0.004 | 0.016 ± 0.011 | 0.0995 |
| A2_C | -0.019 ± 0.010 | 0.012 ± 0.011 | 0.0780 |
| A1_2_3tru_L | 0.048 ± 0.036 | 0.017 ± 0.020 | 0.5116 |
| A1_2_3tru_C | -0.011 ± 0.004 | 0.004 ± 0.054 | 0.8051 |
| A24cd_L | 0.035 ± 0.019 | 0.011 ± 0.013 | 0.3494 |
| A24cd_C | 0.046 ± 0.015 | 0.024 ± 0.011 | 0.2824 |
| A23c_L | 0.010 ± 0.009 | 0.031 ± 0.017 | 0.3592 |
| A23c_C | -0.024 ± 0.016 | -0.029 ± 0.012 | 0.8055 |
| mPMtha_L | 0.004 ± 0.005 | -0.009 ± 0.005 | 0.1281 |
| mPMtha_C | -0.004 ± 0.007 | 0.040 ± 0.034 | 0.2750 |
| Stha_L | 0.000 ± 0.002 | -0.001 ± 0.008 | 0.8751 |
| Stha_C | -0.001 ± 0.003 | -0.012 ± 0.020 | 0.6276 |
| The *p* value with label ‘*’ meant the result was significant after Bonferroni correction (p < 0.05/30). | | | |

**Table S31. The mediation effect of postoperative nodal efficiency of A4ul_L between** ***d_CST_* and recovery time**

|  | Effect | SE or Boot SE | t value | *p* value | Lower 95% CI | Upper 95% CI | Percentage of effect |
| --- | --- | --- | --- | --- | --- | --- | --- |
| Correlated to postoperative nodal efficiency of A4ul_L | | | |  |  |  |  |
| Constant | -0.6159 | 0.2271 | -2.7116 | 0.0098 | -1.075 | -0.1568 | - |
| *d_CST_* | 0.0769 | 0.0226 | 3.4072 | 0.0015 | 0.0313 | 0.1225 | - |
| Direct effect |  |  |  |  |  |  |  |
| Constant | 19.0710 | 3.6832 | 5.1778 | < 0.0001 | 11.6208 | 26.5211 | - |
| *d_CST_* | -0.4100 | 0.3820 | -1.0735 | 0.2896 | -1.1826 | 0.3625 | - |
| Nodal efficiency of A4ul_L | -9.7165 | 2.3565 | -4.1233 | 0.0002 | -14.483 | -4.9500 | - |
| Total effect |  |  |  |  |  |  |  |
| Constant | 25.0555 | 4.0055 | 6.2553 | < 0.0001 | 16.96 | 33.151 | - |
| *d_CST_* | -1.1570 | 0.3979 | -2.9079 | 0.0059 | -1.9611 | -0.3528 | - |
| Summary |  |  |  |  |  |  |  |
| Total effect | -1.1570 | 0.3979 | -2.9079 | 0.0059 | -1.9611 | -0.3528 | - |
| Direct effect | -0.4100 | 0.3820 | -1.0735 | 0.2896 | -1.1826 | 0.3625 | 35.44% |
| Indirect effect | -0.7470 | 0.3301 |  | < 0.05 | -1.4848 | -0.1858 | 64.56% |

* A4ul_L, lesioned-hemispheric Brodmam area 4 upper limb region. CI, confidential interval. *d_CST_* shortest distance from surgical region to corticospinal tract.

**Table S32. The mediation effect of postoperative nodal degree centrality of A4ul_L between** ***d_CST_* and recovery time**

|  | Effect | SE or Boot SE | t value | *p* value | Lower 95% CI | Upper 95% CI | Percentage of effect |
| --- | --- | --- | --- | --- | --- | --- | --- |
| Correlated to postoperative nodal degree centrality of A4ul_L | | | |  |  |  |  |
| Constant | -0.5418 | 0.2345 | -2.3109 | 0.0261 | -1.0157 | -0.0679 | - |
| *d_CST_* | 0.0676 | 0.0233 | 2.9037 | 0.0060 | 0.0206 | 0.1147 | - |
| Direct effect |  |  |  |  |  |  |  |
| Constant | 21.9065 | 4.0612 | 5.3941 | < 0.0001 | 13.6918 | 30.1211 | - |
| *d_CST_* | -0.7640 | 0.4169 | -1.8323 | 0.0745 | -1.6073 | 0.0794 | - |
| Nodal degree centrality of A4ul_L | -5.8117 | 2.5723 | -2.2594 | 0.0295 | -11.0148 | -0.6087 | - |
| Total effect |  |  |  |  |  |  |  |
| Constant | 25.0555 | 4.0055 | 6.2553 | < 0.0001 | 16.9600 | 33.1510 | - |
| *d_CST_* | -1.1570 | 0.3979 | -2.9079 | 0.0059 | -1.9611 | -0.3528 | - |
| Summary |  |  |  |  |  |  |  |
| Total effect | -1.1570 | 0.3979 | -2.9079 | 0.0059 | -1.9611 | -0.3528 | - |
| Direct effect | -0.7640 | 0.4169 | -1.8323 | 0.0745 | -1.6073 | 0.0794 | 66.03% |
| Indirect effect | -0.3930 | 0.2052 |  | < 0.05 | -0.8606 | -0.0614 | 33.97% |

* A4ul_L, lesioned-hemispheric Brodmam area 4 upper limb region. CI, confidential interval. *d_CST_* shortest distance from surgical region to corticospinal tract.

**Table S33. The mediation effect of postoperative nodal vulnerability of A4ul_L between** ***d_CST_* and recovery time**

|  | Effect | SE or Boot SE | t value | *p* value | Lower 95% CI | Upper 95% CI | Percentage of effect |
| --- | --- | --- | --- | --- | --- | --- | --- |
| Correlated to postoperative nodal vulnerability of A4ul_L | | | |  |  |  |  |
| Constant | -0.3930 | 0.2459 | -1.5980 | 0.1179 | -0.8900 | 0.1040 | - |
| *d_CST_* | 0.0490 | 0.0244 | 2.0080 | 0.0514 | -0.0003 | 0.0984 | - |
| Direct effect |  |  |  |  |  |  |  |
| Constant | 23.2435 | 4.0129 | 5.7923 | < 0.0001 | 15.1266 | 31.3604 | - |
| *d_CST_* | -0.9308 | 0.4055 | -2.2957 | 0.0272 | -1.7510 | -0.1107 | - |
| Nodal vulnerability of A4ul_L | -4.6110 | 2.5016 | -1.8432 | 0.0729 | -9.6710 | 0.4490 | - |
| Total effect |  |  |  |  |  |  |  |
| Constant | 25.0555 | 4.0055 | 6.2553 | < 0.0001 | 16.9600 | 33.1510 | - |
| *d_CST_* | -1.1570 | 0.3979 | -2.9079 | 0.0059 | -1.9611 | -0.3528 | - |
| Summary |  |  |  |  |  |  |  |
| Total effect | -1.1570 | 0.3979 | -2.9079 | 0.0059 | -1.9611 | -0.3528 | - |
| Direct effect | -0.9308 | 0.4055 | -2.2957 | 0.0272 | -1.7510 | -0.1107 | 80.45% |
| Indirect effect | -0.2262 | 0.1875 |  | ＞0.05 | -0.6809 | 0.0261 | 19.55% |

* A4ul_L, lesioned-hemispheric Brodmam area 4 upper limb region. CI, confidential interval. *d_CST_* shortest distance from surgical region to corticospinal tract.

**Table S34. The mediation effect of variations of nodal efficiency of A4ul_L between** ***d_CST_* and recovery time**

|  | Effect | SE or Boot SE | t value | *p* value | Lower 95% CI | Upper 95% CI | Percentage of effect |
| --- | --- | --- | --- | --- | --- | --- | --- |
| Correlated to variations of nodal efficiency of A4ul_L | | | |  |  |  |  |
| Constant | -0.3890 | 0.2462 | -1.5803 | 0.1219 | -0.8865 | 0.1085 | - |
| *d_CST_* | 0.0486 | 0.0245 | 1.9857 | 0.0540 | -0.0009 | 0.0980 | - |
| Direct effect |  |  |  |  |  |  |  |
| Constant | 21.1305 | 3.2803 | 6.4416 | < 0.0001 | 14.4953 | 27.7656 | - |
| *d_CST_* | -0.6671 | 0.3313 | -2.0133 | 0.0510 | -1.3373 | 0.0031 | - |
| Variations of nodal efficiency | -10.0901 | 2.0442 | -4.9359 | < 0.0001 | -14.2250 | -5.9552 | - |
| Total effect |  |  |  |  |  |  |  |
| Constant | 25.0555 | 4.0055 | 6.2553 | < 0.0001 | 16.9600 | 33.1510 | - |
| *d_CST_* | -1.1570 | 0.3979 | -2.9079 | 0.0059 | -1.9611 | -0.3528 | - |
| Summary |  |  |  |  |  |  |  |
| Total effect | -1.1570 | 0.3979 | -2.9079 | 0.0059 | -1.9611 | -0.3528 | - |
| Direct effect | -0.6671 | 0.3313 | -2.0133 | 0.0510 | -1.3373 | 0.0031 | 57.66% |
| Indirect effect | -0.4899 | 0.2676 |  | < 0.05 | -1.0722 | -0.0290 | 42.34% |

* A4ul_L, lesioned-hemispheric Brodmam area 4 upper limb region. CI, confidential interval. *d_CST_* shortest distance from surgical region to corticospinal tract.

**Table S35. The mediation effect of variations of nodal degree centrality of A4ul_L between** ***d_CST_* and recovery time**

|  | Effect | SE or Boot SE | t value | *p* value | Lower 95% CI | Upper 95% CI | Percentage of effect |
| --- | --- | --- | --- | --- | --- | --- | --- |
| Correlated to variations of nodal degree centrality of A4ul_L | | | |  |  |  |  |
| Constant | -0.5108 | 0.2372 | -2.1536 | 0.0374 | -0.9903 | -0.0314 | - |
| *d_CST_* | 0.0638 | 0.0236 | 2.7061 | 0.0100 | 0.0161 | 0.1114 | - |
| Direct effect |  |  |  |  |  |  |  |
| Constant | 20.8876 | 3.7519 | 5.5672 | < 0.0001 | 13.2986 | 28.4767 | - |
| *d_CST_* | -0.6368 | 0.3837 | -1.6595 | 0.1050 | -1.4130 | 0.1394 | - |
| Variations of nodal degree centrality | -8.1587 | 2.3674 | -3.4462 | 0.0014 | -12.9474 | -3.3701 | - |
| Total effect |  |  |  |  |  |  |  |
| Constant | 25.0555 | 4.0055 | 6.2553 | < 0.0001 | 16.9600 | 33.1510 | - |
| *d_CST_* | -1.1570 | 0.3979 | -2.9079 | 0.0059 | -1.9611 | -0.3528 | - |
| Summary |  |  |  |  |  |  |  |
| Total effect | -1.1570 | 0.3979 | -2.9079 | 0.0059 | -1.9611 | -0.3528 | - |
| Direct effect | -0.6368 | 0.3837 | -1.6595 | 0.1050 | -1.4130 | 0.1394 | 55.04% |
| Indirect effect | -0.5202 | 0.3072 |  | < 0.05 | -1.3118 | -0.1053 | 44.96% |

* A4ul_L, lesioned-hemispheric Brodmam area 4 upper limb region. CI, confidential interval. *d_CST_* shortest distance from surgical region to corticospinal tract.

**Table S36. The mediation effect of variations of nodal vulnerability of A4ul_L between** ***d_CST_* and recovery time**

|  | Effect | SE or Boot SE | t value | *p* value | Lower 95% CI | Upper 95% CI | Percentage of effect |
| --- | --- | --- | --- | --- | --- | --- | --- |
| Correlated to variations of nodal vulnerability of A4ul_L | | | |  |  |  |  |
| Constant | -0.3972 | 0.2456 | -1.6170 | 0.1137 | -0.8937 | 0.0993 | - |
| *d_CST_* | 0.0496 | 0.0244 | 2.0318 | 0.0489 | 0.0003 | 0.0989 | - |
| Direct effect |  |  |  |  |  |  |  |
| Constant | 22.3357 | 3.7999 | 5.8779 | < 0.0001 | 14.6495 | 30.0220 | - |
| *d_CST_* | -0.8175 | 0.3841 | -2.1284 | 0.0397 | -1.5945 | -0.0406 | - |
| Variations of nodal vulnerability | -6.8474 | 2.3697 | -2.8895 | 0.0063 | -11.6407 | -2.0541 | - |
| Total effect |  |  |  |  |  |  |  |
| Constant | 25.0555 | 4.0055 | 6.2553 | < 0.0001 | 16.9600 | 33.1510 | - |
| *d_CST_* | -1.1570 | 0.3979 | -2.9079 | 0.0059 | -1.9611 | -0.3528 | - |
| Summary |  |  |  |  |  |  |  |
| Total effect | -1.1570 | 0.3979 | -2.9079 | 0.0059 | -1.9611 | -0.3528 | - |
| Direct effect | -0.8175 | 0.3841 | -2.1284 | 0.0397 | -1.5945 | -0.0406 | 70.66% |
| Indirect effect | -0.3395 | 0.1781 |  | < 0.05 | -0.7321 | -0.0406 | 29.34% |

* A4ul_L, lesioned-hemispheric Brodmam area 4 upper limb region. CI, confidential interval. *d_CST_* shortest distance from surgical region to corticospinal tract.
